# Recombinant Bile Salt-Stimulated Lipase in Preterm Infant Feeding: A Randomized Phase 3 Study

**DOI:** 10.1371/journal.pone.0156071

**Published:** 2016-05-31

**Authors:** Charlotte Casper, Jean-Michel Hascoet, Tibor Ertl, Janusz S. Gadzinowski, Virgilio Carnielli, Jacques Rigo, Alexandre Lapillonne, María L. Couce, Mårten Vågerö, Ingrid Palmgren, Kristina Timdahl, Olle Hernell

**Affiliations:** 1 Unit of Neonatology, Paul Sabatier University, Toulouse, France; 2 Department of Neonatology, Université de Lorraine, Nancy, France; 3 Department of Neonatology, Medical School, University of Pécs, Pécs, Hungary; 4 Chair and Department of Neonatology, Poznan University of Medical Sciences, Poznan, Poland; 5 Polytechnic University of Marche and Salesi’s Children Hospital, Ancona, Italy; 6 Department of Neonatology, University of Liège, Liège, Belgium; 7 Necker Enfants Malades Hospital, Paris Descartes University, EA 7328, Paris, France; 8 Neonatology Department, University Clinical Hospital, Santiago de Compostela, Spain; 9 Swedish Orphan Biovitrum (Sobi), Stockholm, Sweden; 10 Pediatrics, Department of Clinical Sciences, Umeå University, Umeå, Sweden; Cardiff University, UNITED KINGDOM

## Abstract

**Introduction:**

Feeding strategies are critical for healthy growth in preterm infants. Bile salt-stimulated lipase (BSSL), present in human milk, is important for fat digestion and absorption but is inactivated during pasteurization and absent in formula. This study evaluated if recombinant human BSSL (rhBSSL) improves growth in preterm infants when added to formula or pasteurized breast milk.

**Patients and Methods:**

LAIF (Lipase Added to Infant Feeding) was a randomized, double-blind, placebo-controlled phase 3 study in infants born before 32 weeks of gestation. The primary efficacy variable was growth velocity (g/kg/day) during 4 weeks intervention. Follow-up visits were at 3 and 12 months. The study was performed at 54 centers in 10 European countries.

**Results:**

In total 415 patients were randomized (rhBSSL n = 207, placebo n = 208), 410 patients were analyzed (rhBSSL n = 206, placebo n = 204) and 365 patients were followed until 12 months. Overall, there was no significantly improved growth velocity during rhBSSL treatment compared to placebo (16.77 vs. 16.56 g/kg/day, estimated difference 0.21 g/kg/day, 95% CI [-0.40; 0.83]), nor were secondary endpoints met. However, in a predefined subgroup, small for gestational age infants, there was a significant effect on growth in favor of rhBSSL during treatment. The incidence of adverse events was higher in the rhBSSL group during treatment.

**Conclusions:**

Although this study did not meet its primary endpoint, except in a subgroup of infants small for gestational age, and there was an imbalance in short-term safety, these data provide insights in nutrition, growth and development in preterm infants.

**Trial Registration:**

ClinicalTrials.gov NCT01413581

## Introduction

During the last decades major achievements in neonatal intensive care are illustrated by increasing survival rates. Surviving infants have a higher burden of morbidities that may affect growth, body composition, neurodevelopment, organ development and differentiation [1–3], which has become an increasingly important issue. A higher growth velocity during the neonatal intensive care unit (NICU) hospitalization is associated with positive effects on growth and neurodevelopment [1, 4].

Optimized feeding strategies to mimic intrauterine growth and minimize the risk of delayed development and morbidities are major challenges. The recommended enteral nutrition for both term and preterm newborns is the mother’s own fresh breast milk, however donor milk or formula is frequently used. While milk is rich in fat, accounting for approximately half its energy content, the protein content is too low for the rapidly growing preterm infant motivating the use of fortifiers [5]. Efficient utilization of the energy-rich fat may be hampered by gastrointestinal immaturity in this patient group, emphasizing the need for proper nutritional care [6].

Pasteurization of donor milk to prevent transmission of pathogens is routine in most NICUs. A complication of this is the denaturation of heat labile bioactive proteins such as the bile salt-stimulated lipase (BSSL, also known as carboxyl ester lipase or bile salt-dependent lipase) [7–9]. BSSL is secreted by the exocrine pancreas into the intestinal lumen in all species studied. In some species, notably humans, it is also secreted by the lactating mammary gland and is thus a constituent of the milk. Due to the immature exocrine pancreatic function at birth, the milk is the major source of BSSL for newborn breastfed infants [6], and pasteurization of mothers own milk reduces fat absorption and weight gain in preterm infants [10]. Studies *in vitro* [11] and in rodents [12], suggest that BSSL and pancreatic lipase related protein 2 (PLRP2) are key enzymes in neonatal fat digestion. Thus the destruction of milk BSSL upon pasteurization and its absence from formula motivated the development of recombinant human BSSL (rhBSSL) as a therapeutic strategy to improve qualitative growth and not only weight gain, in non-breastfed preterm infants.

In two preceding phase 2 studies, rhBSSL was well tolerated and significantly improved growth velocity of preterm infants receiving 1 week of treatment, administered orally in infant formula or pasteurized breast milk (PBM) [13]. In agreement with previous studies *in vitro* [14] the long-chain polyunsaturated fatty acids (LCPUFAs): docosahexaenoic acid (DHA) and arachidonic acid (AA), had significantly higher coefficient of absorption in the rhBSSL group than placebo.

This randomized, double-blind, placebo-controlled, phase 3 study was designed to demonstrate improved growth in preterm infants compared to placebo after administration of rhBSSL in infant formula or PBM during 4 weeks of treatment. It was key to find a common clinical study protocol where the feeding volume, type of formula and fortification-schedule were standardized in a way accepted and possible to follow by all 54 participating centers.

## Subjects and Methodology

### Subjects

Infants eligible for the study were born before week 32 of gestation and hospitalized in a NICU. Infants with birth weight above the 10th percentile for gestational age on the gender-specific intrauterine growth curve, were categorized as appropriate for gestational age (AGA) and infants below the 10th percentile, as small for gestational age (SGA) [15]. Enteral feeding of at least 100 mL/kg/day and an expectancy to remain on formula or PMB and not receive fresh breast milk the following 4 weeks was a prerequisite. Patients were enrolled to the protocol that was approved at the time of their enrollment. All patients had the possibility to enroll into the follow-up period, 24 months, if they wanted. All amendments were approved by the ethics committee if judged necessary by the ethics committee. See clinical study protocol for more details (S1 Text). The first patient was randomized in July 2011 and the last patient’s last visit was in August 2014.

The study was conducted according to ICH GCP guidelines and the Declaration of Helsinki and approved by the following Ethics Committees: Belgium; CHU de Liège—Comité d'Ethique, Czech Republic;Etická komise Fakultní nemocnice Olomouc, University Hospital Ethics Committee Hradec Králové, Etická komise Všeobecná fakultní nemocnice v Praze, Etická komise Krajská nemocnice T. Bati and Schváleno Etickou Komisi, France; CPP Ile de France 3 Hôpital Tarnier, Germany; Berlin Regional Office for Health and Social Affairs Ethics Committee of the State of Berlin, Hungary; Medical Research Council Ethics Committee for Clinical Pharmacology, Italy; Comitato Etico Azienda Ospedaliero Universitaria Ospedali Riuniti, Laforgia Comitato Etico Indipendente Azienda Ospedaliero-Universitaria “Policlinico Consorziale”, Comitato Etico Indipendente Azienda Ospedaliero- Universitaria “Ospedali Riuniti” di Foggia, Comitato di Etica Fondazione IRCCS “Cà Granda” Ospedale Maggiore Policlinico, Comitato Etico dell’Universita Cattolica del Sacro Cuore–Policlinico Universitario A. Gemelli and Comitato Etico per la Sperimentazione, Poland; Bioethics Committee at the Medical University of Karol Marcinkowski in Poznan, Russia; Ivanovo Scientific Research Institute for Maternity and Childhood and the Local Ethics Committee Office in Nizhniy Novgorod, Spain; Comité Ético de Investigación Clinica Hospita Universitario de Salamanca, Comité Ético de Investigación Clínica de Galicia (Sergas), Comité Coordinador de Ética de la Investigación Biomédica de Andalucía and Comité Ético de Investigación Clínica de Asturias, Sweden; Regional Ethical Review Board in Umeå. In addition to the ethical approvals, the study was also approved by the Clinical Study Manager, Medical Program Director, and Associate Director of Project Management.Written informed consent was provided by the guardians of each infant.

### Study design and intervention

This was a prospective, multicenter, randomized, placebo-controlled, double-blind, phase 3 study comparing rhBSSL treatment with placebo (*www.clinicaltrials.gov* registration no: NCT01413581). The primary objective was to demonstrate that rhBSSL improves growth in preterm infants compared to placebo when administered in infant formula or PBM. Secondary objectives included assessment of decreasing the risk of growth restriction, shortening time of hospital stay, improving early development, decreasing re-admittance to hospital, increasing the serum concentrations of DHA and AA, and to compare the safety and tolerability of rhBSSL treatment with placebo. An interactive voice response system (IVRS) was used for the randomization. A randomization schedule for IVRS was generated linking sequential patient randomization numbers to treatment codes. The randomization was stratified by feeding regimen (PBM/infant formula) and by size for gestational age category (SGA/AGA) to minimize treatment imbalance within subgroups (S1 Text). The randomization numbers were blocked, and within each block the same number of patients was allocated to each treatment group. Intervention started on the day of randomization or the day after. Follow-up visits were scheduled 3 months after treatment initiation and at 12 months corrected age. For infants with anti-drug antibodies (ADA) at 3 months, an additional visit was scheduled at 6 months.

### Feeding regimen and study drug

Each center chose one formula and one target volume (150–180 ml/kg/day) for all infants to be maintained throughout the study. Restrictions in formula concentrations of protein (2.8–4.1 g/100 kcal), carbohydrates (9.5–12.0 g/100 kcal) and lipids (4.4–6.0 g/100 kcal) were given. Formulas had to contain DHA and AA and not >40% of fat as medium-chain triglycerides (MCT). PBM fortifications were allowed according to predefined site-specific schedules and were maintained throughout the treatment.

rhBSSL (Sobi, Sweden) lipolytic activity [16] was measured using emulsified olive oil or para-nitrophenyl-butyrate as substrate [17] (unpublished data). Further characterization was done by testing bile salt dependency, heparin binding capacity, peptide mapping, N- and C-terminal sequencing, carbohydrate content assessment, circular dichroism, analytical ultra-centrifugation, chromatographic and electrophoresis techniques.

Reconstitution of 15 mg rhBSSL (8700 Units) in 1 mL sterile water, added to 100 mL feed (final concentration 0.15 g/L), was within physiological range. Placebo was identical to the investigational product, except for the active ingredient; it contained mannitol, glycine, sodium phosphate and pH adjuster.

### Growth assessment

The primary outcome measure, growth velocity (g/kg/day), was assessed by ≥3 measurements/week during treatment. After that, weight was recorded at least weekly until discharge and at follow-up visits. Body weight was measured using a standardized scale (SECA 717), length (cm) was measured from the crown to the heel using a preterm infant length board, head circumference (mm) was measured using a non-stretch measuring tape.

### Safety assessments

Serious adverse events (SAEs) were reported once the informed consent was signed and until the 12-month follow-up visit, non-serious adverse events (AEs) were reported from treatment initiation to the 3 months follow-up visit. It was up to each investigator to judge if a clinical sign or an AE was considered related to the study drug. An independent data safety monitoring board reviewed unblinded data when 10%, 30%, 50% and 75% of the patients had completed treatment. As part of the safety objective, neurodevelopment was assessed using Bayley Scales III [18] at 12 months corrected age. Another secondary safety variable was development of ADA.

### Statistical analysis

In total 410 patients were required to ensure 90% power in demonstrating improved growth for rhBSSL compared to placebo, assuming a true difference in growth velocity of 2.25 g/kg/day and a standard deviation (SD) of 7 g/kg/day (using a 2-sided test with significance level of 5%). Assumptions were based on previous studies [13]; the estimate of SD was increased by 30% to allow greater variability within the phase 3 study population.

Growth velocity during treatment was determined for each patient using a linear regression model with log (weight) as response and a predictor variable of time. Growth velocity for each patient was estimated as the slope arising from the regression model. An analysis of covariance model including factors for treatment, feeding regimen, and size for gestational age category, with baseline weight included as a covariate was used.

Body weight, feeding utilization, length and head circumference were analyzed using covariance models including factors for treatment, feeding regimen, and size for gestational age category, with the baseline value included as a covariate. For the comparison between groups, the point estimate, associated 95% CI, and p-value were calculated.

The binary endpoints, a weight below the 10^th^ percentile at 4 weeks and a growth velocity below 15 g/kg/day during treatment, were analyzed using logistic regression models with treatment, feeding regimen and size for gestational age category as explanatory variables.

A statistical analysis plan was finalized prior to database lock. Sample size calculation was performed using nQuery Advisor software Version 4.0 (Statistical Solutions Ltd, Ireland). All output was produced using SAS software (SAS Institute, NC, USA).

## Results

### Patient disposition and demographics

**Fig 1:**
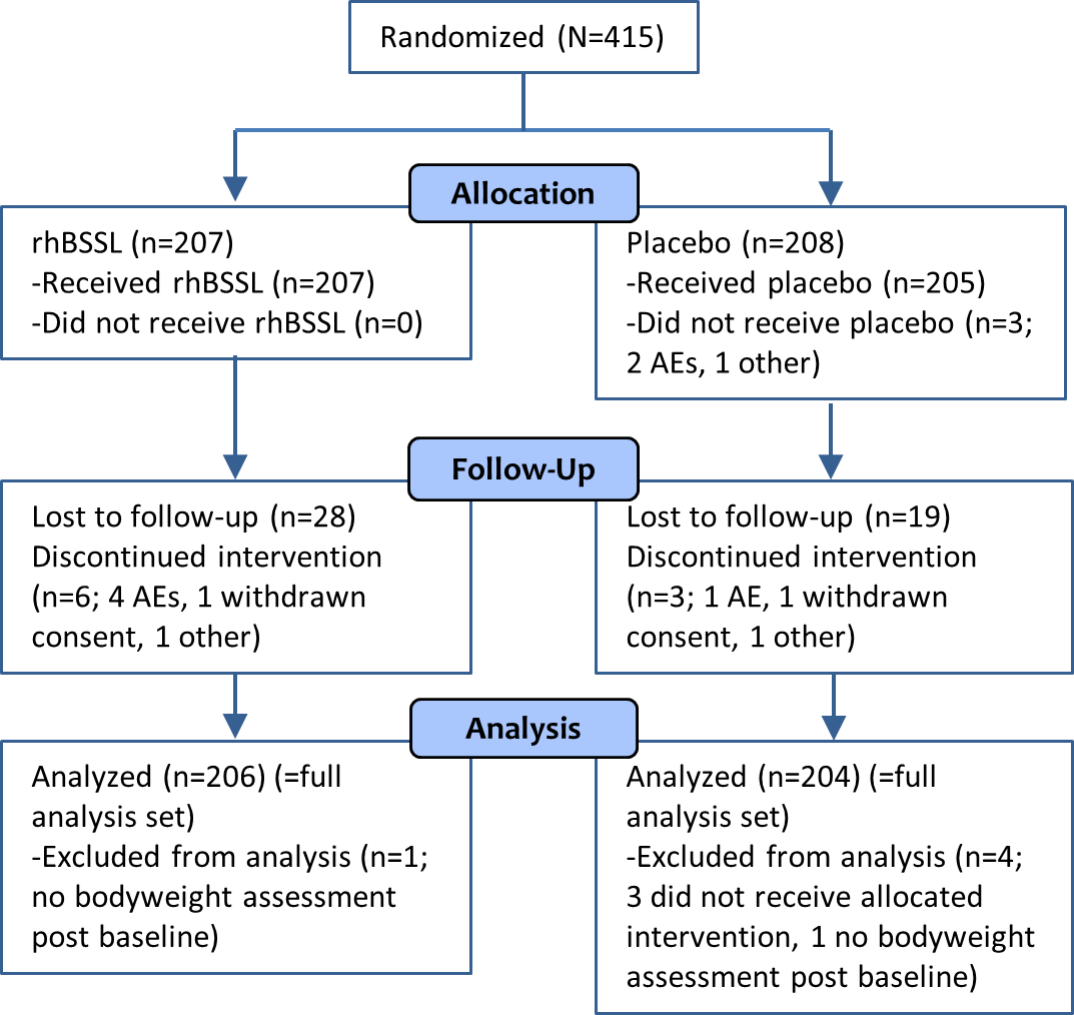
CONSORT flow diagram of randomized patients. AE; adverse event.

**Fig 2:**
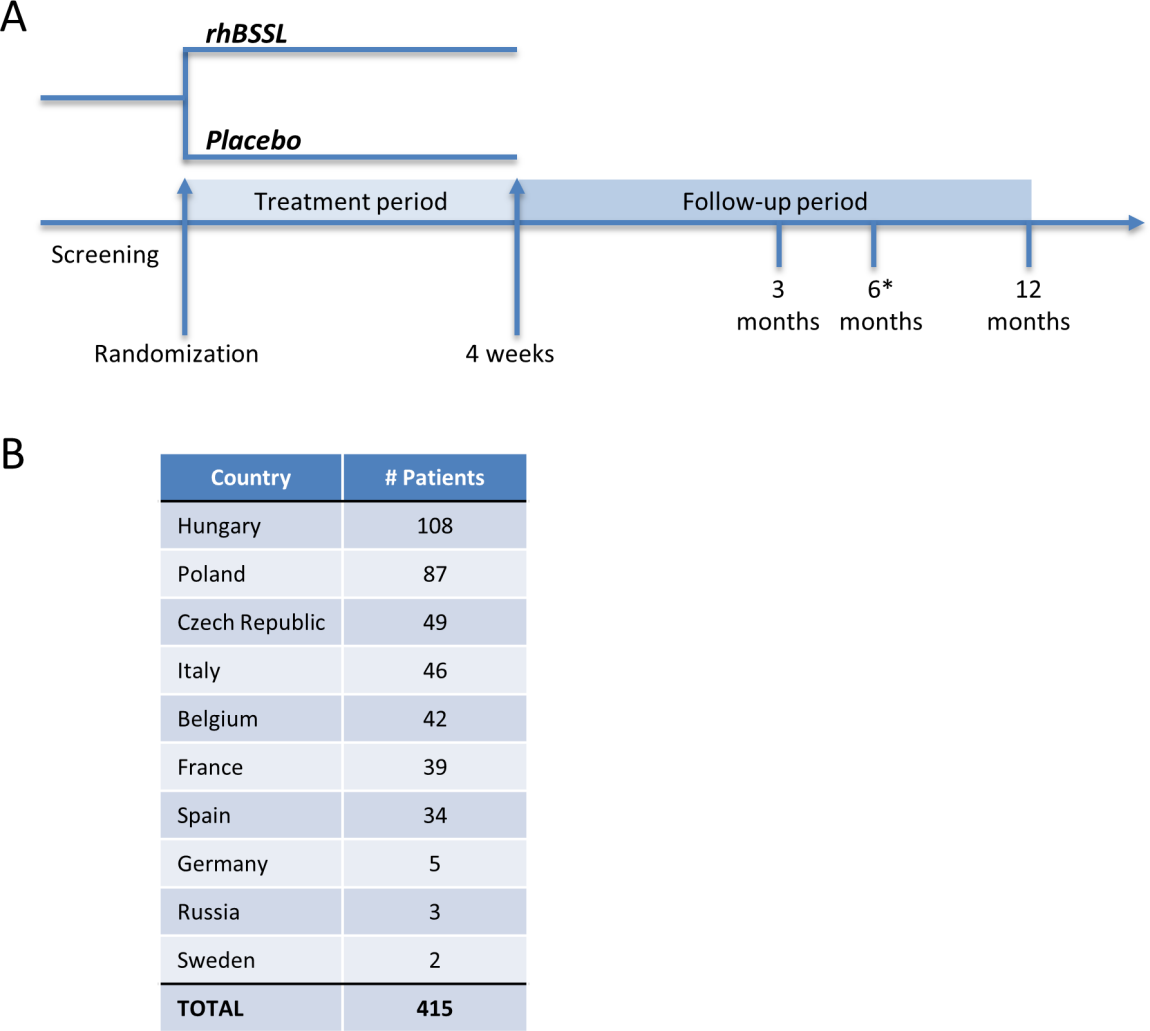
Clinical study design and number of patients. A) The LAIF study design. The asterisk indicates a visit specific for ADA positive infants at 3 months. B) Number of randomized patients.

A total of 415 preterm infants were randomized, 207 to rhBSSL treatment and 208 to placebo, 410 patients were included in the full analysis set (rhBSSL = 206, placebo = 204) (Fig 1). The 4 weeks intervention was completed by 98% of the patients and 88% completed the study until 12 months. The study was performed at 54 centers in 10 European countries (Fig 2).

Demographics were well balanced between groups (Table 1) with mean gestational age at birth of 28.8 weeks in both groups, age at randomization of 3.2 weeks in both groups, and a mean body weight at birth of 1179 g (rhBSSL) and 1167 g (placebo). There were slightly more females in the placebo group (57.4%) than in the rhBSSL group (50.5%). Most infants were formula-fed (rhBSSL 62%, placebo 63%). The small amounts of fresh breast milk that could not be prohibited for ethical reasons were balanced between groups and not considered confounding any efficacy results. The proportion of SGA infants was 16% in the rhBSSL group and 15% in the placebo group.

**Table 1:** Demographic characteristics (full analysis set^a^).

	rhBSSL (N = 206)	Placebo (N = 204)
**Sex**		
Male	102 (49.5%)	87 (42.6%)
Female	104 (50.5%)	117 (57.4%)
**Gestational age at birth (weeks)**		
Mean (SD)	28.8 (1.7)	28.8 (1.7)
Median (Min-Max)	28.9 (24.1–31.7)	29.0 (24.0–31.9)
**Age at randomization (weeks)**		
Mean (SD)	3.2 (1.5)	3.2 (1.5)
Median (Min-Max)	2.9 (1.0–8.0)	3.0 (1.0–8.4)
**Birth weight (g)**		
Mean (SD)	1179 (299)	1167 (294)
Median (Min-Max)	1170 (600–1850)	1165 (580–1990)
**Baseline weight**		
Mean (SD)	1385 (265)	1388 (263)
Median (Min-Max)	1386 (690–2090)	1388 (740–2130)
**Size for gestational age category**		
SGA	32 (16%)	30 (15%)
AGA	174 (84%)	174 (85%)
**Feeding regimen**		
Formula	127 (62%)	128 (63%)
PBM	79 (38%)	76 (37%)

^a^ Infants who received at least one dose (rhBSSL/placebo) and had a baseline and at least one post-baseline weight assessment.

Abbreviations: AGA; appropriate for gestational age, PBM; pasteurized breast milk, rhBSSL; recombinant human bile salt-stimulated lipase, SD; standard deviation, SGA; small for gestational age.

### rhBSSL did not significantly improve growth in preterm infants

**Fig 3:**
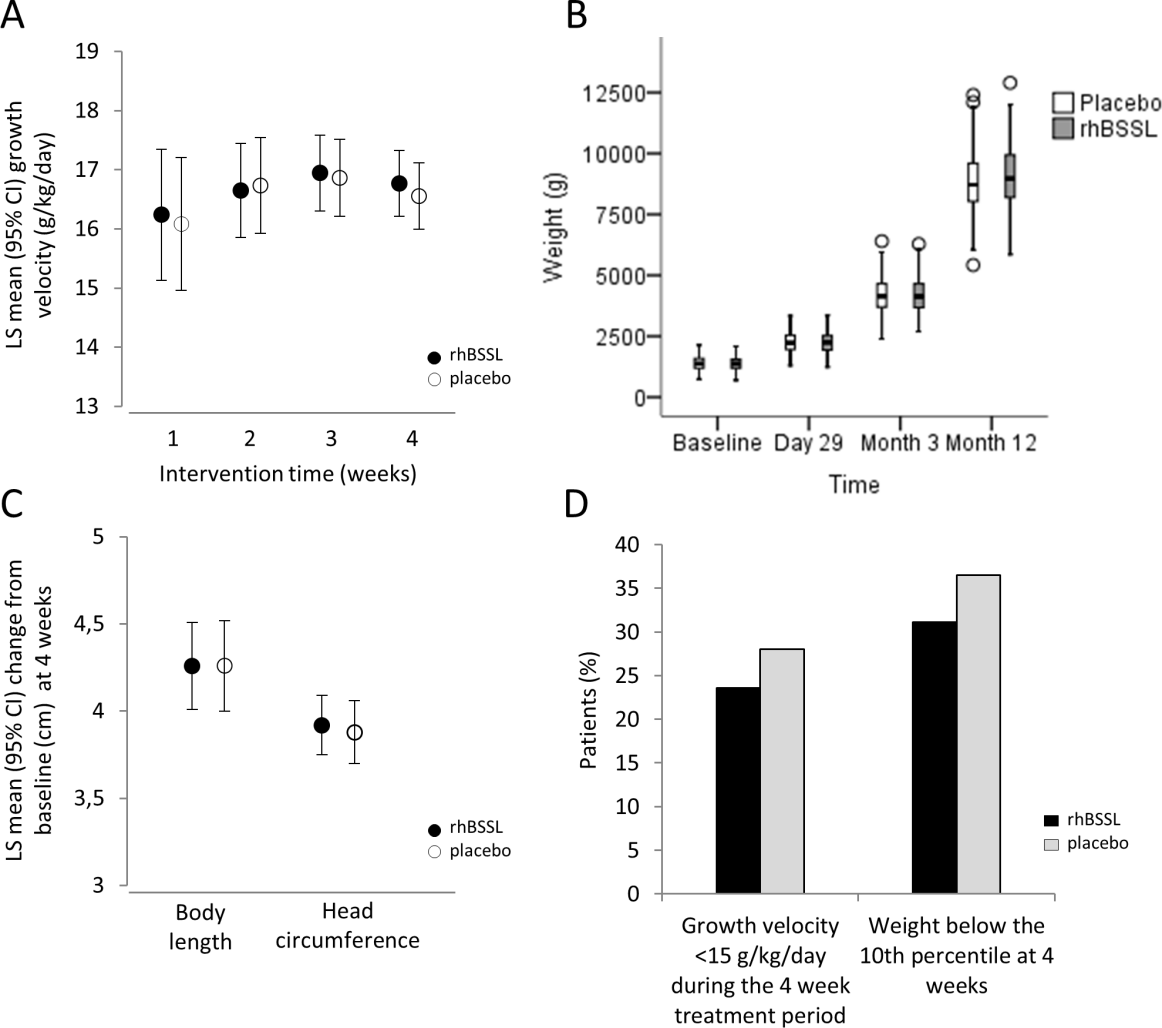
Treatment with rhBSSL does not improve growth in preterm infants. A) Growth velocity during intervention (rhBSSL; N = 206, placebo; N = 204). CI; confidence interval, LS mean; least square mean. B) Body weight at baseline, 4 weeks, 3 months, and 12 months adjusted age. Box plot illustrating median and Q1/Q3. Whiskers extend to lowest/highest value within 1.5 interquartile range from Q1/Q3 and outliers are indicated. C) Body length and head circumference; change from baseline at 4 weeks. D) Growth restriction during intervention.

The primary efficacy variable, growth velocity during 4 weeks intervention showed a mean of 16.77 g/kg/day for the rhBSSL group (N = 206) and a mean of 16.56 g/kg/day for the placebo group (N = 204) (Fig 3). The difference of 0.21 g/kg/day (95% CI [-0.40, 0.83]) in favor of rhBSSL was not statistically significant (p = 0.493), nor were differences observed after 1, 2 or 3 weeks of intervention significant. Growth velocity varied between countries, but the lack of any rhBSSL-effect was still consistent. There were no significant differences in the average body weight between groups throughout the study (Fig 3).

Body length and head circumference were similar between treatment groups with an LS mean difference (rhBSSL-placebo) of 0.00 cm (95% CI [-0.28, 0.27]) for body length and 0.04 cm (95% CI [-0.15, 0.24]) for head circumference (Fig 3). However, assessment of growth restriction, defined as growth velocity below 15g/kg/day or a body weight below the 10^th^ percentile at 4 weeks, showed a small numerical, albeit not statistically significant improvement in favor of rhBSSL (23.6 vs. 28.0%, p = 0.312 for growth velocity below 15 mg/kg/day and 31.1 vs. 36.5%, p = 0.340 for weight below the 10^th^ percentile) (Fig 3). In agreement with this, no significant improvement was observed for any of the additional secondary outcome measures in response to rhBSSL.

The mean (SD) feeding volume during intervention was 145.6 (17.81) ml/kg/day for rhBSSL and 149.6 (16.25) ml/kg/day for placebo (not shown). The reason for the 4 ml/kg/day lower mean feeding volume in the rhBSSL group remains unknown. Taking differences in feeding volume into account, calculations of feeding utilization, as defined by individual measures of weight gain per volume feed given during intervention, showed a statistically significant higher feeding utilization in the rhBSSL group. The improvement corresponded to a mean weight increase of approximately 0.65 g/kg/day for an infant receiving 150 ml/kg/day (not shown).

### Analyses of growth in preterm infant subgroups

**Fig 4:**
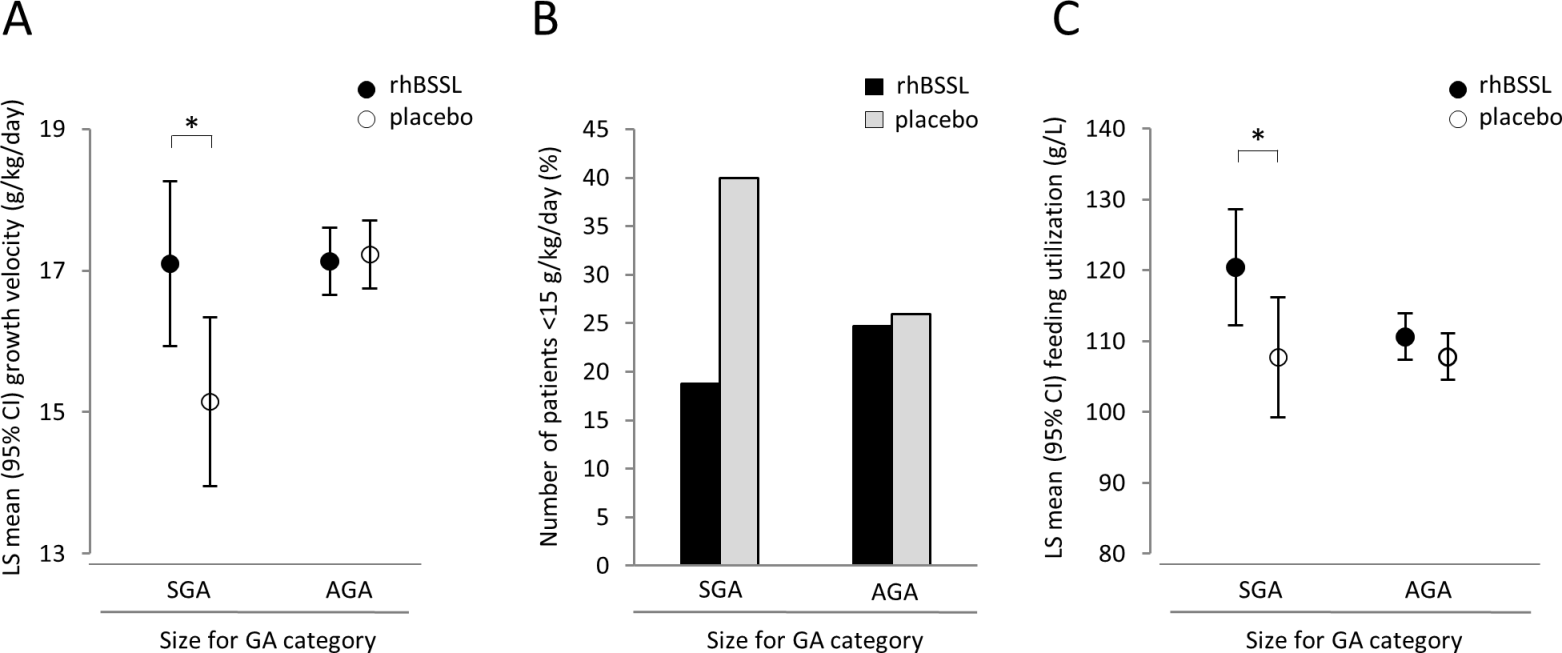
Treatment with rhBSSL improves growth in SGA infants during the 4 week treatment period. A) Growth velocity during intervention for SGA/AGA-infants. Asterisk indicates significance for SGA infants; LS mean difference (rhBSSL-placebo) of 1.95 (95% CI; 0.38, 3.52) and no significance for AGA infants; LS mean difference of -0.10 (95% CI; (-0.76, 0.57). B) Infants with a growth <15g/kg/day, odds ratio (95% CI) 0.321 (0.098, 1.045), p = 0.0592. C) Feeding utilization (LS mean rhBSSL: 120.36 g/L, LS mean placebo: 107.68 g/L). LS mean difference (95% CI): 12.68 (0.94, 24.42), p = 0.0347.

To examine any subgroup specific growth effects of rhBSSL treatment, prespecified analyses were performed by feeding regimen (PBM/formula), size for gestational age (SGA/AGA), gestational age at birth (<29 weeks, ≥29 weeks) or gender (male/female) (S1 Table). For SGA infants the mean growth velocity during treatment was 17.10 g/kg/day (n = 32) in the rhBSSL group, and 15.15 g/kg/day (n = 30) in the placebo group. Thus, the estimated difference was 1.95 g/kg/day, 95% CI [0.38, 3.52]), reaching statistical significance in favor of rhBSSL (Fig 4). Notably this effect was not driven by the smallest SGA infants. On the contrary, for AGA infants, no significant difference in growth velocity between rhBSSL (mean 17.13 g/kg/day) and placebo (mean 17.23 g/kg/day) was observed. Also, the interaction between treatment and size for gestational age category was statistically significant (p = 0.0187) indicating a different magnitude of improvement with rhBSSL versus placebo in SGA compared to AGA infants. Feeding regimen, feeding volume and the demographics of the SGA subgroup were similar to the overall population, except for birth weight (S2 Table). No significant growth advantage in response to rhBSSL was observed in any of the other subgroups.

In agreement with the positive growth effect of rhBSSL, fewer SGA infants on rhBSSL experienced growth below 15 g/kg/day (Fig 4). A minor decrease in the number of infants below the 10^th^ percentile in the rhBSSL group was also observed (not shown). However, these effects on growth restriction did not reach statistical significance.

SGA infants in the rhBSSL group experienced significantly higher feeding utilization than placebo, with a mean observed difference of 12.7 g/L (95% CI [0.94, 24.42], p = 0.0347) corresponding to 1.9 g/kg/day more weight increase per 150 ml/kg/day of feeding volume (Fig 4). There was no significant effect on feeding utilization in AGA infants indicating that the significance in the rhBSSL group in the total population was driven by the SGA infants.

Notably, independent of rhBSSL, the growth velocity was significantly higher among the formula-fed infants compared to those fed PBM (formula+rhBSSL 17.84 and formula+placebo 17.73 g/kg/day vs. PBM+rhBSSL 15.73 and PBM+placebo 15.35 g/kg/day (S1 Table). Head circumference and body length were also numerically larger in patients fed formula compared to PBM.

### Safety

The safety population included all 412 patients that received at least one dose of study medication (N = 212 rhBSSL, N = 200 placebo). The incidence of AEs and SAEs during intervention was higher in the rhBSSL group compared to placebo, 83% (rhBSSL) and 78% (placebo) experienced at least one AE and 9.4% (rhBSSL) and 6.5% (placebo) experienced at least one SAE (Table 2). The imbalance was due to more infections and gastrointestinal intolerability, including necrotizing enterocolitis (NEC) in the rhBSSL group, an imbalance that equalized during the follow-up period. One occurrence of NEC was in an SGA infant and the remaining 8 NEC-cases as well as one case of enterocolitis haemorrhagic occurred in AGA infants.

**Table 2:** Adverse events during the study period (safety population^a^).

Patients with at least one of the following (n [%]):	4 weeks treatment period		Post 4 weeks to 3 months		3 to 12 months	
	rhBSSL (N = 212)	Placebo (N = 200)	rhBSSL (N = 212)	Placebo (N = 200)	rhBSSL (N = 212)	Placebo (N = 200)
AE	176 (83.0)	156 (78.0)	128 (60.4)	116 (58.0)	NA^b^	NA^b^
SAE	20 (9.4)	13 (6.5)	46 (21.7)	44 (22.0)	61 (28.8)	55 (27.5)
AE leading to death	0	0	1 (0.5)	0	1	1
AE leading to study discontinuation	2 (0.9)	0	1 (0.5)	0	1 (0.5)	1 (0.5)
AE related to study drug^c^	8 (3.8)	3 (1.5)	0	1 (0.5)	NA	NA

^a^ All patients receiving at least one dose of study intervention (212 rhBSSL and 200 placebo) were included in the safety population. Five patients randomized to placebo were included in the rhBSSL group since they incorrectly had received ≥2 vials of rhBSSL.

^b^ Only SAEs were collected during the follow-up period (3 to 12 months).

^c^ Related to study drug, as judged by the investigator.

Abbreviations: AE; adverse event, NA; not applicable, rhBSSL; recombinant human bile salt-stimulated lipase, SAE; serious adverse event.

Two patients discontinued the study due to AEs during intervention, both on rhBSSL treatment. In total there were three deaths, all during the follow-up period, one during 4 weeks to 3 months (unknown reason; rhBSSL) and two during 3 to 12 months (respiratory failure; rhBSSL, circulatory collapse; placebo).

Abdominal distension, flatulence and gastroesophageal reflux disease were more frequent than other gastrointestinal intolerability AEs during rhBSSL treatment (Table 3). Despite this imbalance, there were no major differences in the proportion of patients with withheld feeding for 24 hours between groups (Table 4).

**Table 3:** Adverse events associated with gastrointestinal intolerability and necrotizing enterocolitis during the 4 week treatment period (safety population^a^).

	AEs associated with gastrointestinal intolerability and necrotizing enterocolitis			
	rhBSSL (N = 212)		Placebo (N = 200)	
	AEs	AEs qualifying as SAE	AEs	AEs qualifying as SAE
**Gastrointestinal intolerability, PT, n (%)**	**35 (16.5)**	**2 (0.9)**	**14 (7.0)**	**0**
Abdominal distension	7 (3.3)	0	1 (0.5)	0
Flatulence	7 (3.3)^c^	0	3 (1.5)	0
Gastrooesophageal reflux disease	7 (3.3)	0	5 (2.5)	0
Constipation	4 (1.9)^b^	0	1 (0.5)^b^	0
Infantile colic	3 (1.4)	0	0	0
Diarrhoea	2 (0.9)	1 (0.5)	2 (1.0)^b^	0
Abdominal pain	2 (0.9)^b^	0	0	0
Gastroenteritis	2 (0.9)	0	0	0
Haematochezia	2 (0.9)^b^	1 (0.5)	0	0
Regurgitation	2 (0.9)	0	0	0
Food intolerance	1 (0.5)	0	1 (0.5)	0
Vomiting	1 (0.5)	1 (0.5)	0	0
Enterocolitis	0	0	1 (0.5)^b^	0
**Necrotizing enterocolitis, PT, n (%)**	**8 (3.8)**	**5 (2.4)**	**1 (0.5)**	**1 (0.5)**
Necrotizing enterocolitis neonatal	7 (3.3)^c^	4 (1.9)	1 (0.5)	1 (0.5)
Enterocolitis haemorrhagic	1 (0.5)^b^	1 (0.5)	0	0

^a^ All patients receiving at least one dose of study intervention (212 rhBSSL and 200 placebo) were included in the safety population. Five patients randomized to placebo were included in the rhBSSL group since they incorrectly had received ≥2 vials of rhBSSL.

^b^ Including one report of treatment emergent AE that was considered drug related

^c^ Including two reports of treatment emergent AEs that was considered drug related

Note: A patient is only counted once per PT but may contribute to more than one PT in case of multiple events.

Abbreviations: AE; adverse event, PT; preferred term, rhBSSL; recombinant human bile salt-stimulated lipase, SAE; serious adverse event.

**Table 4:** Withheld enteral feeding for at least 24 hours (safety population^a^).

	rhBSSL (N = 212) n (%)	Placebo (N = 200) n (%)
**Number of patients where feeding was withheld during a 24 hour period**	21 (9.9)	19 (9.5)

^a^ Five patients randomized to placebo were included in the rhBSSL group since they incorrectly had received ≥2 vials of rhBSSL.

Note: Patients are only counted once, even if there are two or more breaks in 24 hour feeding during the treatment period.

Assessment of neurodevelopment was part of the safety objectives and performed using Bayley Scales III [18] at 12 months corrected age. The scales neither identified positive nor negative effects of rhBSSL treatment on neurodevelopment. Another safety endpoint was the assessment of ADAs, 16.5% of the total population had ADA at some point during the study, however there were no hypersensitivity reactions or events of NEC in patients with ADA (not shown).

## Discussion

In this large study in premature infants, we found that 4 weeks of rhBSSL treatment added to preterm formula or PBM did not improve growth velocity to a level where it reached statistical significance or clinical relevance. The reasons why previous phase 2 study results were not confirmed still remain elusive.

Extensive characterization to compare the inherent properties of the investigational product with the phase 2 product concluded that the enzymes had the same primary structure, comparable glycosylation profile, heparin binding capacity and *in vitro* activity. An alternative explanation for the unexpected results could be better growth in the placebo group in the phase 3 study, possibly concealing any rhBSSL-effect (phase 3: mean 15.35 g/kg/day for PBM and 17.73 g/kg/day for formula, phase 2: mean 13.63 g/kg/day for PBM and 14.31 g/kg/day for formula) [13]. This was possibly due to improved nutritional awareness and management in recent years.

PBM fortifications were frequent in both treatment groups and up to 40% of the fat content in formulas was allowed as MCTs, while only one single formula without MCTs was allowed in the phase 2 studies. However, the lack of efficacy could not be explained by differences in preplanned feeding regimen or addition of fortifications. Moreover, despite relatively wide protein-, carbohydrate- and lipid-composition allowed in the formulas, there were only minor differences in caloric intake between groups.

The rhBSSL-effect on growth in SGA infants indicates that the appropriate target population lies within a subpopulation. While it is often claimed that SGA infants require greater energy supply than AGA infants [19], there is no consensus on how to feed SGA infants, what the appropriate feeding volumes are, and what the rate of feeding advancement should be [20]. In this respect our study is consistent with Makrides et al. [21], showing that high DHA supplementation only met the outcome for some subgroups, i.e. girls or infants with a birth weight <1250 g, although the latter did not remain significant in adjusted analyses. One might speculate that rhBSSL may in part compensate for DHA and AA deficiency which has been demonstrated in SGA infants [22, 23]. The observed positive growth-effect of rhBSSL in SGA infants in our study needs to be confirmed, potentially in a larger group of SGA infants. The rational for including infants born before week 32 of gestation was based on previous experience from the phase 2 studies taking the level of pancreas immaturity into account. The exocrine pancreatic function is immature at birth and fat malabsorption is more common among preterm than term infants [6], and the key enzymes in fat digestion are different in early infancy than later in childhood. In rodents the exocrine pancreas seems to mature towards weaning [12]. The ontogeny has been less studied in infants but like in rodents expression of mRNA encoding colipase-dependent pancreatic lipase is low or undetectable at birth [24]. Exactly when the exocrine pancreas has matured to a level meeting the needs of all preterm or term infants remains to be studied. Possibly more immature or sick infants with more compromised digestion and absorption than studied here would benefit from BSSL supplementation.

The unexpected imbalance in safety profile between groups was mainly due to more infections and gastrointestinal intolerability, including NEC. One might speculate that certain unexpected effects generated in the intestinal lumen by rhBSSL, for unknown reason induced an inflammatory response. An expert panel re-evaluated the NEC diagnostic criteria and after re-assessment of clinical findings, biomarkers, x-rays, duration of withheld feeding and antibiotic treatment, 4 patients had NEC (3 rhBSSL, 1 placebo), 4 patients had suspected NEC (all rhBSSL), and 1 did not have NEC (rhBSSL). Study drug was stopped 2 to 10 days and then restarted, except in 2 patients who permanently stopped the study drug. All patients with NEC or suspected NEC recovered. Considering an estimated risk of NEC of up to 11% in very low birth weight infants [25], the incidence in this study was low (3.3% rhBSSL, 0.5% placebo), possibly due to investigators unconsciously proposing the study for infants with a relatively good intestinal tolerability.

These data substantially contribute to increased knowledge on feeding practice among preterm infants in Europe. Only 38% were fed PBM, although human milk is generally recommended [26], although the high proportion of formula-fed infants in part can be explained by exclusion of breastfed infants from the study. Milk composition varies greatly [27, 28] and was not measured and despite randomization we cannot exclude unbalanced lipid and energy content between groups.

It was encouraging that such a large study on this vulnerable patient population, in 10 European countries, could be conducted with high quality. Despite major variability in health care routines, we managed to unify the procedures in the clinical study protocol so that the variability after randomization was low with only small variations in mean volume intake over time. This is to our knowledge the first large double-blind study in preterm infants exploring a bioactive substance from breast milk and it is important to conduct further studies on such components from fresh breast milk and to optimize feeding protocols in order to improve nutrition and health in preterm infants.

## Supporting Information

S1 Table Growth velocity by feeding regimen, size for gestational age category, gestational age at birth, and sex (full analysis set). (DOCX)

S2 Table Demography and baseline characteristics for SGA patients (full analysis set). (DOCX)

S1 Text Clinical Study Protocol. (PDF)

S2 Text CONSORT Checklist. (DOC)

## Abbreviations

AA: arachidonic acid
ADA: anti-drug antibody
AE: adverse event
AGA: appropriate for gestational age
BSSL: bile salt-stimulated lipase
CI: confidence interval
DHA: docosahexaenoic acid
ICH GCP: International Conference on Harmonisation-Good Clinical Practice
IVRS: interactive voice response system
LCPUFA: long-chain polyunsaturated fatty acids
LS: least square
MCT: medium-chain triglycerides
NEC: necrotizing enterocolitis
NICU: neonatal intensive care unit
PBM: pasteurized breast milk
rhBSSL: recombinant human bile salt-stimulated lipase
SAE: serious adverse event
SD: standard deviation
SGA: small for gestational age
Sobi: Swedish Orphan Biovitrum
