# Supplementary material for: Recombinant Bile Salt-Stimulated Lipase in Preterm Infant Feeding: A Randomized Phase 3 Study
Source: PLoS One. 2016 May 31;11(5):e0156071. doi: 10.1371/journal.pone.0156071 (PMC4887005; doi:10.1371/journal.pone.0156071)
Supplement: S1 Table — (DOCX) [file pone.0156071.s001.docx]

**Supplementary Table 1.** Growth velocity by feeding regimen, size for gestational age category, gestational age at birth, and sex (full analysis set^a^)

|  |  | **rhBSSL (N=206)** | | **Placebo (N=204)** |
| --- | --- | --- | --- | --- |
| **Growth velocity by feeding regimen (g/kg/day)** | | | | |
| **PBM** | LS mean | 15.725 | | 15.354 |
|  | 95 % CI | (14.940, 16.511) | | (14.557, 16.152) |
| rhBSSL n=79 | LS mean (diff. rhBSSL-placebo) |  | 0.371 | |
| Placebo n=76 | 95 % CI |  | (-0.629, 1.370) | |
| **Formula** | LS mean | 17.844 | | 17.725 |
|  | 95 % CI | (17.202, 18.485) | | (17.082, 18.368) |
| rhBSSL n=127 | LS mean (diff. rhBSSL-placebo) |  | 0.119 | |
| Placebo n=128 | 95 % CI |  | (-0.660, 0.898) | |
| **Growth velocity by size for gestational age category (g/kg/day)** | | | | |
| **SGA** | LS mean | 17.097 | | 15.146 |
|  | 95 % CI | (15.935, 18.258) | | (13.954, 16.337) |
| rhBSSL n=32 | LS mean (diff. rhBSSL-placebo) |  | 1.951 | |
| Placebo n=30 | 95 % CI |  | (0.381, 3.521) | |
| **AGA** | LS mean | 17.131 | | 17.226 |
|  | 95 % CI | (16.653, 17.610) | | (16.748, 17.705) |
| rhBSSL n=174 | LS mean (diff. rhBSSL-placebo) |  | -0.095 | |
| Placebo n=174 | 95 % CI |  | (-0.757, 0.567) | |
| **Growth velocity by gestational age at birth (g/kg/day)** | | | | |
| **<29 weeks** | LS mean | 16.664 | | 16.639 |
|  | 95 % CI | (15.924, 17.404) | | (15.839, 17.440) |
| rhBSSL n=106 | LS mean (diff rhBSSL-placebo) |  | 0.025 | |
| Placebo n=97 | 95 % CI |  | (-0.853, 0.902) | |
| **≥29 weeks** | LS mean | 16.888 | | 16.486 |
|  | 95 % CI | (16.193, 17.582) | | (15.824, 17.147) |
| rhBSSL n=100 | LS mean (diff rhBSSL-placebo) |  | 0.402 | |
| Placebo n=107 | 95 % CI |  | (-0.465, 1.268) | |
| **Growth velocity by sex (g/kg/day)** | | | | |
| **Male** | LS mean | 16.627 | | 16.664 |
|  | 95 % CI | (15.931, 17.322) | | (15.937, 17.392) |
| rhBSSL n=102 | LS mean (diff. rhBSSL-placebo) |  | -0.038 | |
| Placebo n=87 | 95 % CI |  | (-0.947, 0.872) | |
| **Female** | LS mean | 16.905 | | 16.470 |
|  | 95 % CI | (16.178, 17.632) | | (15.759, 17.182) |
| rhBSSL n=104 | LS mean (diff. rhBSSL-placebo) |  | 0.435 | |
| Placebo n=117 | 95 % CI |  | (-0.405, 1.275) | |

^a^ Infants who received at least one dose (rhBSSL/placebo) and had a baseline and at least one post-baseline weight assessment.
Note: An analysis of covariance model including factors for treatment, feeding regimen (PBM or infant formula), size for gestational age category (SGA or AGA) and the interaction between treatment and the factor of interest (feeding, gestational age category, gestational age at birth and sex respectively), with baseline body weight included as a covariate was used. If a patient withdrew before 4 weeks then growth velocity was derived using weight assessments up to their last available assessment.
