# Supplementary material for: Recombinant Bile Salt-Stimulated Lipase in Preterm Infant Feeding: A Randomized Phase 3 Study
Source: PLoS One. 2016 May 31;11(5):e0156071. doi: 10.1371/journal.pone.0156071 (PMC4887005; doi:10.1371/journal.pone.0156071)
Supplement: S2 Table — (DOCX) [file pone.0156071.s002.docx]

**Supplementary Table 2**. Demography and baseline characteristics for SGA patients (full analysis set^a^)

|  | rhBSSL (N=32) | Placebo (N=30) | Total (N=62) |
| --- | --- | --- | --- |
| **Age at Randomization (weeks)** |  |  |  |
| Mean (SD) | 3.24 (1.61) | 3.04 (1.28) | 3.14 (1.45) |
| Median (Range) | 2.79 (1.00-7.86) | 2.79 (1.00-6.43) | 2.79 (1.00-7.86) |
| **Gestational age at Birth (weeks)** |  |  |  |
| Mean (SD) | 28.94 (1.61) | 29.27 (1.59) | 29.10 (1.59) |
| Median (Range) | 29.00 (25.00-31.29) | 29.50 (25.86-31.57) | 29.29 (25.00-31.57) |
| **Post-menstrual age at Randomization (weeks)** |  |  |  |
| Mean (SD) | 32.04 (0.93) | 32.17 (0.91) | 32.10 (0.92) |
| Median (Range) | 32.43 (29.57-32.86) | 32.50 (29.00-32.86) | 32.43 (29.00-32.86) |
| **Sex** |  |  |  |
| Male | 15 (46.9) | 17 (56.7) | 32 (51.6) |
| Female | 17 (53.1) | 13 (43.3) | 30 (48.4) |
| **Race** |  |  |  |
| White | 31 (96.9) | 30 (100.0) | 61 (98.4) |
| Asian | 1 (3.1) |  | 1 (1.6) |
| **Baseline Weight (g)** |  |  |  |
| Mean (SD) | 1091 (202) | 1106 (193) | 1098 (196) |
| Median (Range) | 1160 (690-1455) | 1088 (740-1500) | 1105 (690-1500) |
| **Feeding regimen** |  |  |  |
| Formula | 20 (62.5) | 18 (60.0) | 38 (61.3) |
| PBM | 12 (37.5) | 12 (40.0) | 24 (38.7) |

^a^ Infants who received at least one dose (rhBSSL/placebo) and had a baseline and at least one post-baseline weight assessment.
