# Supplementary material for: Recombinant Bile Salt-Stimulated Lipase in Preterm Infant Feeding: A Randomized Phase 3 Study
Source: PLoS One. 2016 May 31;11(5):e0156071. doi: 10.1371/journal.pone.0156071 (PMC4887005; doi:10.1371/journal.pone.0156071)
Supplement: S1 Text — (PDF) [file pone.0156071.s003.pdf]

CLINICAL STUDY PROTOCOL

A Prospective, Randomized, Double-Blind, Phase 3 Study Comparing rhBSSL and Placebo Added to Infant Formula or Pasteurized Breast Milk During 4 Weeks of Treatment in Preterm Infants Born Before Week 32 of Gestational Age

PROTOCOL NUMBER BVT.BSSL-030

**EudraCT Number:**	2010-023909-35
**Sponsor:**	Swedish Orphan Biovitrum AB (publ)<br>112 76 Stockholm, Sweden
**Sponsor Contact:**	Anna Olsson<br>Clinical Study Manager<br>Telephone: 46 8 697 20 00
**Sponsor's Medical Monitor**	Björn Paulsson MD<br>Medical Program Director<br>Telephone: 46 8 697 20 00
**Project Manager:**	Tracy Roe<br>Associate Director, Project Management<br>PPD Inc<br>Franklin House, Kings Worthy<br>Winchester, Hampshire, UK<br>Telephone: 44 7944 073 138
**PPD Medical Monitor:**	PPD Medical Monitor<br>PPD Granta Park<br>Great Abington,<br>Cambridge CB21 6GQ<br>United Kingdom<br>Telephone: PPD PVG Hotline: 44 1223 374240
**Version and Date of Protocol:**	Version 5.0 Including Administrative Change 2.0 –<br>10 June 2013

ETHICAL CONDUCT OF THE STUDY

The study will be conducted according to the International Conference on Harmonisation harmonised tripartite guideline E6(R1): Good Clinical Practice ensuring that the rights, safety and well-being of patients are protected, consistent with the principles that have their origin in the Declaration of Helsinki.

CONFIDENTIAL

The concepts and information contained in this document or generated during the study are considered proprietary and may not be disclosed in whole or in part without the expressed, written consent of Swedish Orphan Biovitrum AB (publ).

Table of Contents

Table of Contents 2
List of Tables 6
Protocol Approval 8
Declaration of Investigator 9
Summary of Changes 10
Protocol Synopsis 13
List of Abbreviations 20
Definitions of Terms 21
1 Introduction 22
1.1 Premature Infants and Growth 22
1.2 Pancreatic Function in Early Infancy 22
1.3 Bile-salt-stimulated Lipase 23
1.4 Recombinant Human BSSL 24
1.5 Completed Clinical Studies 24
1.6 Study Rationale 25
1.7 Potential Risks and Benefits 25
2 Study Objectives and Endpoints 26
2.1 Study Objectives 26
2.1.1 Primary Objective 26
2.1.2 Secondary Objectives 26
2.2 Exploratory Objectives 27
2.3 Study Endpoints 27
2.3.1 Primary Endpoint 27
2.3.2 Secondary Endpoints 27
2.3.3 Exploratory Endpoints 29
3 Investigational Plan 29
3.1 Study Design 29
3.2 Selection of Study Population 31

3.2.1 Inclusion Criteria 32
3.2.2 Exclusion Criteria 32
3.3 Withdrawal of Patients 33
3.3.1 Reasons for Withdrawal 33
3.3.2 Handling of Withdrawals 34
3.3.3 Replacements 34
3.4 Study Procedures 35
3.4.1 Medical History 35
3.4.2 Demography Assessments and Recording of Confounders of Neurodevelopment 35
3.4.2.1 Demography Assessments 35
3.4.2.2 Confounders of Neurodevelopment 36
3.4.3 Efficacy Assessments 37
3.4.3.1 Body Weight 37
3.4.3.2 Total Body Length 37
3.4.3.3 Total Body Height 38
3.4.3.4 Head Circumference 38
3.4.3.5 Time to Readiness for Discharge 38
3.4.3.6 Docosahexaenoic Acid (DHA) and Arachidonic Acid (AA) . 38
3.4.3.7 Bayley Scales of Infant and Toddler Development 39
3.4.3.8 Child Behavior Checklist 39
3.4.3.9 Neurodevelopment Disability Composite 40
3.4.4 Safety Assessments 40
3.4.4.1 Adverse Events 40
3.4.4.1.1 Definitions 40
3.4.4.1.2 Eliciting and Documenting Adverse Events 42
3.4.4.1.3 Reporting Adverse Events 43
3.4.4.1.4 Assessment of Severity 45
3.4.4.1.5 Assessment of Causality 46
3.4.4.1.6 Follow-up of Patients with Reported Adverse Events 47
3.4.4.1.6.1 Adverse Events Occurring before the 12-Months-corrected-age Visit 47
3.4.4.1.6.2 Serious Adverse Drug Reactions Occurring after the 12-Months-corrected-age Visit 47
3.4.4.2 Physical Examination 48

3.4.4.3 Gastrointestinal Tolerability 48
3.4.4.4 Vital Signs and Body Temperature 48
3.4.4.5 Electrocardiogram..... 48
3.4.4.6 Bayley Scales of Infant and Toddler Development 49
3.4.4.7 Laboratory Safety Analyses 50
3.4.4.7.1 Vitamins A and D 50
3.4.4.7.2 rhBSSL Antibodies 51
3.4.5 Exploratory Assessments 51
3.4.5.1 Total Fatty Acids 51
3.4.5.2 Body Composition 51
3.4.5.3 Calprotectin and Microbiota 51
3.4.6 Health Economic Assessments 52
3.4.6.1 Health Care Utilization 52
3.4.6.2 Indirect Resource Use 53
3.4.6.3 Chronic Medical Conditions/Diagnoses 53
3.4.7 Sample Collections 53
3.5 Overdose Management 55
3.6 Study Treatments 55
3.6.1 Method of Assigning Patients to Treatment Groups 55
3.6.2 Treatments Administered 55
3.6.3 Identity of Investigational Product 56
3.6.4 Management of Clinical Supplies 57
3.6.4.1 Study Drug Packaging and Storage 57
3.6.4.2 Preparation of Study Drug and Food 57
3.6.4.3 Study Drug Accountability 58
3.6.5 Food Requirements 58
3.6.5.1 Daily Volume of Enteral Feeding 58
3.6.5.2 Daily Volume of Parenteral Nutrition 58
3.6.5.3 Infant Formula Requirements 59
3.6.5.4 Pasteurized Breast Milk Requirements 59
3.6.5.5 Concomitant Use of Fresh Breast Milk 60
3.6.6 Blinding 60
3.6.7 Breaking the Blind 60
3.6.7.1 Emergency Unblinding 60
3.6.7.2 Scheduled Unblinding 61

3.6.8 Treatment Compliance 61
3.6.9 Prior, Concomitant, and Subsequent Therapy 61
3.7 Statistical Analysis Plan 62
3.7.1 Primary Efficacy Endpoints 62
3.7.2 Secondary Efficacy Endpoints 62
3.7.3 Safety Endpoints 64
3.7.4 Health Economic Endpoints 65
3.7.5 Exploratory Endpoints 66
3.7.6 Sample Size Calculations 66
3.7.7 Analysis Sets 66
3.8 Statistical Analysis 67
3.8.1 Primary Efficacy Analysis 67
3.8.2 Secondary Efficacy Analyses 68
3.8.3 Health Economic Endpoints 71
3.8.4 Exploratory Endpoints 71
3.8.5 Subgroup Analyses 71
3.8.6 Safety Analyses 71
3.8.6.1 Adverse Events 72
3.8.6.2 Clinical Laboratory Results 72
3.8.7 Interim Analyses 72
3.8.8 Analysis of Data up to 12-months 73
3.8.9 Analysis of 24-months Data 73
3.8.10 Combining Data 73
3.9 Data Quality Assurance 73
4 Investigator's Obligations 74
4.1 Confidentiality 74
4.2 Institutional Review 74
4.3 Informed Consent 75
4.4 Study Reporting Requirements 76
4.5 Financial Disclosure and Obligations 77
4.6 Investigator Documentation 77
4.7 Study Conduct 78
4.8 Data Collection 78

4.8.1 Case Report Forms and Source Documents 78
4.9 Adherence to Protocol....78
4.10 Coding Dictionaries 79
4.11 Investigator's Final Report 79
4.12 Records Retention....79
4.13 Publications....79
5 Study Management.... 81
5.1 Sponsor 81
5.2 Vendor Contact....81
5.3 Central Laboratories....82
5.4 Data Safety Monitoring Board....82
5.5 Monitoring 83
5.5.1 Monitoring of the Study....83
5.5.2 Inspection of Records 83
5.6 Management of Protocol Amendments and Deviations....84
5.6.1 Modification of the Protocol....84
5.6.2 Protocol Violations and Deviations 84
5.7 Study Termination....85
5.8 Final Report 85
6 Appendices 87
Appendix 1: Schedule of Events 87
Appendix 2: Protocol Amendment 1.0 92
Appendix 3: Administrative Change 1.0 102
Appendix 4: Protocol Amendment 2.0 109
Appendix 5: Administrative Change 2.0 126
7 Reference List.... 130

List of Tables

Table 1–1 Protocol Amendment History and Reasons for Amendment or Administrative Changes.... 10

Table 3–1 Maximum Blood Volumes to be Drawn from Each Patient (in Addition to Normal Routine Samples) During the Study Period 54

Table 3–2 Study Drug Supplies 56

Table 3–3 Composition of rhBSSL Drug Product 56

Table 6–1 Schedule of Events until Discharge 88

Table 6–2 Schedule of Events for Follow-up Visits 90

Protocol Approval

**Study Title**	A Prospective, Randomized, Double-Blind, Phase 3 Study Comparing rhBSSL and Placebo Added to Infant Formula or Pasteurized Breast Milk During 4 Weeks of Treatment in Preterm Infants Born Before Week 32 of Gestational Age
**Protocol Number**	BVT.BSSL-030
**Protocol Version and Date**	Version 5.0 Including Administrative Change 2.0 – 10 June 2013

Protocol accepted and approved by:

Anna Olsson
Clinical Study Manager
Swedish Orphan Biovitrum AB (publ)
112 76 Stockholm, Sweden

Anna Olsson ____________________ 11-Jun 2013
Signature Date

Björn Paulsson MD
Medical Program Director
Swedish Orphan Biovitrum AB (publ)
112 76 Stockholm, Sweden

Björn Paulsson ____________________ 11 June 2013
Signature Date

Tracy Roe
Associate Director, Project Management
PPD Inc
Franklin House, Kings Worthy
Winchester, Hampshire, UK

Tracy Roe ____________________ 11 June 2013
Signature Date

Declaration of Investigator

I have read and understand all sections of the protocol entitled "A Prospective, Randomized, Double-Blind, Phase 3 Study Comparing rhBSSL and Placebo Added to Infant Formula or Pasteurized Breast Milk During 4 Weeks of Treatment in Preterm Infants Born Before Week 32 of Gestational Age" and the accompanying current investigator's brochure.

I agree to supervise all aspects of the protocol and to conduct the clinical investigation in accordance with the Final Protocol, Version 5.0, including Administrative Change 2.0, 10 June 2013, the International Conference on Harmonisation harmonised tripartite guideline E6(R1): Good Clinical Practice, and all applicable government regulations. I will not make changes to the protocol before consulting with Swedish Orphan Biovitrum AB (publ), or implement protocol changes without independent ethics committee approval except to eliminate an immediate risk to patients. I agree to administer study drug only to patients under my personal supervision or the supervision of a subinvestigator.

I will not supply the investigational drug to any person not authorized to receive it. Confidentiality will be protected. Patient identity will not be disclosed to third parties or appear in any study reports or publications.

I will not disclose information regarding this clinical investigation or publish results of the investigation without authorization from Swedish Orphan Biovitrum AB (publ).

______________________________________ ____________________
Signature of Principal Investigator Date

____
Printed Name of Principal Investigator

Summary of Changes

Table 1–1 Protocol Amendment History and Reasons for Amendment or Administrative Changes

Version	Date	Reason for Amendment or Administrative Changes
Version 1.0	10 February 2011	Original Protocol
Version 2.0<br>Amendment 1.0	27 May 2011	• To change the assessment of the effect of rhBSSL treatment with respect to vitamin D from an exploratory endpoint to a secondary safety endpoint. The Vitamin D assessments were initially classified as exploratory due to uncertainty about the development of the bioanalytical method. The method has now been developed.<br>• To clarify:<br>o The reasons for withdrawal. There are no prohibited medications in the study, therefore the statement about withdrawal in the case of prohibited medication being used has been deleted.<br>o The collection of the demography assessment data. During the study, the equipment provided for length, weight and head circumference measurement is mandatory to use. Assessments performed before the patient has been included in the study cannot be controlled. Therefore the statement about recording the method used to obtain body weight at birth in the eCRF has been deleted.<br>o The reporting period for serious adverse events and tolerability assessments. Clarification that SAEs are collected from the time of signed informed consent. Tolerability will only be assessed during the treatment period and not weekly until discharge.<br>o The rhBSSL antibody sample collection schedule. The cut-off level for a positive antibody result will be based on baseline data from approximately the first 50 patients. As some patients might be due for the 6-month visit before the cut-off level has been defined this visit will initially be conducted for all patients irrespective of antibody results. Thereafter, the 6-month visit will only be conducted for those patients who have a positive result at the 3-month visit.<br>o The blind break procedure to be used with the interactive voice recognition system.<br>o How causality assessments will be reported in sponsor reports.<br>• To update the maximum blood volume to be drawn from each patient. Microtainers are only available in 0.5 mL and 0.25 mL. Therefore the total volume per visit has been increased by 0.05 mL (7% compared to the previous volume) at baseline and 0.1 mL at the follow-up visits.<br>• To change the requirements for infant formula. Infant formula meeting the European Society for Paediatric Gastroenterology, Hepatology and Nutrition guidelines, and the original protocol requirements are not available in all countries. Therefore the lower limit for carbohydrates has been changed from 10.5 to 9.5 g/100 kcal, and the medium-chain triglycerides content changed from <40% to ≤40%. The energy intake level has been deleted as this is not a specification for the formula content but a result of the feeding volume.<br>• To remove the prohibited concomitant medication criterion. The intention of the original protocol was to exclude patients with prior or current use of

Version	Date	Reason for Amendment or Administrative Changes
		corticosteroids at screening. It was not intended to prohibit their use once a patient had been included in the study.<br>• To provide details regarding the central laboratories in the study.<br>• To remove the requirement for study specific analysis of pasteurized breast milk composition. This requirement has been removed from the protocol because several problems with the analysis of samples at a central laboratory have been identified. The collection of pasteurized breast milk composition data was intended to give a rough idea of the pasteurized breast milk composition in the study (samples were only collected once per week for each patient). Therefore this data will only be collected where it is available.<br>• To add sodium to the routine laboratory assessment list of tests of special interest.<br>• In addition minor typographical errors and inconsistency between the synopsis and main protocol were corrected.
Version 3.0 Administrative Change 1.0	14 May 2012	• To update administrative information with new project manager details.<br>• To clarify that the Week 4 time point is the end of treatment (Day 29) and Baseline is Day 1.<br>• To clarify that in the 48 hours preceding randomization the use of systemic anti-infectives is exclusionary, with the exception of prophylactic use as per local clinical practice.<br>• To remove the requirement to collect retrospective body weight measurements (with the exception of birth weight and minimum recorded weight).<br>• To clarify that use of study specific equipment for body weight, total body length, and head circumference measurements after the end of the treatment period is preferred but not mandatory.<br>• To clarify that preplanned/administrative hospitalizations are not considered serious adverse events.<br>• To clarify the collection period for AEs and SAEs.<br>• To clarify that AEs of special interest will be handled as all other AEs and SAEs, but will be reported separately in the clinical study report.<br>• To change collection of intensity of AEs to only recording the maximum intensity, rather than changes in intensity.<br>• To allow qualified study personnel other than the primary investigator to perform the Bayley Scale of Infant and Toddler Development.<br>• To remove body composition assessments at Day 15, at 3 months, and the 12-months-corrected-age visit and add assessments at 40 weeks postmenstrual age and 3 months corrected age. The schedule of events was also amended to add these 2 extra visits.<br>• To update study drug packaging language to clarify how the syringe and tip cap are provided.<br>• To update study drug storage text in line with information in the investigator's brochure.<br>• To generalize text regarding study drug preparation to allow for sites that may be mixing in areas other than the pharmacy and neonatal intensive care unit.<br>• To clarify that recorded details of parenterally administered nutrition

Version	Date	Reason for Amendment or Administrative Changes
		containing a fat emulsion include volume and product name.<br>• To clarify that the time to sustained weight gain is one of the criteria used to evaluate the time to readiness for discharge endpoint.<br>• To remove information about posting results on www.ClinicalStudyResults.org, as the site no longer exists.<br>• To correct any minor typographical errors.
Version 4.0 Amendment 2.0	13 Dec 2012	• To determine the long-term safety and effect of rhBSSL, the follow-up period has been prolonged to 24 months corrected age from previously 12 months corrected age. At 12 months corrected age, the Bayley-III scale will be administered to evaluate delayed performance. Due to its low predictive validity to later IQ, except for the very low scores achieved by the most compromised infants, the Bayley-III scale will also be administered at 24 months corrected age, which is the age when a valid developmental examination can be performed (Vohr et al 2003). At 24 months corrected age, 2 new domains of the Bayley-III scale have been added together with the Child Behavior Checklist (CBCL) and the neurodevelopment disability composite.<br>• To assess the effect of rhBSSL on selected health economic variables, information regarding health care utilization, indirect resource use and chronic medical conditions/diagnosis will also be collected.<br>• To harmonize the global development program of rhBSSL, growth restriction has been added as a secondary efficacy endpoint.<br>• Administrative reasons.
Version 5.0 Administrative Change 2.0	10 June 2013	• To reschedule analysis of data<br>• To clarify data collection regarding outpatient visits<br>• To clarify that demographic data on multiple birth will be collected<br>• To clarify that body weight, body height, and head circumference at 24 months will be analyzed using descriptive statistics<br>• To add another presentation of weight and length data<br>• To add another presentation of feeding data<br>• To clarify how antibodies will be presented<br>• To clarify the statistical analysis of time to discharge, and further change the statistical analysis of time to readiness for discharge to be consistent with the analysis of time to discharge<br>• To change the sponsor’s medical monitor<br>• Administrative reasons

Protocol Synopsis

Protocol Number: BVT.BSSL-030

Title: A Prospective, Randomized, Double-Blind, Phase 3 Study Comparing rhBSSL and Placebo Added to Infant Formula or Pasteurized Breast Milk During 4 Weeks of Treatment in Preterm Infants Born Before Week 32 of Gestational Age

Study Phase: Phase 3

Objectives: The primary objective of this study is to demonstrate that recombinant human bile-salt-stimulated lipase (rhBSSL) improves growth in preterm infants as compared with placebo when administered in infant formula or pasteurized breast milk (PBM).

The secondary objectives of this study are

- To determine the effect of rhBSSL treatment in decreasing the risk of growth restriction
- To determine the effect of rhBSSL treatment in shortening the time of hospital stay
- To determine the effect of rhBSSL treatment in improving early development
- To determine the effect of rhBSSL treatment in decreasing readmittance to hospital
- To determine the effect of rhBSSL treatment in increasing the levels of docosahexaenoic acid (DHA) and arachidonic acid (AA)
- To compare the safety and tolerability of rhBSSL treatment with that of placebo treatment
- To determine the effect of rhBSSL treatment on neurodevelopment
- To determine the long-term effect of rhBSSL treatment on anthropometrics
- To determine the long-term safety of rhBSSL treatment
- To assess the effect of rhBSSL treatment on health care utilization
- To assess the effect of rhBSSL treatment on indirect resource use
- To assess the effect of rhBSSL treatment on chronic medical conditions/diagnoses

Exploratory objectives of this study are

- To assess the effect of rhBSSL treatment on fatty acid levels
- To assess the effect of rhBSSL on the body composition
- To assess the effect of rhBSSL treatment on the fecal calprotectin and microbiota contents

Patient Population: Approximately 432 patients will be enrolled at approximately 70 study centers in Europe to ensure at least 410 evaluable patients. Patients will be randomly assigned to study drug only if they meet all of the inclusion criteria and none of the exclusion criteria. The randomization will be stratified by feeding regimen (PBM or infant formula) and by size

Protocol Number: BVT.BSSL-030

for gestational age category (small for gestational age [SGA] or appropriate for gestational age [AGA]). At least 10% of the infants will be SGA at birth and at least 15% of the infants will be fed with PBM. An interim analysis to re-estimate the variance will be performed after 200 patients have completed the 4-week treatment period. The number of patients may be increased to a maximum of 534 evaluable patients (corresponding to 564 randomized patients) in case of a higher than anticipated variability.

Inclusion criteria: Each patient must meet all of the following criteria to be enrolled in this study:

1. Preterm infant born before Week 32 of gestation.
2. Preterm infant who is <33 weeks postmenstrual age at the time of randomization.
3. Preterm infant who is AGA or SGA at birth.
4. Preterm infant who is receiving food enterally (bottle or gavage tube) at a level of at least 100 mL/kg/day at randomization.
5. Preterm infant whose enteral feeding consists of only infant formula or only PBM at the time of inclusion, and who are expected to remain on only infant formula for 4 weeks, or only PBM for at least 2 weeks following treatment initiation.
6. Preterm infant who is expected not to receive any fresh breast milk for 4 weeks following treatment initiation.
7. Informed consent is obtained from the patient's legally acceptable representative. Every effort will be made to ensure informed consent is obtained from both parents. Where this is not possible, the local regulations will be followed in the definition of legally acceptable representative.

Exclusion criteria: Patients meeting any of the following criteria are not eligible for enrollment:

1. Expected stay at the hospital is less than 4 weeks from the first dose of study drug.
2. Currently receiving mechanical ventilation via endotracheal tube (continuous positive airway pressure [CPAP] or high-flow nasal cannula are not criteria for exclusion).
3. Require ≥30% oxygen (if on CPAP or in head box) or >0.5 L/min oxygen.
4. Evidence of severe brain disease or damage including grade III or IV peri- or intraventricular hemorrhage, meningitis or hydrocephalus, grade III or IV intracranial hemorrhage, or periventricular leukomalacia.
5. Presence of major dysmorphology or congenital abnormalities that are likely to affect growth and/or development.
6. Current clinical evidence of hemodynamically significant persistent ductus arteriosus.
7. Clinical evidence of sepsis (including low or high white blood cell count and/or low platelet count and bacteriologically proven evidence of systemic infection). This should be based on the investigator's opinion and available local laboratory reference

Protocol Number: BVT.BSSL-030

ranges.

8. Systemic anti-infective treatment within 48 hours prior to randomization, other than prophylactic treatment (eg, antifungal prophylaxis with fluconazole) as per local clinical practice.
9. Evidence of congenital infection (eg, cytomegalovirus).
10. Previous or current diagnosis of necrotizing enterocolitis (Bell's stage 2 or greater).
11. Prior or current treatment with corticosteroids, except hydrocortisone.
12. Presence of any condition that in the opinion of the investigator makes the patient unsuitable for inclusion.
13. Enrolled in another concurrent clinical intervention study.

Study Design: This is a randomized, double-blind, multicenter, prospective Phase 3 study designed to compare the efficacy and safety of rhBSSL with placebo during 4 weeks of treatment. When patients reach a level of enteral feeding of at least 100 mL/kg/day, they will be randomly assigned to receive rhBSSL or placebo added to the food. The randomization will be stratified by feeding regimen (PBM or infant formula) and by size for gestational age category (SGA or AGA). The feeding volumes will be decided according to each unit's feeding plan, and the patient's body weight.

The addition of study drug to the food will start on Day 1, which will be as soon as possible after randomization (either on the day of randomization or the day after). The predose assessments on Day 1 (the day the first dose of study drug is administered) will be the patient's baseline values. The administration of study drug will continue for 4 weeks. Follow-up visits will occur at 3 months after the first dose of study drug and at 12 and 24 months corrected age. There will be an additional follow-up visit at 6 months after the first dose of study drug if rhBSSL antibodies are found at 3 months. Patients who have body composition assessments during the treatment period will also return for follow-up assessments at 40 weeks postmenstrual age and 3 months corrected age. Telephone contacts will take place at 15, 18 and 21 months corrected age.

The primary efficacy measurement (growth velocity) will be made by frequent (at least 3 times per week) measurements of the infants' weight during treatment.

Endpoints: The primary efficacy variable is growth velocity in grams per kilogram per day during 4 weeks of treatment.

The study has the following secondary efficacy endpoints:

- Change from Baseline in body weight (g) at 3 months
- Body weight (g) at 12 and 24 months corrected age
- Change from Baseline in total body length (mm) at 4 weeks and 3 months
- Body length (mm) at 12 months corrected age

Protocol Number: BVT.BSSL-030

- Body height (cm) at 24 months corrected age
- Growth restriction, defined as growth velocity <15 g per kilogram bodyweight per day during 4 weeks of treatment
- Time to readiness for discharge
- Time to discharge
- Change from Baseline in head circumference (mm) at 4 weeks and 3 months
- Head circumference (mm) at 12 and 24 months corrected age
- Time from Baseline to 150 mL/kg/day of enteral feeding
- Readmission to hospital within 1 month of discharge
- Bayley Scale of Infant and Toddler Development III (Bayley-III) scores for the cognitive, language, motor, social-emotional and adaptive behavior domains at 24 months corrected age
- Neurodevelopment disability composite at 24 months corrected age
- Child Behavior Checklist (CBCL) scores at 24 months corrected age

The study has the following secondary safety endpoints:

- Adverse events
- Bayley-III scores for the cognitive, language and motor domains at 12 months corrected age
- Vomiting (frequency and volume)
- Vital signs (heart rate, blood pressure, body temperature)
- Laboratory variables amylase, bilirubin, aminotransferases, sodium, and urea
- Levels of vitamins A and D
- Presence of rhBSSL antibodies

The study has the following health economic endpoints:

- Number of outpatient visits from the time of initial hospital discharge to home up to 24 months corrected age
- Number of inpatient visits, days of hospitalization and days in intensive care unit from the time of initial hospital discharge to home up to 24 months corrected age
- Number of days lost from work related to the child´s condition from the time of initial hospital discharge to home up to 24 months corrected age
- Presence of chronic medical conditions/diagnoses at 24 months corrected age

Protocol Number: BVT.BSSL-030

The study has the following exploratory endpoints:

- Levels of fatty acids
- Percent body fat
- Fecal calprotectin concentration
- Fecal microbiota

Study Drug, Dosage, and Route of Administration: Recombinant human BSSL will be delivered as a sterile powder for oral solution, in a single-dose glass vial containing 8700 U (corresponding to 15 mg active rhBSSL). The content of 1 vial is intended for reconstitution in 1 mL of sterile water before addition to 100 mL of food. Each vial includes an overfill to ensure that 8700 U can be transferred to 100 mL of food. The matching placebo will also be delivered as a sterile powder for oral solution in an identical single-dose container.

Sample Size: Assuming a true difference in growth velocity between rhBSSL and placebo of 2.25 g/kg/day at 4 weeks and a standard deviation of 7 g/kg/day, 205 evaluable patients in each treatment arm provide 90% power to demonstrate that rhBSSL improves growth velocity compared with placebo (using a 2-sided test with a significance level of 5%). Allowing for 5% of patients to be excluded from the full analysis set, at least 432 patients will be randomized. These assumptions are based on the meta-analysis of Swedish Orphan Biovitrum’s 2 previous Phase 2 studies; the estimate of standard deviation has been increased by 30% to allow for greater variability within the Phase 3 study population. The appropriateness of the assumptions of a standard deviation of 7 g/kg/day will be examined at a planned blinded interim analysis (IA) which will be conducted once 200 patients have been randomized and followed up to 4 weeks or discontinuation (whichever occurs first). Swedish Orphan Biovitrum (Sobi) will use the IA results to decide whether the sample size of the study should be increased. If the estimated standard deviation is higher than the anticipated 7.0 g/kg/day, the sample size will be increased correspondingly up to a maximum of 534 evaluable patients (corresponding to 564 randomized patients). The sample size will not be decreased. As this is a blind re-estimation of variance, there is no impact on Type I error rate.

Protocol Number: BVT.BSSL-030

Statistical Methods: The null and alternative hypotheses are defined for the primary efficacy variable (weight gain measured as growth velocity during 4 weeks of treatment):

H_0: $\mu_{\text{rhBSSL}} = \mu_{\text{placebo}}$

H_A: $\mu_{\text{rhBSSL}} \neq \mu_{\text{placebo}}$

In order to calculate growth velocity, the natural log-transformed values of the baseline and all postbaseline weight assessments for each patient will be calculated. A linear regression model will then be fitted for each patient with a response variable of log(weight) and a predictor variable of time. Growth velocity for each patient will be estimated as the slope arising from the regression model, and will need to be multiplied by 1000 for conversion into the desired unit of grams per kilogram per day. The weight over time for each treatment group will be illustrated graphically and if the log-transformed data clearly deviate from the linearity assumption, a sensitivity analysis will be performed using untransformed data.

Statistical analysis for the primary efficacy variable will be performed using an analysis of covariance model including factors for treatment, feeding regimen (PBM or infant formula), size for gestational age category (SGA or AGA), with baseline weight included as a covariate. For the comparison between treatment groups, the point estimate and the associated 95% confidence interval and *P* value from the model will be presented.

The assumptions of normality and homogeneity of variance will be assessed by inspection of normal probability plots and residual plots. If either assumption is clearly not met, a corresponding nonparametric analysis will be performed (using the same analysis of covariance model, but using ranked response and covariate) to check the robustness of the results.

- Change from Baseline in body weight (g) at 3 months,
- Body weight (g) at 12 and 24 months corrected age,
- Change from Baseline in total body length (mm) at 4 weeks and 3 months,
- Body length (mm) at 12 months corrected age,
- Body height (cm) at 24 months corrected age,
- Change from Baseline in head circumference (mm) at 4 weeks and 3 months,
- Head circumference (mm) at 12 and 24 months corrected age

will be analyzed using an analysis of covariance approach similar to that used for the primary endpoint. No logarithmic transformation will be performed for these endpoints. Time to readiness for discharge and time to discharge will be analyzed using an analysis of variance model.

Readmission to hospital within 1 month of discharge and growth restriction will be analyzed using a logistic regression model with treatment, feeding regimen (PBM or infant formula), and size for gestational age category (SGA/AGA) as explanatory variables.

Protocol Number: BVT.BSSL-030

The Bayley-III cognition, language, motor, social-emotional and adaptive behavior composite scores at 24 months will be analyzed using an analysis of covariance model including factors for treatment, feeding regimen (PBM or infant formula), size for gestational age category (SGA or AGA), and critical confounders for neurodevelopment included as covariates.

The neurodevelopment disability composite scores will be analyzed using a logistic regression model with treatment, feeding regimen (PBM or infant formula), size for gestational age category (SGA or AGA) and critical confounders for neurodevelopment included as explanatory variables.

The remaining endpoints, including secondary safety, health economic and exploratory endpoints, will be analyzed using descriptive statistics.

The study results will be reported after 200 patients have completed the 12-months-corrected-age visit, including the Bayley assessment. A separate addendum to the report will be prepared when the remaining patients have completed the 12-month visit. The results of the 24-months-corrected-age assessments, as well as the health economic assessments and any serious adverse drug reactions, will be reported separately.

Date of Protocol: 10 June 2013

List of Abbreviations

Abbreviation	Definition
AA	arachidonic acid
ADR	adverse drug reaction
AE	adverse event
AGA	appropriate for gestational age
Bayley-III	Bayley Scales of Infant and Toddler Development, third edition
BSSL	bile-salt-stimulated lipase
CBCL	Child Behavior Checklist
CHMP	Committee for Medicinal Products for Human Use
CPAP	continuous positive airway pressure
DHA	docosahexaenoic acid
DSMB	data safety monitoring board
eCRF	electronic case report form
ECG	electrocardiogram
FAS	full analysis set
IA	interim analysis
ICF	informed consent form
ICH	International Conference on Harmonisation
IEC	independent ethics committee
IVRS	interactive voice recognition system
MedDRA	Medical Dictionary for Regulatory Activities
NICU	neonatal intensive care unit
PBM	pasteurized breast milk
PDCO	Pediatric Committee
PPS	per-protocol set
PVG	pharmacovigilance
rhBSSL	recombinant human bile-salt-stimulated lipase
SAE	serious adverse event
SGA	small for gestational age
Sobi	Swedish Orphan Biovitrum AB (publ)
TG	triglyceride

Definitions of Terms

Gestational age	In this study, gestational age should be determined based on the best obstetric measurement. Gestational age is expressed as completed weeks (eg, a 25-week 5-day fetus is considered a 25-week fetus (Engle 2004).
Postmenstrual age	The gestational age at birth plus the time elapsed since birth (Engle 2004).
Corrected age	Represents the age of the infant calculated from the expected date of birth (term) (Engle 2004).

1 Introduction

1.1 Premature Infants and Growth

The goal for nutritional support of preterm infants is to meet the intrauterine growth and nutrient retention in the neonate. Although awareness and understanding of the importance of optimized nutrition exists, there is room for considerable improvement in the nutritional regimens of preterm newborn infants. Growth in preterm infants, in terms of both weight gain and longitudinal growth, is often inadequate despite efforts to optimize parenteral nutrition and enteral feeding (Bloom et al 2003; Dusick et al 2003). In a growth observational study in extremely low-birth-weight infants, it was found that, at discharge from the neonatal intensive care unit (NICU) at 36 weeks postmenstrual age, most were below the 10th percentile weight for completed weeks of gestation (Ehrenkranz et al 1999). It has also been concluded that growth velocity during NICU hospitalization exerts a significant effect on growth and neurodevelopment at 18 to 22 months corrected age (Ehrenkranz et al 2006). In an observational study in a NICU, it was found that preterm infants could rarely meet the recommended dietary intakes and accrued a nutrient deficit that could not be regained before hospital discharge (Embleton et al 2001).

A short period of early dietary manipulation in preterm infants may have major beneficial consequences for later development, which suggests that optimal nutrition in different periods in early life, both prenatal and postnatal, may be critical. In general, the low–birth-weight preterm infants, whether appropriate for gestational age (AGA) or small for gestational age (SGA), who show catch-up growth toward normal in the first year of life are more likely to have more optimal outcomes in health, growth, and developmental status through childhood than children who do not catch up (Casey 2008).

1.2 Pancreatic Function in Early Infancy

In early infancy, and especially in the preterm infants, pancreatic exocrine functions are not fully developed (Manson and Weaver 1997). Several studies have shown low or negligible lipase activity in duodenal secretions of preterm infants (Zoppi et al 1972; Fredrikzon et al 1978; Lebenthal and Lee 1980). The lipase activity is then significantly and successively increased up to the age of 2 years (or more) (Zoppi et al 1972; Fredrikzon et al 1978; Lebenthal et al 1980).

Several different lipases act together in the lipolysis of dietary lipids. The most important ones are gastric lipase, colipase-dependent lipase (also called pancreatic lipase or pancreatic triglyceride [TG] lipase), pancreatic lipase-related protein 2, phospholipase A2, and bile-salt-stimulated lipase (BSSL). With the exception of gastric lipase, these enzymes are secreted by the exocrine pancreas. In adults, gastric lipase and colipase-dependent lipase are the 2 most important lipases involved in TG digestion and collectively hydrolyze each TG to 1 sn-2 monoglyceride and 2 free fatty acids (Armand 2007; Carey and Hernell 1992; Hamosh 2005). In the preterm pancreas, expression of pancreatic lipases is low compared with the adult pancreas (Lombardo 2001). In particular, colipase-dependent lipase has been shown to be poorly expressed and the levels are low in postprandial duodenal contents in preterm infants (Fredrikzon et al 1978). This is compensated for by expression of BSSL in the lactating mammary gland and secretion of the enzyme with the milk.

1.3 Bile-salt-stimulated Lipase

Bile–salt-stimulated lipase accounts for about 1% of the total protein in human breast milk and is present at concentrations from 0.1 to 0.2 g/L (Stromqvist et al 1997). The levels of BSSL are similar throughout the day (Freed et al 1986) and BSSL production is maintained for at least 3 months (Hernell et al 1977), although levels of BSSL may decline with duration of lactation (Torres et al 2001). Triglycerides comprise about 98% or more of all lipids in human breast milk or infant formula and account for about 50% of the energy content. Therefore, the lipases that hydrolyze the TGs, making the resulting hydrolysis products available for absorption, are of great importance for efficient energy utilization for the rapidly growing newborn infant. However, BSSL is inactivated during pasteurization of human breast milk and is not present in any infant formulas that exist for the nutrition of pre- or full-term neonates. It has been shown that fat absorption, weight gain, and linear growth is higher in infants fed fresh compared with pasteurized breast milk (PBM) (Andersson et al 2007; Williamson et al 1978).

Bile-salt-stimulated lipase has a broader substrate specificity than most lipases. Not only is the enzyme capable of completely hydrolyzing TGs but also vitamin A and cholesteryl esters. Thus, BSSL drives the intraluminal lipolysis toward completion and results in the formation of glycerol and free fatty acids, including long-chain polyunsaturated fatty acids, the latter being indispensable building blocks for the developing central nervous system.

1.4 Recombinant Human BSSL

Swedish Orphan Biovitrum AB (publ) (Sobi) is developing a recombinant human BSSL (rhBSSL) for addition to preterm infant formula or PBM (food). The rhBSSL is manufactured in a mammalian cell culture process with a Chinese hamster ovary cell line expressing human BSSL. The rhBSSL highly resembles its native counterpart and is orally administered.

Recombinant human BSSL is available as a lyophilized sterile powder for oral solution. After reconstitution, rhBSSL 8700 U is added to 100 mL infant formula or PBM (final concentration equal to 0.15 g/L), representing the physiological concentration found in breast milk. As the compound is most likely not absorbed when administered orally, distribution will remain localized to the gastrointestinal tract. It is expected that the compound will be deactivated by proteases to smaller peptides and amino acids. Any intact BSSL is expected to be excreted unchanged in the feces. Amino acids resulting from the enzymatic degradation of BSSL would be absorbed and handled as other dietary amino acids.

1.5 Completed Clinical Studies

Two Phase 2 studies in 63 preterm infants (BVT.BSSL-020 and BVT.BSSL-021) have been completed. These are the first clinical trials with rhBSSL in this patient population. To date, rhBSSL was well tolerated with a safety profile similar to that of placebo. Two serious adverse events (SAEs), one in each study, were reported. Both occurred during the placebo treatment period.

In these double-blind crossover studies, patients fed with infant formula (BVT.BSSL-020) or PBM (BVT.BSSL-021) were randomly assigned to have rhBSSL 0.15 g/L or placebo added to their food for the first 7 days. After a washout period of 2 days, the patients crossed over to the other treatment regimen and received an additional 7 days of treatment. At enrollment, the feeding volume was within the range of 150 to 180 mL/kg/day. The volume was kept constant for each individual throughout the study. The primary efficacy assessment (coefficient of fat absorption) was made by measuring the fat in the food and corresponding stool during the last 3 days (72 hours) of each treatment period.

A prospectively defined meta-analysis of the 2 studies showed that growth velocity was statistically significantly greater with rhBSSL than with placebo ($P<0.001$) and increased by

2.93 g/kg/day more on rhBSSL compared with placebo. This is considered clinically relevant and corresponds to a 20% improvement. Analysis of coefficient of fat absorption showed no statistically significant improvement with rhBSSL compared with placebo (*P*=0.069). There was no difference between rhBSSL and placebo in change in knee-to-heel length.

An exploratory analysis of the coefficient of absorption for arachidonic acid (AA) and docosahexaenoic acid (DHA) showed increased absorption of both these fatty acids with rhBSSL compared with placebo.

1.6 Study Rationale

Recombinant human BSSL is currently being developed by Sobi as a PBM or infant formula supplement to promote growth in preterm infants. This Phase 3 study is designed to confirm the findings of the Phase 2 studies, which demonstrated that rhBSSL improved growth in preterm infants. Specifically, this prospective Phase 3 randomized, double-blind study is designed to determine the efficacy (improved growth) of rhBSSL and to compare the safety and tolerability of rhBSSL treatment with that of placebo treatment after oral administration in the food of preterm infants.

The study design is in agreement with discussions held with the Committee for Medicinal Products for Human Use (CHMP) at a Scientific Advice meeting in July 2009, and with the Pediatric Investigational Plan agreed with the Pediatric Committee (PDCO) on 14 January 2011.

In addition, the patients will be followed up to 24 months corrected age to determine the long-term safety and effect (neurodevelopment and anthropometrics) of rhBSSL as well as assess the effect of rhBSSL on selected health economic variables.

1.7 Potential Risks and Benefits

As mentioned above, rhBSSL was well tolerated in the 2 studies performed in preterm infants. Recombinant human BSSL will be added to infant formula or PBM at a concentration within the range of what is found for native BSSL in mother’s milk as in the previously performed studies. Exposure to intact rhBSSL is expected to be limited to the gastrointestinal tract, where it will be degraded to smaller peptides and amino acids.

No clinical studies have been performed to determine antibody formation. However, the risk for an anti-product antibody response in preterm infants is considered to be low, as described in the investigator's brochure. In this study, serum levels of possible rhBSSL antibodies will be determined at Baseline, at the end of treatment (Day 29), and 3 months after the start of treatment and followed further if antibodies are detected.

Based on current experience, oral administration of rhBSSL 8700 U added to 100 mL of infant formula or PBM is not expected to present any additional risks to this population of preterm infants. However, the conduct of the study in NICUs allows for the continuous monitoring of patient safety and the accessibility to any necessary therapeutic measures.

Infants treated with rhBSSL may benefit from improved growth and improved uptake of long-chain polyunsaturated fatty acids such as DHA and AA, both important building blocks for the developing central nervous system.

2 Study Objectives and Endpoints

2.1 Study Objectives

2.1.1 Primary Objective

The primary objective of this study is to demonstrate that rhBSSL improves growth in preterm infants as compared with placebo when administered in infant formula or PBM.

2.1.2 Secondary Objectives

The secondary objectives of this study are

- To determine the effect of rhBSSL treatment in decreasing risk of growth restriction
- To determine the effect of rhBSSL treatment in shortening the time of hospital stay
- To determine the effect of rhBSSL treatment in improving early development
- To determine the effect of rhBSSL treatment in decreasing readmittance to hospital
- To determine the effect of rhBSSL treatment in increasing the levels of DHA and AA
- To compare the safety and tolerability of rhBSSL treatment with that of placebo treatment

- To determine the effect of rhBSSL treatment on neurodevelopment
- To determine the long-term effect of rhBSSL treatment on anthropometrics
- To determine the long-term safety of rhBSSL treatment
- To assess the effect of rhBSSL treatment on health care utilization
- To assess the effect of rhBSSL on indirect resource use
- To assess the effect of rhBSSL on chronic medical conditions/diagnoses

2.2 Exploratory Objectives

Exploratory objectives of this study are

- To assess the effect of rhBSSL treatment on fatty acid levels
- To assess the effect of rhBSSL on the body composition
- To assess the effect of rhBSSL treatment on the fecal calprotectin and microbiota contents

2.3 Study Endpoints

2.3.1 Primary Endpoint

The primary efficacy variable is growth velocity in grams per kilogram per day during 4 weeks of treatment.

2.3.2 Secondary Endpoints

The study has the following secondary efficacy endpoints:

- Change from Baseline in body weight (g) at 3 months
- Body weight (g) at 12 and 24 months corrected age
- Change from Baseline in total body length (mm) at 4 weeks and 3 months
- Body length (mm) at 12 months corrected age
- Body height (cm) at 24 months corrected age

- Growth restriction, defined as growth velocity <15 g per kilogram bodyweight per day during 4 weeks of treatment
- Time to readiness for discharge
- Time to discharge
- Change from Baseline in head circumference (mm) at 4 weeks and 3 months
- Head circumference (mm) at 12 and 24 months corrected age
- Time from Baseline to 150 mL/kg/day of enteral feeding
- Readmission to hospital within 1 month of discharge
- Levels of DHA and AA at 4 weeks
- Bayley Scale of Infant and Toddler Development, third edition (Bayley-III) scores for the cognitive, language, motor, social-emotional and adaptive behavior domains at 24 months corrected age
- Neurodevelopment disability composite at 24 months corrected age
- Child Behavior Checklist (CBCL) scores at 24 months corrected age

Section 3.7.2 contains additional details for the secondary efficacy endpoints.

The study has the following secondary safety endpoints:

- Adverse events (AEs)
- Bayley-III scores for the cognitive, language and motor domain at 12 months corrected age
- Vomiting (frequency and volume)
- Vital signs (heart rate, blood pressure, body temperature)
- Laboratory variables amylase, bilirubin, aminotransferases, sodium, and urea
- Levels of vitamins A and D
- Presence of rhBSSL antibodies

The study has the following health economic endpoints:

- Number of outpatient visits from the time of initial hospital discharge to home up to 24 months corrected age
- Number of inpatient visits, days of hospitalization and days in intensive care unit from the time of initial hospital discharge to home up to 24 months corrected age
- Number of days lost from work related to the child´s condition from the time of initial hospital discharge to home up to 24 months corrected age
- Presence of chronic medical conditions/diagnoses at 24 months corrected age

2.3.3 Exploratory Endpoints

The study has the following exploratory endpoints:

- Levels of fatty acids
- Percent body fat
- Fecal calprotectin concentration
- Fecal microbiota

3 Investigational Plan

3.1 Study Design

This is a randomized, double-blind, multicenter, prospective Phase 3 study designed to compare the efficacy and safety of rhBSSL with placebo during 4 weeks of treatment. When patients reach a level of enteral feeding of at least 100 mL/kg/day, they will be randomly assigned to receive rhBSSL 8700 U or placebo added to 100 mL of the food. The randomization will be stratified by feeding regimen (PBM or infant formula) and by size for gestational age category (SGA or AGA). The feeding volumes will be decided according to each unit's feeding plan, and the patient's body weight (Section 3.6.5.1).

The addition of study drug to the food will start on Day 1, which will be as soon as possible after randomization (either on the day of randomization or the day after). The predose assessments on Day 1 (the day the first dose of study drug is administered) will be the

patient's baseline values. The administration of study drug will continue for 4 weeks. Follow-up visits will occur at 3 months after the first dose of study drug and at 12 and 24 months corrected age. There will be an additional follow-up visit at 6 months after the first dose of study drug if rhBSSL antibodies are found at 3 months. Patients who have body composition assessments during the treatment period will also return for follow-up assessments at 40 weeks postmenstrual age and 3 months corrected age. Telephone contacts will take place at 15, 18 and 21 months corrected age.

For patients that have conducted their 12-months-corrected-age visit before the implementation of amendment 2, i.e., before the long-term follow up was added, a separate visit will be conducted to collect informed consent, confounders for neurodevelopment, serious adverse drug reactions (ADRs) as well as perform the health economic assessments. This visit should take place as soon as the amendment has been approved by the regulatory authority and the independent ethics committee (IEC).

The primary efficacy measurement (growth velocity) will be made by frequent (at least 3 times per week) measurements of the infants' weight during treatment.

A schematic of the study design is presented in Figure 3–1.

Figure 3–1 Study Schematic

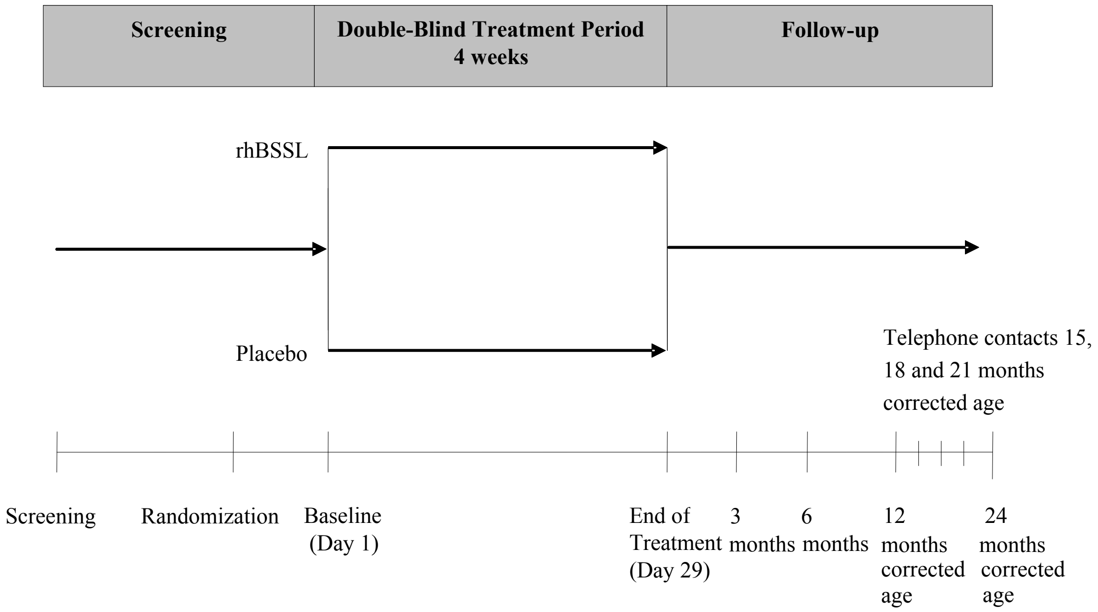

Note: Patients who have body composition assessments during the treatment period will also return for follow-up assessments at 40 weeks postmenstrual age and 3 months corrected age.
Abbreviation: rhBSSL, recombinant human bile-salt-stimulated lipase

3.2 Selection of Study Population

It is estimated that 432 patients will be randomized at approximately 70 study centers in Europe to ensure at least 410 evaluable patients. Patients will be randomly assigned to study drug only if they meet all of the inclusion criteria and none of the exclusion criteria. The randomization will be stratified by feeding regimen (PBM or infant formula) and by size for gestational age category (SGA or AGA). At least 10% of the infants will be SGA at birth and at least 15% of the infants will be fed with PBM. An interim analysis to re-estimate the variance will be performed after 200 patients have completed the 4-week treatment period. The number of patients may be increased to a maximum of 534 evaluable patients (corresponding to 564 randomized patients) in case of a higher than anticipated variability.

Since fresh human breast milk contains BSSL, any coadministration of fresh milk will confound the results of this study. Therefore, for strictly scientific and statistical reasons, the use of fresh breast milk must be restricted. On the other hand, for ethical reasons, no mother can be prohibited from providing fresh milk, either by direct breast feeding or administered via gavage. Thus the 2 aspects, the need to restrict the use of fresh breast milk and the ethical considerations, will have to be carefully balanced.

At the time of inclusion the enteral feeding should consist of only infant formula or only PBM and it should be expected that the patient will not receive any fresh breast milk during the 4 week treatment period. For restrictions regarding later introduction of breast feeding, during the planned treatment period, see Section 3.6.5.5.

3.2.1 Inclusion Criteria

Each patient must meet all of the following criteria to be enrolled in this study:

1. Preterm infant born before Week 32 of gestation.
2. Preterm infant who is <33 weeks postmenstrual age at the time of randomization.
3. Preterm infant who is AGA or SGA at birth.
4. Preterm infant who is receiving food enterally (bottle or gavage tube) at a level of at least 100 mL/kg/day at randomization.
5. Preterm infant whose enteral feeding consists of only infant formula or only PBM at the time of inclusion, and who are expected to remain on only infant formula for 4 weeks, or only PBM for at least 2 weeks following treatment initiation.
6. Preterm infant who is expected not to receive any fresh breast milk for 4 weeks following treatment initiation.
7. Informed consent is obtained from the patient’s legally acceptable representative. Every effort will be made to ensure informed consent is obtained from both parents. Where this is not possible, the local regulations will be followed in the definition of legally acceptable representative.

3.2.2 Exclusion Criteria

Patients meeting any of the following criteria are not eligible for enrollment:

1. Expected stay at the hospital is less than 4 weeks from the first dose of study drug.

2. Currently receiving mechanical ventilation via endotracheal tube (continuous positive airway pressure [CPAP] or high-flow nasal cannula are not criteria for exclusion).

3. Require ≥30% oxygen (if on CPAP or in head box) or >0.5 L/min oxygen.

4. Evidence of severe brain disease or damage, including grade III or IV peri- or intraventricular hemorrhage, meningitis or hydrocephalus, grade III or IV intracranial hemorrhage, or periventricular leukomalacia.

5. Presence of major dysmorphology or congenital abnormalities that are likely to affect growth and/or development.

6. Current clinical evidence of hemodynamically significant persistent ductus arteriosus.

7. Clinical evidence of sepsis (including low or high white blood cell count and/or low platelet count and bacteriologically proven evidence of systemic infection). This should be based on the investigator's opinion and available local laboratory reference ranges.

8. Systemic anti-infective treatment within 48 hours prior to randomization, other than prophylactic treatment (eg, antifungal prophylaxis with fluconazole) as per local clinical practice.

9. Evidence of congenital infection (eg, cytomegalovirus).

10. Previous or current diagnosis of necrotizing enterocolitis (Bell's stage 2 or greater).

11. Prior or current treatment with corticosteroids, except hydrocortisone.

12. Presence of any condition that in the opinion of the investigator makes the patient unsuitable for inclusion.

13. Enrolled in another concurrent clinical intervention study.

3.3 Withdrawal of Patients

3.3.1 Reasons for Withdrawal

The investigator may withdraw a patient from the study treatment for the following reasons:

1. Patient experiences serious or intolerable (as determined by the investigator) AEs; or

2. Patient's legally acceptable representative requests an early discontinuation for any reason.

The investigator may also withdraw a patient from study treatment based on his or her own judgment, upon the request of Sobi, or if Sobi terminates the study. Upon occurrence of a serious or intolerable AE, the principal investigator will confer with PPD. If a patient is discontinued because of an AE, the event will be followed up until it is resolved or the condition is stable.

3.3.2 Handling of Withdrawals

The patient's legally acceptable representative is free to request withdrawal of the patient from the study at any time. Patient participation in the study may be stopped at any time at the discretion of the investigator or at the request of Sobi.

When a patient is withdrawn, the reasons for withdrawal will be recorded by the investigator on the relevant page of the electronic case report form (eCRF). Whenever possible, all patients who are prematurely withdrawn from the study treatment will undergo the scheduled follow-up visits. Parents (or other legally acceptable representative) of a patient who fail to return with the patient for follow-up assessments will be contacted by the study center (2 documented phone calls followed by 1 registered letter) in an attempt to have them comply with the protocol.

It is vital to obtain follow-up data on any patient withdrawn because of an AE or SAE. In every case, efforts must be made to undertake protocol-specified safety and follow-up procedures.

3.3.3 Replacements

The number of patients randomly assigned to treatment will be adjusted should the actual percentage of patients to be excluded from the full analysis set (FAS) differ significantly from the assumed value of 5% to ensure that there are at least 410 evaluable patients included in the FAS. The required number of patients may be increased to a maximum of 534 evaluable patients (corresponding to 564 randomized patients) based on the variability observed in the interim analysis.

3.4 Study Procedures

Before performing any study procedures, the legally acceptable representative of potential patients will sign an informed consent form (ICF). The patient's legally acceptable representative will have the opportunity to receive answers to any questions they might have before signing. The investigator will also sign the ICF.

Informed consent will also be collected from the legally acceptable representative of the patients in the study for the 24-months-corrected-age assessments, as well as the health economic assessments and any serious ADRs. This consent will be collected when amendment 2 has been implemented and earliest at the 12-months-corrected-age visit. In addition, informed consent will be collected from the legally acceptable representatives for the collection of confounders for neurodevelopment.

Patients will undergo the procedures at the time points specified in the schedule of events as shown in Table 6–1 and Table 6–2.

3.4.1 Medical History

Medical history will be recorded in the eCRF at Screening.

3.4.2 Demography Assessments and Recording of Confounders of Neurodevelopment

3.4.2.1 Demography Assessments

At Baseline the following should be recorded:

- Body weight
- Total body length
- Head circumference
- Sex
- Race
- Actual birth date

- Expected birth date
- Body weight at birth
- Information related to multiple birth

3.4.2.2 Confounders of Neurodevelopment

Even with randomization, confounding of intervention groups may occur when assessing developmental outcomes. Numerous factors related to child developmental outcome have been identified as confounding variables which need to be considered in randomized, controlled, trials of infant nutritional interventions. These variables include socioeconomic status, parental education, race, income, maternal IQ, age, psychological status, parity, marital status, child gender, number of children in the home and exposure to teratogenic substances (Singer 2001). In this study, confounders deemed to have a possible influence on the neurodevelopmental outcome have been selected.

The following confounders of neurodevelopment will be recorded as soon as informed consent has been provided (earliest at the 12-months-corrected-age visit):

- Medical and social factors of the biological mother
    - maternal height, weight and age at time of conception
    - presence of diabetes and/or preeclampsia during this pregnancy
    - nicotine use, alcohol intake and/or drug abuse during this pregnancy
    - number of previous pregnancies (all pregnancies that lasted beyond week 16 of gestation to be included)

If the biological mother is not the legally authorized representative, the above factors will not be collected.

The following confounders of neurodevelopment will be recorded at the 24-months-corrected-age visit:

- Factors of the household the patient currently lives in
    - number of children ≤18 years of age in the household
    - number of parents/primary caregivers in the household

- socio economic status of parents/primary caregivers (educational level, employment status and income level)

If the parent/primary caregiver is not the legally authorized representative, the above factors will not be collected.

3.4.3 Efficacy Assessments

3.4.3.1 Body Weight

Body weight at birth and the lowest measured body weight should be retrospectively recorded in the eCRF. The patient's body weight in grams will, as a minimum, be measured at Baseline (Day 1, before start of study drug administration), on Days 8, 15, 22, and 29, and on at least 2 other time points per week. Thereafter, body weight should be recorded at least weekly until discharge, and at each scheduled follow-up visit (Table 6–1 and Table 6–2). Body weight measured at any other time point from randomization up to discharge should also be recorded in the eCRF, but not more than 1 record per day.

During the treatment period, the body weight should be measured according to standard unit procedures, as near to the same time each day as is possible, and using the same scale (provided for the study), with graduation of 2 g, each time. The weight of catheters, tubes, and other items that cannot be removed should be subtracted from the total measured weight. Preferably the same equipment should also be used for assessments made after the treatment period, where possible.

At the 24-months-corrected-age visit, the body weight does not need to be measured using the scale provided for the study. The body weight will be measured in accordance with hospital routine for children of this age.

3.4.3.2 Total Body Length

During the treatment period, the patient's total length (mm) will be measured from the crown to the heel using a preterm infant length board provided for the study and recorded in the eCRF. Preferably and where possible the same equipment should also be used for assessments made after the treatment period. Total body length should be measured according to separate detailed instructions. This measurement should be made at Baseline (Day 1), Days 8, 15, 22, and 29, at discharge, and at the 3-months and the 12-months-

corrected-age follow-up visits (Table 6–1 and Table 6–2). Measurements up to discharge should be taken as near to the same time each day as is possible.

3.4.3.3 Total Body Height

At the 24-months-corrected-age visit, the length measurement (lying) will be replaced by height measurement (standing). The body height will be measured in accordance with hospital routine for children of this age.

3.4.3.4 Head Circumference

During the treatment period, the circumference of the patient's head (mm) will be measured, using a nonstretch measuring tape provided for this study, and recorded in the eCRF. Preferably and where possible the same equipment should also be used for assessments made after the treatment period. The measuring of head circumference should be made according to separate detailed instructions. This measurement should be made at Baseline (Day 1), Days 8, 15, 22, and 29, at discharge, and at each scheduled follow-up visit (Table 6–1 and Table 6–2). Measurements up to discharge should be taken as near to the same time each day as is possible.

3.4.3.5 Time to Readiness for Discharge

The following should be recorded in the eCRF:

- The date when the infant is able to suckle feed (coordinate swallowing and breathing).
- The date when the patient is able to control the body temperature, ie, able to maintain normal body temperature while clothed in an open bed with normal ambient temperature.
- The date when the patient is able to maintain stable cardiorespiratory function.

3.4.3.6 Docosahexaenoic Acid (DHA) and Arachidonic Acid (AA)

Fatty acids, including DHA and AA, will be determined in either serum or plasma at Baseline (Day 1) and at the end of treatment (Day 29). Fatty acids other than DHA and AA are considered exploratory assessments.

Total fatty acids require a blood volume of 200 µL, while 500 µL of blood is required for a complete analysis in both phospholipid and triglyceride fractions. Section 3.4.7 provides further details of the sampling.

3.4.3.7 Bayley Scales of Infant and Toddler Development

A description of the Bayley-III assessment is provided in Section 3.4.4.6.

3.4.3.8 Child Behavior Checklist

The CBCL for 1.5 to 5 years of age is a parent-rated scale that will be used to evaluate mental health and behavioral development of children at 24 months corrected age. It consists of 99 problem items and one open-ended item for recording other problems not listed on the form. Each item will be rated by the parent/primary caregiver on a 0-2 point scale, where 0=not true, 1= somewhat or sometimes true and 2=very true or often true. Instructions on how to complete the CBCL will be given by a trained health care professional and the questionnaire should be completed at the site. For administrative reasons it is allowed to administer the CBCL on a different day than the main visit, as long as the administration takes place within the visit window specified in the schedule of events as shown in Table 6–2.

There are 7 syndrome subscales (emotionally reactive, anxious/depressed, somatic complaints, withdrawn, sleep problems, attention problems and aggressive behavior). These subscales are evaluated by summarizing the ratings for the items comprising each syndrome (Rescorla 2005). The subscales are also summarized as internalizing problems (emotionally reactive, anxious/depressed, somatic complaints and withdrawn items) and externalizing problems (attention problems and aggressive behavior items), and a total problem score is calculated.

The CBCL is part of the Achenbach System of Empirically Based Assessment (ASEBA) which has been used in several countries in research and clinical work (Rescorla 2005). The scale has good psychometric properties and confirmation of the reliability and validity of the problem scales have been confirmed for the Dutch version of the CBCL (Potijk et al 2012).

3.4.3.9 Neurodevelopment Disability Composite

The neurodevelopment disability composite will be assessed at 24 months corrected age and is defined as presence of any one of the following:

- A composite score of less than 85 on any of the cognitive, language or motor domains of Bayley-III
- Bilateral deafness, defined as need for bilateral amplification
- Bilateral blindness, defined as corrected visual acuity of less than 20/200 (or equivalent) in the better eye
- Cerebral palsy (CP), defined as hypotonia, spastic diplegia, hemiplegia or quadriplegia causing functional deficits that require rehabilitation services

Similar neurodevelopment disability composites have been widely used to study neurodevelopmental outcome (Schmidt et al 2007; Wadhawan et al 2009; Wickremasinghe et al 2012).

3.4.4 Safety Assessments

3.4.4.1 Adverse Events

3.4.4.1.1 Definitions

Adverse Event

An AE is defined as any untoward medical occurrence, including a clinically significant laboratory finding, symptom, or disease, in a patient enrolled into this study regardless of its causal relationship to study drug.

Treatment-Emergent Adverse Event

A treatment-emergent AE is defined as any event not present before exposure to study drug or any event already present that worsens in either intensity or frequency after exposure to study drug.

Serious Adverse Event

An SAE is defined as any event that

- results in death,
- is immediately life threatening (includes events that put patients at risk of death at the time of the event but not events that may have caused patient death if more severe),
- requires inpatient hospitalization or prolongation of existing hospitalization,
- results in persistent or significant disability or incapacity,
- is a congenital anomaly or birth defect.

Important medical events that may not result in death, be life threatening, or require hospitalization may be considered an SAE when, based upon appropriate medical judgment, they may jeopardize the patient and may require medical or surgical intervention to prevent one of the outcomes listed in this definition.

However, preplanned/administrative hospitalization will **not be considered** as an SAE, for example:

- Hospitalization for a procedure that is planned prior to signing the informed consent for enrolling in the study, without a worsening of a pre-existing disease. The procedure must be documented in the source documents and in the eCRF.
- Hospitalization or prolongation of hospitalization for administrative reasons such as technical, practical, or social reasons, in absence of an AE (eg, preplanned hospitalization for vaccinations according to a vaccination program or hospital general policies). This must also be documented in the source documents and in the eCRF.

Please note that hospitalization or prolonged hospitalization for a complication to the preplanned/administrative procedure remains a reportable SAE.

Serious Adverse Drug Reaction

A serious ADR is defined as any event that fulfills the criteria for an SAE and where a causal relationship between the event and the study drug is suspected by the investigator.

3.4.4.1.2 Eliciting and Documenting Adverse Events

The investigator is responsible for reporting all AEs that are observed or reported during the study, regardless of their relationship to study drug or their clinical significance.

The investigator will report and monitor AEs of special interest, ie, infections, necrotizing enterocolitis, sepsis, intraventricular hemorrhage, retinopathy of prematurity, hearing impairment, bronchopulmonary dysplasia, and cholestasis. These AEs will be handled as AEs, or if meeting the serious criteria, as SAEs. The AEs of special interest will be discussed in a separate section of the study report.

Adverse events: AEs will be recorded from start of treatment on Day 1 until the 3-month follow-up visit.

Adverse events present at the 3-month visit should be followed up at the patient's next visit (6-month [if applicable] or 12 months). Adverse events that have not resolved or stabilized at the 12-months-corrected-age visit should be followed up until satisfactory resolution or until the principal investigator or subinvestigator deems the event to be chronic or not clinically significant or the patient's condition to be stable, but without further recordings in the eCRF.

Serious AEs: SAEs will be reported from the time the informed consent has been signed up to the 12-months-corrected-age visit. The investigator will ask the patient's legally acceptable representative about any ongoing or previous (since last visit) events that fulfill the SAE criteria.

Any SAEs present at the 12-months-corrected-age visit should be followed up until satisfactory resolution or until the principal investigator or subinvestigator deems the event to be chronic or not clinically significant or the patient's condition to be stable, but without further recordings in the eCRF. Additional information received regarding patients with ongoing SAEs after 12 months should be reported via fax notification as described in Section 3.4.4.1.3.

Serious ADRs: Serious ADRs will be reported up to the 24-months-corrected-age visit. At the time of consent to the follow-up assessments (if not done at the 12-months-corrected-age visit), at telephone contacts and at the 24-months-corrected-age visit, the patient's legally acceptable representative will be asked about any ongoing or previous (since last contact or visit) events that fulfills the SAE criteria. The investigator will then judge if a causal relationship between the SAE and the study drug is suspected i.e., if the SAE is a serious ADR.

Any serious ADRs present at the 24-months-corrected-age visit should be followed up until satisfactory resolution or until the principal investigator or subinvestigator deems the event to be chronic or not clinically significant or the patient's condition to be stable, but without further recordings in the eCRF. Additional information received regarding patients with ongoing serious ADRs after 24 months should be reported via fax notification as described in Section 3.4.4.1.3.

In addition, AEs will be recorded on the eCRF from any study-specific data collected during the study (eg, laboratory values, physical examination findings, ECG changes) or any other documents or data that are relevant to patient safety.

3.4.4.1.3 Reporting Adverse Events

AEs reported or observed will be recorded on the AE page of the eCRF as described in the eCRF completion guidelines. Information collected will include date and time of onset, investigator-specified assessment of severity and relationship to study drug, date and time of resolution of the event, seriousness, as well as any required treatment or evaluations, and outcome. Adverse events resulting from concurrent illnesses, reactions to concurrent illnesses, reactions to concurrent medications, or progression of disease states must also be reported. All AEs will be followed to adequate resolution. The Medical Dictionary for Regulatory Activities (MedDRA) will be used to code all AEs (Section 4.10).

Any medical condition that is present at the time that the patient is screened but does not deteriorate should not be reported as an AE. However, if it deteriorates at any time during the study, it should be recorded as an AE.

A hospitalization for administrative reasons in the absence of an AE is not considered to be an SAE. If a patient is hospitalized for a procedure that was planned prior to when the

informed consent was obtained, it is not considered an SAE. For further information, see Section 3.4.4.1.1.

Up to the 12-months-corrected-age visit, the investigator's assessment of an AE's relationship to study drug is part of the documentation process, but it is not a factor in determining what is or is not reported in the study. If there is any doubt as to whether a clinical observation is an AE, the event will be reported. Any SAE reported after the 12- and up to the 24-months-corrected-age visits must be assessed by the investigator regarding relationship to study drug and must if considered related (i.e. is a serious ADR) be reported in the study.

The severity and the relationship or association of the study drug in causing or contributing to the AE will be characterized as defined in Sections 3.4.4.1.4 and 3.4.4.1.5.

Serious Adverse Events

Any AE considered serious by the investigator or subinvestigator or which meets SAE criteria (Section 3.4.4.1.1) occurring up to the 12-months-corrected-age visit, and any AE which meets SAE criteria (Section 3.4.4.1.1) and where a causal relationship between the event and the study drug is suspected by the investigator (serious ADR) occurring up to the 24-months-corrected-age visit, must be reported to PPD Pharmacovigilance (PVG) Department within 24 hours from the time study center personnel first learn about the event. The following contact information should be used for SAE and serious ADR reporting (Fax line for sending the SAE notification and the hotline and e-mail for questions):

Fax line for sending the SAE and serious ADR notification: PPD PVG Fax line: +44 1223 374102

The hotline and e-mail for questions:
PPD PVG Hotline: +44 1223 374240
PPD PVG E-mail: emeaasiasafetycentral.sm@ppdi.com

PPD PVG address:
Medical Affairs/Pharmacovigilance, PPD Granta Park, Great Abington, Cambridge CB21 6GQ, United Kingdom

The SAE and serious ADR must be reported within 24 hours by completing the eCRF pages and sending a paper SAE notification fax to PVG. If the patient is hospitalized during the

course of an SAE/serious ADR, or because of an SAE/ serious ADR, a copy of the hospital discharge summary should be faxed to PPD as soon as it becomes available. Withdrawal from the study and all therapeutic measures will be at the discretion of the principal investigator or subinvestigator. All SAEs (regardless of relationship with the study treatment) will be followed until satisfactory resolution or until the principal investigator or subinvestigator deems the event to be chronic or the patient to be stable.

PPD on behalf of Sobi is responsible for reporting relevant SAEs to the competent authority, other applicable regulatory authorities, and participating investigators, in accordance with European Clinical Trials Directive (Directive 2001/20/EC), International Conference on Harmonisation (ICH) guidelines, and/or local regulatory requirements.

For SAEs occurring up to the 12-months-corrected-age visit, PPD on behalf of Sobi is responsible for reporting unexpected fatal or life-threatening events associated with the use of the study drug (expedited reports) to the regulatory authorities, ethics committees, or principal investigators as required by fax, e-mail, or courier within 7 calendar days after being notified of the event. PPD on behalf of Sobi should report other relevant SAEs associated with the use of the study drug to the appropriate regulatory authorities (according to local guidelines), investigators, and central ethics committees by a written safety report within 15 calendar days of notification.

For serious ADRs occurring after the 12-months-corrected-age visit, Drug Safety at Sobi will be responsible for reporting to appropriate regulatory authorities and PPD will be responsible for reporting to central ethics committees and principal investigators within the same time frames as above.

3.4.4.1.4 Assessment of Severity

The severity, or intensity, of an AE refers to the extent to which an AE affects the patient's daily activities. The intensity of the AE will be rated as mild, moderate, or severe using the following criteria:

Mild: These events require minimal or no treatment and do not interfere with the patient's daily activities that are age appropriate.

Moderate: These events result in a low level of inconvenience or concern with the therapeutic measures. Moderate events may cause some interference with functioning.

Severe: These events interrupt a patient's usual age-appropriate daily activity and may require systemic drug therapy or other treatment. Severe events are usually incapacitating.

Only one intensity of the AE will be recorded. If the intensity of the AE changes, the maximum intensity of the event will be recorded.

3.4.4.1.5 Assessment of Causality

The relationship or association of the study drug in causing or contributing to the AE will be characterized using the following classification and criteria:

Not related: This relationship suggests that there is no association between the study drug and the reported event.

Unlikely: This relationship suggests that the temporal sequence of the clinical event, including a laboratory test abnormality, with drug administration makes a causal relationship improbable, and that other drugs, chemicals, or underlying diseases provide plausible explanations.

Possible: This relationship suggests that treatment with the study drug caused or contributed to the AE, ie, the event follows a reasonable temporal sequence from the time of drug administration or follows a known response pattern to the study drug, but could also have been produced by other factors.

Probable: This relationship suggests that a reasonable temporal sequence of the event with study drug administration exists and, based upon the known pharmacological action of the drug, known or previously reported adverse reactions to the drug or class of drugs, or judgment based on the investigator's clinical experience, the association of the event with the study drug seems likely.

Definite: This relationship suggests that a definite causal relationship exists between drug administration and the AE, and other conditions (concurrent illness, progression/expression of disease state, or concurrent medication reaction) do not appear to explain the event.

"Not related" and "unlikely" will be included in the category "unrelated" (ie, not having a reasonable suspected causal relationship to the study drug) in sponsor reports. "Possible", "probable" and "definite" will be included in the category "related" (ie, having a reasonable causal relationship to the study drug) in sponsor reports.

3.4.4.1.6 Follow-up of Patients with Reported Adverse Events

3.4.4.1.6.1 Adverse Events Occurring before the 12-Months-corrected-age Visit

All AEs (including SAEs) occurring up to the 12-months-corrected-age visit, captured in accordance with Section 3.4.4.1.2, must be followed to satisfactory resolution or until the principal investigator or subinvestigator deems the event to be chronic or not clinically significant or the patient's condition to be stable, but without further recordings into the eCRF after the 12-months-corrected-age visit. Follow-up information from SAEs that are ongoing at the 12-months-corrected-age visit will be reported via the fax notification as described in Section 3.4.4.1.3.

Sobi retains the right to request additional information for any patients with ongoing AE(s)/SAE(s) at the 12-months-corrected-age visit, if judged necessary.

3.4.4.1.6.2 Serious Adverse Drug Reactions Occurring after the 12-Months-corrected-age Visit

All serious ADRs occurring after the 12-months-corrected-age visit, captured in accordance with Section 3.4.4.1.2, must be followed to satisfactory resolution or until the principal investigator or subinvestigator deems the event to be chronic or not clinically significant or the patient's condition to be stable, but without further recordings into the eCRF after the 24-months-corrected-age visit. Follow-up information from SAEs that are ongoing at the 24-months-corrected-age visit will be reported via the fax notification as described in Section 3.4.4.1.3.

Sobi retains the right to request additional information for any patients with ongoing serious ADRs s at the 24-months-corrected-age visit, if judged necessary.

3.4.4.2 Physical Examination

Physical examination will be performed at Screening, Baseline, Days 8, 15, 22, and 29, at discharge, and at the follow-up visits at 3 months and 12 months corrected age. Any clinically significant changes from Baseline up to the 3-months visit noted during the physical examinations should be reported as AEs. See instructions regarding SAE/serious ADR reporting after this visit in Section 3.4.4.1.2.

3.4.4.3 Gastrointestinal Tolerability

Tolerability assessments will be performed at Baseline and then daily until the end of treatment. The tolerability assessments will consist of recording incidences and estimated volumes of vomiting. Vomiting should also be reported as an AE if considered clinically significant.

3.4.4.4 Vital Signs and Body Temperature

Blood pressure, heart rate, and body temperature will be recorded at Screening, Baseline, Days 8, 15, 22, and 29, and at discharge. Measurements will be performed according to standard routines at the study center.

A rectal body temperature above 38.0°C, or a corresponding temperature measured by other methods, should be reported as an AE (fever).

The reporting of abnormalities as both vital signs and AEs should be avoided. An asymptomatic vital sign value should only be reported as an AE if it is clinically significant, if it fulfills a criterion for an SAE, or if it causes withdrawal of the patient from the study. If an abnormal vital sign value is associated with clinical signs and symptoms, the sign/symptom should be reported as an AE and the associated vital sign should be considered additional information.

3.4.4.5 Electrocardiogram

Routine care electrocardiograms (ECGs) will be conducted according to the hospitals' standard procedure. No extra ECGs will be conducted. Results of any ECGs conducted as part of routine care will be recorded in the eCRF. Any ECG abnormalities considered by the investigator to be clinically significant should be reported as an AE.

3.4.4.6 Bayley Scales of Infant and Toddler Development

The Bayley-III is an individually administered instrument that assesses the developmental functioning of infants and young children between 1 month and 42 months of age, across five domains: cognitive, motor, language, social-emotional, and adaptive behavior. Assessments of the cognitive, motor and language domains are conducted using items administered to the child; assessment of the social-emotional and adaptive behavior domains are conducted using parent/primary caregiver response to a questionnaire. Bayley-III is primarily used to identify young children with developmental delay and to assist health care providers in the intervention planning. The Bayley Scales of Infant and Toddler Development, second edition, has been used extensively in research to track the effects of intervention on children's development. The psychometric properties of the Bayley Scales of Infant and Toddler Development, second edition, have been maintained in Bayley-III (Bayley et al 2006). In this study, Bayley-III will be used to assess neurodevelopment. The cognitive, motor, and language domains will be assessed at 12 and 24 months corrected age. At 24 months corrected age, the social-emotional and adaptive behavior domains will also be assessed.

The cognitive, motor, and language domains will be assessed by a trained health care professional. The questionnaires in the social-emotional and adaptive behavior domains will either be completed by the parents/primary caregivers or read to the parents/primary caregivers by a trained health care professional and the response from the parents/primary caregivers recorded. The social-emotional and adaptive behavior questionnaires may either be completed at the site or at home. Instructions on how to complete the questionnaires will be given by a trained health care professional. For administrative reasons it is allowed to administer and complete the Bayley-III on a different day than the main visit as long as the administration takes place within the visit window specified in the schedule of events as shown in Table 6–2.

A composite score will be calculated for each of the 5 domains, and scaled scores will be calculated for each subtest as well as for the cognitive and social-emotional domains.

At 24 months corrected age, the Bayley-III scores will be evaluated as secondary efficacy endpoints.

3.4.4.7 Laboratory Safety Analyses

Routine hematology and chemistry assessments will be conducted at the local laboratories according to each hospital's standard procedures. Only a limited number of extra blood samples will be taken from this vulnerable population for standard laboratory safety assessment. When a routine safety sample is collected during the treatment period, the following should whenever possible be determined in those samples: amylase, aminotransferases (alanine aminotransferase and aspartate aminotransferase), total bilirubin, sodium, and urea. Of these, amylase, aminotransferases, and total bilirubin have the highest priority.

Blood samples will be collected at the end of treatment (Day 29) for serum (or plasma) amylase, aminotransferases, total bilirubin, sodium, and urea, unless those have been determined in at least 1 blood sample collected following a minimum of 2 weeks of treatment with study drug. These analyses will be performed by the local laboratory.

Sample collection details and results will be recorded in the eCRF. The reporting of abnormalities as both laboratory findings and AEs should be avoided. An asymptomatic abnormal laboratory value should only be reported as an AE if it is clinically significant, if it fulfills a criterion for an SAE, or if it causes withdrawal of the patient from the study. If an abnormal laboratory value is associated with clinical signs and symptoms, the sign/symptom should be reported as an AE and the associated laboratory results should be considered additional information. Information regarding laboratory values judged by the investigator to be abnormal and clinically significant will be captured in the eCRF up to the 3-months visit. Information will also be captured until the values return to normal.

3.4.4.7.1 Vitamins A and D

A blood sample for the analysis of vitamin A and vitamin D, ie, D 25(OH)D_2 and 25(OH)D_3, including inactive epimer forms, in serum will be collected at Baseline (Day 1) and at the end of treatment (Day 29). Section 3.4.7 provides further details of sampling. Previously available methods for determination of vitamin D use serum volumes that are unacceptably high for the preterm infants. Therefore, an assay that can determine both vitamins A and D in the same sample has recently been developed at Sobi. This assay also includes determination of free cholesterol, since the levels of the lipophilic vitamins vary with the levels of serum

lipids. The new assay has an increased sensitivity relative to previously available methods, and thereby reduces the required blood volume.

3.4.4.7.2 rhBSSL Antibodies

A blood sample will be collected at Baseline (Day 1), at the end of treatment (Day 29), and at the 3-months follow-up visit for the determination of rhBSSL antibodies in serum. A further sample will be taken at the 6-months follow-up visit. Once the cut-off level for a positive antibody result has been defined (based on baseline data from approximately the first 50 patients), the 6-months sample will only be taken for those patients who have a positive result for the 3-months sample. If the result is positive at 6 months, a further sample will be collected at the 12-months-corrected-age follow-up visit. Section 3.4.7 provides further details of sampling.

3.4.5 Exploratory Assessments

The data collected for exploratory assessments may not be evaluated as a part of the main clinical study report, but reported separately.

3.4.5.1 Total Fatty Acids

See Section 3.4.3.6.

3.4.5.2 Body Composition

Body composition (percent fat mass), will be determined by air displacement plethysmography at study centers with access to the necessary equipment (Pea Pod®). Measurements will be performed at Baseline (Day 1), at the end of treatment (Day 29), at 40 weeks postmenstrual age, and 3 months corrected age, with the 2 latter visits scheduled only for patients with body composition assessments during the treatment period (Table 6–1 and Table 6–2).

3.4.5.3 Calprotectin and Microbiota

Calprotectin is a calcium-binding protein and it has been shown that levels of the protein are higher in preterm infants fed with fresh mother’s milk compared to infants fed with formula,

and that calprotectin excretion can be linked to the gut bacterial establishment (Rouge et al 2010; Savino et al 2010). However, the possible effect of rhBSSL on these variables is unknown.

A fecal sample will be collected at Baseline (Day 1) and at the end of treatment (Day 29). Detailed instructions regarding sampling and sample handling and shipping will be given in a separate laboratory manual. The samples will be analyzed with respect to calprotectin and bacterial contents.

3.4.6 Health Economic Assessments

3.4.6.1 Health Care Utilization

Information regarding health care utilization from the time of initial hospital discharge to home up to the 24-months-corrected-age visit will be collected through parent/primary caregiver reporting. The information will be collected as soon as informed consent has been provided (earliest at the 12-months-corrected-age visit), during telephone contacts at 15, 18 and 21 months corrected age and at the 24-months-corrected-age visit.

Parents/primary caregivers should be encouraged to use a notebook to keep track of all outpatient and inpatient visits and record information such as date, reason/diagnosis for visit, type of visit and duration of hospitalization. In case the caregiver is not able to provide information regarding health care utilization, the site will be asked to contact applicable health care providers for information. Approval from parents/primary caregivers to contact other health care providers will be collected as part of ICF procedure.

For outpatient care, the number of visits and type of health care provider will be recorded in the eCRF.

For inpatient care, the number of visits, reason/diagnosis for visit, number of days of hospitalization and number of days in the intensive care unit will be recorded in the eCRF.

The health care utilization information will not include standard preventive health care visits for children or vaccination programs.

3.4.6.2 Indirect Resource Use

Information regarding number of days lost from work related to the child´s condition from the time of initial hospital discharge to home until the 24-months-corrected-age visit will be collected through parent/primary caregiver reporting. The information will be collected as soon as informed consent has been provided (earliest at the 12-months-corrected-age visit), during telephone contacts at 15, 18 and 21 months corrected age and at the 24 months corrected age visit.

The number of days lost from work related to the child´s condition will be recorded in the eCRF. Parents/caregivers should be encouraged to use a notebook to keep track of the number of days lost from work.

3.4.6.3 Chronic Medical Conditions/Diagnoses

Chronic medical conditions/diagnoses present at 24 months corrected age will be recorded in the eCRF.

3.4.7 Sample Collections

Blood sampling for the care of the patients should always have priority over blood samples collected specifically for this study. No more than 1% of the total blood volume should be collected at any single time, and no more than 3% during a 4-week period. The total blood volume of the patients in this study is estimated to be 80 to 90 mL/kg of body weight (Hawcutt et al 2010), and 3% corresponds to 2.4 mL/kg of body weight. A visit window of ±3 days is allowed for all blood sampling after Baseline. This will allow study specific blood samples to be drawn at the same time point as standard routine samples minimizing the number of needle sticks.

Table 3–1 summarizes the whole blood volume that should whenever possible be collected for the scheduled samples for this study, including fatty acids (efficacy assessment [Section 3.4.3.6]) analyzed at the central laboratories.

Table 3–1 Maximum Blood Volumes to be Drawn from Each Patient (in Addition to Normal Routine Samples) During the Study Period

Assessment		Sample Time Point	Sample Volume (mL)	Total Volume During Study (mL)
Safety	Amylase<br>Aminotransferases<br>Total bilirubin<br>Sodium<br>Urea	Day 14 to 29[a]	Part of routine care sample	
	Vitamins A and D	Baseline<br>Day 29	0.1<br>0.1	0.2
	rhBSSL antibodies	Baseline<br>Day 29<br>Month 3<br>Month 6[b]<br>Month 12[b]	0.4<br>0.4<br>0.5<br>0.5<br>0.5	Maximum 2.3<br>(Less if negative result is confirmed at Month 3 and/or Month 6)
Efficacy	Fatty acids	Baseline<br>Day 29	0.25<br>0.5	0.75
**Total Blood Volume to be Drawn During the Study Period**				**3.25**

a. At least one sample collected during the treatment period Day 14 to Day 29.

b. Once cut-off level for a positive result has been defined, the sample is only collected for patients with a positive result at previous visit.

If it is not possible to collect the whole blood volume required according to the table, smaller volumes will be collected. The blood will then be used for as many of the assays as possible with the following priority order:

- rhBSSL antibodies
- Vitamins A and D
- Fatty acids

PPD will provide tubes and labels for the above samples. Further details regarding sampling and sample handling and shipping will be provided in a separate laboratory manual.

3.5 Overdose Management

An overdose is defined as any dose that is 10% or more than the dose prescribed (ie, in this case if a higher than prescribed concentration of rhBSSL is added to the food). Overdose may be symptomatic or asymptomatic. Symptoms associated with an overdose must be recorded as an AE (or SAE if criteria are met, Section 3.4.4.1.1) and the details provided to PPD PVG according to the procedures outlined in Section 3.4.4.1.3. An overdose without signs or symptoms must be documented in the study drug section of the eCRF.

3.6 Study Treatments

3.6.1 Method of Assigning Patients to Treatment Groups

Patients will be randomly assigned to receive either rhBSSL or placebo.

An interactive voice recognition system (IVRS) will be used for the randomization. Biostatistics will generate the randomization schedule for IVRS, which will link sequential patient randomization numbers to treatment codes. The randomization will be stratified by feeding regimen (PBM or infant formula) and by size for gestational age category (SGA or AGA). An infant having a birth weight that lies above the 10th percentile for the gestational age on the gender-specific intrauterine growth curves presented by Olsen et al 2010 is defined as AGA. An infant with a birth weight at or below the 10th percentile is defined as SGA. In this study at least 10% of the infants will be SGA at birth and at least 15% of the infants will be fed with PBM.

The randomization numbers will be blocked, and within each block the same number of patients will be allocated to each treatment group. The block size will not be revealed.

3.6.2 Treatments Administered

The patients will receive 8700 U of rhBSSL or placebo, depending on the randomization schedule, added to each 100 mL of the food. The treatment will start on the day of randomization or the day after. The day of treatment initiation is defined as Day 1 (Baseline). Treatment will continue for 4 weeks, until the Day 29 body weight has been recorded.

3.6.3 Identity of Investigational Product

Recombinant human BSSL will be delivered as a sterile powder for oral solution, in a single-dose glass vial containing 8700 U (corresponding to 15 mg active rhBSSL). The content of 1 vial is intended for reconstitution in 1 mL of sterile water before addition to 100 mL of food. Each vial includes an overfill to ensure that 8700 U can be transferred to 100 mL of food. The matching placebo will also be delivered as a sterile powder for oral solution in an identical single-dose container.

Sobi will provide the study drug to PPD who will package and label the study drug before distributing it to the study centers.

Table 3–2 presents the drug supplies that will be used in the study:

Table 3–2 Study Drug Supplies

Product	Supplied as:	Route
rhBSSL	Sterile powder for oral solution<br>Sterile water	Oral
Placebo	Sterile powder for oral solution<br>Sterile water	Oral

Abbreviation: rhBSSL, recombinant human bile-salt-stimulated lipase

The complete composition of rhBSSL drug product is presented in Table 3–3.

Table 3–3 Composition of rhBSSL Drug Product

Ingredient[a]	Quantity[b]	Function
rhBSSL	8700 U/vial	Active drug product
Sodium phosphate	1.8 mg/vial	Buffer
Sodium chloride	1.3 mg/vial	Solubility
Mannitol	43 mg/vial	Bulking agent
Glycine	3.9 mg/vial	Stabilizer

Abbreviation: rhBSSL, recombinant human bile-salt-stimulated lipase

a. Placebo has the same composition as the active drug product except it does not contain rhBSSL.

b. Each vial includes an overfill of 9% to ensure that 8700 U can be transferred to 100 mL of food.

3.6.4 Management of Clinical Supplies

3.6.4.1 Study Drug Packaging and Storage

The study drug, rhBSSL and placebo, will be stored in single-dose glass vials fitted with rubber stopper and sealed with flip tear-off aluminum caps.

The vials are packed together with sterile water for reconstitution of the powder. 1-mL oral/enteral syringes intended for administration of the reconstituted medicinal product to the food and syringe tip caps will be provided. The syringes and tip caps will be provided sterile and individually packed for single use.

Recombinant human BSSL drug substance is manufactured by WuXi AppTec Inc, (Philadelphia, Pennsylvania, United States) and drug product and matching placebo are manufactured by Patheon Italia SpA (Monza, Italy). PPD Ireland is responsible for storage and distribution in Europe and the PPD depot in Russia is responsible for storage and distribution in Russia.

The study drug must be stored refrigerated at 2°C to 8°C at the study center in a secure area. Once the study drug has been mixed with the food it is stable for up to 30 hours if kept refrigerated at 2°C to 8°C in a secure location. However, instructions for use for the specific formula and the unit's normal procedure must be considered when maximum length of storage is determined if it is shorter than 30 hours.

Since BSSL is deactivated at higher temperatures, care should be taken if heating the final mixture of rhBSSL and food before administration. While heating, **the food temperature should not exceed 37°C**, and preferably a temperature-controlled water bath should be used.

3.6.4.2 Preparation of Study Drug and Food

The pharmacist or delegate will mix the study drug with 1 mL of sterile water. If the reconstitution of study drug and the mixing of reconstituted study drug with the food is not done at the same location, the syringe can be transported with the tip cap in place. Each vial of study drug will be mixed with 100 mL of PBM or infant formula.

Each feeding should be drawn from the main food bottle, heated (**not above 37°C**) or given at room temperature, and the infant fed according to the unit's normal procedure. The main food bottle should not be heated and should remain refrigerated.

Additional details regarding the preparation of the food are provided in a separate study drug handling manual. Labels for rhBSSL have been designed according to regulatory and national requirements.

3.6.4.3 Study Drug Accountability

The investigator will maintain accurate records of receipt of all study drug, including dates of receipt. In addition, accurate records will be kept regarding when and how much study drug is added to food. The amount of drug administered to the patients will be estimated from the food records (Section 3.6.5.1). All used study drug vials should be stored in a secure area for drug accountability (room temperature). At the completion of the study all remaining study drug will be reconciled and returned, or destroyed locally according to applicable regulations.

3.6.5 Food Requirements

3.6.5.1 Daily Volume of Enteral Feeding

Before inclusion of the first patient, each study center must determine a target food volume, to be used for all infants in the study. This volume should be in the range of 150 to 180 mL/kg/day. Once an infant has reached the target volume, the food volume should not be changed during the treatment period. Reasons for departure from the determined target volume must be recorded in the eCRF.

The daily volume (mL) of food enterally fed to the patient will be recorded in the eCRF every day during the treatment period (Table 6–1). The volume recorded should take into account the food that remained in the bottle at the end of feeding.

3.6.5.2 Daily Volume of Parenteral Nutrition

The total daily volume (mL) of parenterally administered nutrition received by the patient during the treatment period will be recorded in the eCRF (Table 6–1). The product name and

volume of any parenterally administered nutrition containing a fat emulsion should also be recorded.

3.6.5.3 Infant Formula Requirements

Each study center will select one infant formula that all formula fed patients at that study center will be fed. They will remain on the same infant formula throughout the treatment period, unless medically contraindicated. The exact brand name of the infant formula used should be recorded in the eCRF to enable the composition to be derived.

The range of protein, carbohydrate, and lipid content for the infant formulas allowed for use in this study are

Protein: 2.8 to 4.1 g/100 kcal

Carbohydrate: 9.5 to 12.0 g/100 kcal

Lipids: 4.4 to 6.0 g/100 kcal

In addition, all infant formulas to be used in the study must contain AA and DHA. The infant formula must contain less than or equal to 40% of medium-chain TGs.

3.6.5.4 Pasteurized Breast Milk Requirements

There are no special requirements with regard to the source or quality of the PBM. The milk may be the mother's own or come from a milk bank (donor milk). Information about the predominant milk type will be recorded in the eCRF. The composition of the milk (fat, protein, and lactose) should be recorded when available. Fortification of the milk should be done according to a predefined study center-specific schedule. Preferably, one fortifier should be used at a study center, and the amounts added should be fixed, i.e., the same concentration added to all PBM. Fortification must not be individualized based on body weight. All use of fortifiers, including lipid emulsions, should be recorded in the eCRF.

The availability of PBM varies between NICUs, and thus the time period during which an infant is given only PBM before switching to infant formula will vary. In this study, the time of switching to infant formula must not be based on body weight. Instead, each study center

using PBM in this study should, before the first infant is randomized, declare which of the following alternatives will be followed for the infants in the study:

1. PBM only for 4 weeks
2. PBM only for 2 weeks followed by infant formula for 2 weeks

The date when infant formula is first introduced should be recorded in the eCRF, as well as the volumes of PBM and, if applicable, infant formula.

3.6.5.5 Concomitant Use of Fresh Breast Milk

No fresh breast milk should be used during the 29 days of treatment. Any deviation from this rule must be noted in the eCRF. However, for ethical reasons, breast feeding cannot be prohibited. During the treatment period, the infants may be put to the breast, and the consumption of small volumes is allowed. If this is the case the date when fresh breast milk was first consumed will be recorded in the eCRF.

3.6.6 Blinding

Recombinant human BSSL and placebo will be administered in a double-blind fashion. The randomization schedule will be maintained in a secure, locked location by PPD and will not be revealed to any study center personnel, investigators, sponsor personnel, PPD personnel, or the patient's legally acceptable representatives until after the database lock has been achieved. The PPD independent statistical team supporting the data safety monitoring board (DSMB) will have access to the randomization schedule (Section 5.4).

3.6.7 Breaking the Blind

3.6.7.1 Emergency Unblinding

The PPD medical monitor will be responsible for maintaining the blind throughout the study. If a patient becomes seriously ill, the blind will be broken only if knowledge of the administered study drug will affect treatment options available to the patient.

At the initiation of the study, study centers will be instructed on the method for breaking the blind. This will be an electronic process via the IVRS, which is fully documented in the

IVRS manual. Blinding codes should only be broken in emergency situations for reasons of patient safety. The investigator should contact PPD, who will contact the sponsor before breaking the blind. In an emergency when there is insufficient time for the investigator to contact PPD or the sponsor, the investigator is permitted to break the blind without contacting PPD or the sponsor. The sponsor and PPD must be notified as soon as possible after the blind break. Only the IVRS user who is logged in during the emergency code break session will receive an unmasked copy of the notification in real time. All other users (clinical team and sponsor team) will receive a blinded real-time notification informing them of the code break.

3.6.7.2 Scheduled Unblinding

The blind will be broken for an independent analysis and reporting team within PPD and for Sobi once the last patient has completed the 3-month visit and 200 patients have completed the 12-months-corrected-age visit, including the Bayley assessment. Once unblinding has occurred, the unblinded teams will not be involved in decisions regarding individual patients where the patient identity can be revealed. The blind will be maintained for all other teams within PPD, personnel at the sites, and the patients' legally authorized representatives until the end of the study. For details of study reporting, see Section 5.8.

3.6.8 Treatment Compliance

Measures of treatment compliance are not applicable for this study.

3.6.9 Prior, Concomitant, and Subsequent Therapy

Other therapy considered necessary for the patient's welfare may be given at the discretion of the investigator. All such therapy must be recorded in the eCRF. The patient is not allowed to participate concurrently in another clinical intervention study and as such may not use any drug or treatment concurrently under investigation until the patient has completed the 12-months-corrected-age visit.

Preterm infants often experience complications that need therapeutic intervention. This can in most cases be accepted as long as the medication does not interfere with feeding.

All use of concomitant and subsequent medications, from the start of the study up to the 3-months follow-up visit, will be recorded in the patient's eCRF (Table 6–1 and Table 6–2). This will include all prescription drugs, herbal products, vitamins, minerals, and over-the-counter medications. Any changes in concomitant medications also will be recorded in the patient's eCRF.

3.7 Statistical Analysis Plan

A detailed statistical analysis plan will be prepared for this study before the clinical database is locked and should be referred to for further details of the statistical analysis.

3.7.1 Primary Efficacy Endpoints

The primary efficacy variable is growth velocity in grams per kilogram per day during 4 weeks of treatment.

3.7.2 Secondary Efficacy Endpoints

In addition to the primary efficacy endpoint, the following secondary efficacy endpoints will be evaluated to support the primary objective:

- Change from Baseline in body weight (g) at 3 months
- Body weight (g) at 12 months corrected age
- Change from Baseline in total body length (mm) at 4 weeks and 3 months
- Body length (mm) at 12 months corrected age

The following secondary efficacy endpoint will be evaluated to support the objective "To determine the effect of rhBSSL treatment in decreasing risk of growth restriction":

- Growth restriction, defined as growth velocity <15 g per kilogram bodyweight per day during 4 weeks of treatment

The following secondary efficacy endpoints will be evaluated to support the objective "To determine the effect of rhBSSL treatment in shortening the time of hospital stay":

- Time to readiness for discharge. This endpoint will be defined as the time from Baseline when all of the following criteria have been fulfilled: a sustained weight gain, ability to maintain normal body temperature, ability to suckle feed, and ability to maintain stable cardiorespiratory function.
- Time to discharge

The following secondary efficacy endpoints will be evaluated to support the objective "To determine the effect of rhBSSL treatment in improving early development":

- Change from Baseline in head circumference (mm) at 4 weeks and 3 months
- Head circumference (mm) at 12 months corrected age
- Time from Baseline to 150 mL/kg/day of enteral feeding

The following secondary efficacy endpoint will be evaluated to support the objective "To determine the effect of rhBSSL treatment on decreasing readmittance to hospital":

- Readmission to hospital within 1 month of discharge

The following secondary efficacy endpoints will be evaluated to support the objective "To determine the effect of rhBSSL treatment on increasing the levels of DHA and AA":

- Levels of DHA and AA at 4 weeks

The following secondary efficacy endpoints will be evaluated to support the objective "To determine the effect of rhBSSL treatment on neurodevelopment":

- Bayley-III scores at 24 months corrected age
  - Cognitive domain composite score
  - Language domain composite score
  - Motor domain composite score
  - Social-emotional domain composite score
  - Adaptive domain composite score

  - Scaled scores for each subtest and for the cognitive and social-emotional domains (in total 16 scaled scores)

- Neurodevelopment disability composite at 24 months corrected age (as defined in section 3.4.3.9)
- CBCL scores at 24 months corrected age
  - Scores for each of 7 syndrome sub-scales (emotionally reactive, anxious/depressed, somatic complaints, withdrawn, sleep problems, attention problems and aggressive behavior)
  - Score for internalizing problems
  - Score for externalizing problems
  - Score for total problems

The following secondary efficacy endpoints will be evaluated to support the objective "To determine the long-term effect of rhBSSL treatment on anthropometrics":

- Body weight (g) at 24 months corrected age
- Body height (cm) at 24 months corrected age
- Head circumference (mm) at 24 months corrected age

3.7.3 Safety Endpoints

The study has the following secondary safety endpoints to meet the objective "To compare the safety and tolerability of rhBSSL treatment in preterm infants with that of placebo treatment after oral administration in infant formula or PBM":

- AEs up to 3 months and SAEs up to 12 months corrected age
- Bayley-III scores at 12 months corrected age
  - Cognitive domain composite score
  - Language domain composite score
  - Motor domain composite score

    - Scaled scores for each subtest and for the cognitive domain (in total 5 scaled scores)

- Vomiting (frequency and volume)
- Vital signs (heart rate, blood pressure, body temperature)
- Levels of amylase, bilirubin, aminotransferases, sodium, and urea
- Levels of vitamins A and D
- Presence of rhBSSL antibodies

The study has the following secondary safety endpoints to meet the objective "To determine the long-term safety of rhBSSL treatment":

- Serious ADRs up to 24 months corrected age

3.7.4 Health Economic Endpoints

The study has the following health economic endpoints to meet the objective "To assess the effect of rhBSSL treatment on health care utilization":

- The number of outpatient visits from the time of initial hospital discharge to home up to 24 months corrected age
- The number of inpatient visits, days of hospitalization and days in intensive care unit from the time of initial hospital discharge to home up to 24 months corrected age

The study has the following endpoint to meet the objective "To assess the effect of rhBSSL treatment on indirect resource use":

- Number of days lost from work related to the child´s condition from the time of initial hospital discharge to home up to 24 months corrected age

The study has the following endpoint to meet the objective "To assess the effect of rhBSSL treatment on chronic medical conditions/diagnoses":

- Presence of chronic medical conditions/diagnoses at 24 months corrected age

3.7.5 Exploratory Endpoints

The study has the following exploratory endpoints:

- Levels of fatty acids
- Percent body fat
- Fecal calprotectin concentration
- Fecal microbiota

3.7.6 Sample Size Calculations

Assuming a true difference in growth velocity between rhBSSL and placebo of 2.25 g/kg/day at 4 weeks and a standard deviation of 7 g/kg/day, 205 evaluable patients in each treatment arm provide 90% power to demonstrate that rhBSSL improves growth velocity compared with placebo (using a 2-sided test with a significance level of 5%). Allowing for 5% of patients to be excluded from the FAS, it is estimated that 432 patients will be randomized. These assumptions are based on the meta-analysis of Sobi's 2 previous Phase 2 studies; the estimate of standard deviation has been increased by 30% to allow for greater variability within the Phase 3 study population.

The sample size calculation was performed using nQuery Advisor software Version 4.0 (Statistical Solutions, Ltd, Cork, Ireland).

3.7.7 Analysis Sets

The following analysis sets will be used in the statistical analyses.

Full-analysis set: The FAS is the primary analysis set and will consist of all patients randomly assigned to treatment who have a baseline and at least 1 postbaseline assessment of body weight. All analyses using the FAS will group patients according to randomized treatment.

Per-protocol set (PPS): The PPS is the secondary analysis set and will consist of all FAS patients who receive the treatment according to randomization, have no major protocol violations, and remain in the study for a minimum of 1 week. Further criteria may be specified in the statistical analysis plan.

Safety set: The safety set will consist of all patients who received at least 1 dose of study drug. All analyses using the safety set will group patients according to treatment actually received.

The FAS will be used as the primary population and the PPS as supportive for the analyses of the primary efficacy variable, the FAS will be used for the secondary efficacy variables, and the safety set will be used for the safety analysis.

3.8 Statistical Analysis

Demographic data and background characteristics will be summarized using descriptive statistics. Continuous variables will be summarized using the mean, the standard error of the mean, the median, the minimum value, and the maximum value. Categorical variables will be summarized using frequency counts and percentages. All data will be listed in data listings.

Details of all analyses will be described in the statistical analysis plan.

Statistical analysis will be performed using SAS software Version 9.1 or later (SAS Institute, Inc, Cary, North Carolina, United States). All statistical tests will be 2-sided and performed using a 5% significance level, leading to 95% (2-sided) confidence intervals.

3.8.1 Primary Efficacy Analysis

The null and alternative hypotheses are defined for the primary efficacy variable (growth velocity during the 4 weeks of treatment):

H_0: $\mu_{\text{rhBSSL}} = \mu_{\text{placebo}}$

H_A: $\mu_{\text{rhBSSL}} \neq \mu_{\text{placebo}}$

In order to calculate growth velocity, the natural log-transformed value of the baseline and all postbaseline weight assessment for each patient will be calculated. A linear regression model will then be fitted for each patient with a response variable of log(weight) and a predictor variable of time. Growth velocity for each patient will be estimated as the slope arising from the regression model, and will need to be multiplied by 1000 for conversion into the desired unit of grams per kilogram per day. The weight over time for each treatment group will be

illustrated graphically and if the log-transformed data clearly deviate from the linearity assumption, a sensitivity analysis will be performed using untransformed data.

Statistical analysis for the primary efficacy variable will be performed using an analysis of covariance model including factors for treatment, feeding regimen (PBM or infant formula), size for gestational age category (SGA or AGA), with baseline weight included as a covariate. For the comparison between treatment groups, the point estimate and the associated 95% confidence interval and *P* value from the model will be presented.

The assumptions of normality and homogeneity of variance will be assessed by inspection of normal probability plots and residual plots. If either assumption is clearly not met, a corresponding nonparametric analysis will be performed (using the same analysis of covariance model, but using ranked response and covariate) to check the robustness of the results.

It is anticipated that the withdrawal rate from the study after randomization will be approximately 15%. For the primary analysis, if patients withdraw before 4 weeks then growth velocity will be derived using weight assessments up to their last available assessment. Sensitivity analyses will be conducted to assess the impact of missing data.

The methods for handling of missing data will be fully described in the SAP.

The primary efficacy variable will be analyzed using both the FAS (the primary population for efficacy evaluation) and PPS (supportive).

3.8.2 Secondary Efficacy Analyses

The secondary efficacy variables will be analyzed using the FAS only.

The following will be analyzed using an analysis of covariance approach similar to that used for the primary endpoint. No logarithmic transformation will be performed for these endpoints.

- Change from Baseline in body weight (g) at 3 months,
- Body weight (g) at 12 months corrected age,

- Change from Baseline in total body length (mm) at 4 weeks and 3 months,
- Body length (mm) at 12 months corrected age,
- Change from Baseline in head circumference (mm) at 4 weeks and 3 months,
- Head circumference (mm) at 12 months corrected age

The following will be analyzed using descriptive statistics:

- Body weight (g) at 24 months corrected age
- Body height (cm) at 24 months corrected age
- Head circumference (mm) at 24 months corrected age
- Weight-to-length ratio (g/cm) at 4 weeks

The growth restriction is defined as a growth velocity of less than 15 g per kilogram bodyweight per day during the 4-week treatment period and will be analyzed using a logistic regression model with treatment, feeding regimen (PBM or infant formula), and size for gestational age category (SGA/AGA) as explanatory variables. Based on this model, the estimated risk of growth restriction in the rhBSSL and placebo groups respectively, the estimated odds ratio, the associated 95% confidence interval and P-value will be presented. If the risk of growth restriction in the rhBSSL group is statistically significantly lower than in the placebo group, it will be concluded that rhBSSL reduces the risk of growth restriction in preterm infants receiving infant formula or PBM. In addition, the number and proportion of patients in each treatment group with growth restriction will be presented for all patients as well as by feeding regimen and by size for gestational age category.

Time to readiness for discharge will be analyzed using an analysis of variance model. Time to readiness for discharge will be used as the response variable and factors will be included for treatment, feeding regimen (PBM or infant formula) and size for gestational age category (SGA or AGA). For the comparison between treatment groups, the point estimate, the associated 95% confidence interval and P-value from the model will be presented.

Time to discharge will be analyzed using an analysis of variance model similar to that used for time to readiness for discharge.

Readmission to hospital within 1 month of discharge will be analyzed using a logistic regression model with treatment, feeding regimen (PBM or infant formula), and size for gestational age (SGA/AGA) as explanatory variables. Based on this model, the estimated risk of readmission to hospital in the rhBSSL and placebo groups respectively, the estimated odds ratio and the associated 95% confidence interval will be presented. In addition, the number and proportion of patients in each treatment group who were readmitted to hospital will be presented for all patients as well as by feeding regimen and by size for gestational age category.

Time from start of treatment to 150 mL/kg/day of enteral feeding will be presented using descriptive statistics.

Statistical analysis of the Bayley-III cognition, language, motor, social-emotional and adaptive behavior composite scores at 24 months corrected age will be performed using an analysis of covariance model including factors for treatment, feeding regimen (PBM or infant formula), size for gestational age category (SGA or AGA), and critical confounders for neurodevelopment included as covariates. Based on this model, the estimated mean score for each treatment group, the estimated difference between treatments and the associated 95% confidence interval will be presented. The critical confounders to be included in the model will be specified in a study-specific statistical analysis plan finalized prior to breaking the blind. In addition, the respective domain composite score and the scaled score for each subtest at 24 months corrected age will be presented by descriptive statistics.

The neurodevelopment disability composite outcome will be analyzed using a logistic regression model including treatment, feeding regimen (PBM or infant formula), size for gestational age category (SGA or AGA) and critical confounders for neurodevelopment included as explanatory variables. Based on this model, the estimated risk of a neurodevelopment disability in each treatment group, the estimated odds ratio for rhBSSL to placebo and the associated 95% confidence interval will be presented. The critical confounders to be included in the model will be specified in a study-specific statistical analysis plan finalized prior to breaking the blind. Furthermore, the number and proportion of patients in each treatment group with a neurodevelopment disability (according to the above

scale) will be presented for all patients as well as by feeding regimen and by size for gestational age category. This latter presentation will also be done for each individual component of the composite for all patients and by feeding regimen and by size for gestational age category.

The CBCL scores will be analyzed using descriptive statistics.

3.8.3 Health Economic Endpoints

Health economic endpoints will be analyzed using descriptive statistics.

3.8.4 Exploratory Endpoints

Exploratory endpoints will be analyzed using descriptive statistics.

3.8.5 Subgroup Analyses

In order to examine the consistency of the treatment effect, the primary analysis will be repeated within predefined subgroups in the FAS. These subgroups will include the randomization strata. For each subgroup, the estimated growth velocity in each treatment group, the estimated difference in growth velocity between treatments and the corresponding 95% confidence intervals will be presented. *P* values will not be presented. The relationship between growth velocity and gestational age at birth will be assessed and presented graphically for each treatment.

Further subgroup analyses may be defined in the statistical analysis plan.

3.8.6 Safety Analyses

Safety analysis will be performed for vital sign measurements, laboratory safety results, tolerability, withheld enteral feeding during 24 hours, ECG findings, vitamin A and D levels, antibodies against rhBSSL, Bayley-III (composite scores for the cognitive, language and motor domains at 12 months corrected age), and AE data collected up to 3 months and SAE data up to 12 months corrected age.

A separate safety analysis will be performed for serious ADRs collected up to 24 months corrected age.

3.8.6.1 Adverse Events

All AEs will be coded using the latest version of MedDRA. All treatment-emergent AEs will be summarized showing the number of patients who experienced an event, the percentage of patients with that event, and the severity, duration, and relationship to study drug. Percentages will be based on the number of patients who received each treatment during the study.

The incidence of SAEs, including death, serious ADRs and AEs leading to study drug discontinuation will also be tabulated.

3.8.6.2 Clinical Laboratory Results

Clinical laboratory safety test data will be presented using summary statistics. Laboratory normal ranges are not available for preterm infants. Values will be assessed for clinical significance by the investigator and all clinically significant abnormal results will be entered as AEs in the source documents and the eCRF. The number and proportion of patients experiencing clinically significant events during the treatment period will be presented.

3.8.7 Interim Analyses

In order to establish whether the assumptions used to calculate the sample size are appropriate, an blinded interim analysis (IA) will be conducted once 200 patients have been randomized and followed up to 4 weeks or discontinuation (whichever occurs first). At the IA, growth velocity will be calculated for each patient. The overall standard deviation of the growth velocity data will be calculated, and the within-group standard deviation will be estimated using the assumption that the treatment effect is an increase in mean growth velocity of 2.25 g/kg/day as anticipated in the sample size calculations. The sample size calculation will be re-performed using the estimated within-group standard deviation, with all other assumptions remaining unchanged. Sobi will use the IA results to decide whether the sample size of the study should be increased in order to ensure the primary objective can be reliably assessed. The sample size will be increased up to a maximum of 534 evaluable patients, which corresponds to 564 randomized patients assuming a withdrawal rate of 5% from the FAS. The proposed maximum corresponds to a standard deviation of 8.0 g/kg/day. Regardless of the IA result, the sample size will not be decreased, or increased beyond the proposed maximum. As this is a blinded analysis, there is no impact on the overall power or

Type I error rate. Data contributing to the IA will be fully cleaned, and a full analysis plan for the IA will be written before the analysis is conducted.

The DSMB will review selected safety data on an ongoing basis (Section 5.4).

3.8.8 Analysis of Data Up to 12 Months

All available data up to the 12-months-corrected-age visit will be analyzed when all patients have completed the 3-month visit and 200 patients have completed the 12-months-corrected-age visit, including the Bayley assessment. The results will be reported in a clinical study report. When all patients have completed their 12-month visit, complementary analyses of 12-month data (ie, after the 3-month visit and up to the 12-month visit) will be performed and reported in an addendum to the clinical study report.

3.8.9 Analysis of 24-months Data

The analysis of the 24-months-corrected-age assessments, as well as the health economic assessments and any serious ADRs will be performed when the last patient has completed the 24-months-corrected-age visit. The results of this analysis will be reported in a separate clinical study report.

3.8.10 Combining Data

A prospective protocol with the purpose of combining data from this study with data from other possible future studies will, if applicable, be written.

3.9 Data Quality Assurance

During study conduct, PPD will conduct periodic monitoring visits to ensure that the protocol and Good Clinical Practice are being followed. The monitors will review source documents to confirm that the data recorded in eCRFs are accurate. The investigators and institutions will allow PPD monitors and appropriate regulatory authorities access to source documents to perform this verification.

The study center may be subject to review by the independent ethics committee (IEC) and to quality assurance audits performed by Sobi, or companies working with or on behalf of Sobi, and to inspection by appropriate regulatory authorities.

Investigators and their relevant personnel must be available during the monitoring visits and possible audits or inspections and sufficient time must be devoted to the process.

4 Investigator's Obligations

The following administrative items are meant to guide the principal investigator or subinvestigator in the conduct of the study but may be subject to change based on industry and government standard operating procedures, working practice documents, or guidelines. Changes will be reported to the IEC but will not result in protocol amendments.

4.1 Confidentiality

All laboratory specimens, evaluation forms, reports, and other records will be identified in a manner designed to maintain patient confidentiality. All records will be kept in a secure storage area with limited access. Clinical information will not be released without the written permission of the patient's legally acceptable representative, except as necessary for monitoring and auditing by the sponsor, the sponsors designee, the regulatory authorities, or the IEC.

The principal investigator or subinvestigator and all employees and coworkers involved with this study may not disclose or use for any purpose other than performance of the study, any data, record, or other unpublished, confidential information disclosed to those individuals for the purpose of the study. Prior written agreement from the sponsor or its designee must be obtained for the disclosure of any said confidential information to other parties.

4.2 Institutional Review

Regulations and the ICH guidelines require that approval be obtained from an IEC before participation of human patients in research studies. Before study onset, the protocol, ICF, advertisements to be used for the recruitment of study patients, and any other written information regarding this study to be provided to the patient's legally acceptable representative must be approved by the IEC. Documentation of all IEC approvals and of the

IEC compliance with ICH harmonised tripartite guideline E6(R1): Good Clinical Practice will be maintained by the study center and will be available for review by the sponsor or its designee.

All IEC approvals will be signed by the IEC chairman or designee and must identify the IEC name and address, the clinical protocol by title or protocol number or both, and the date approval or a favorable opinion was granted.

The principal investigator or subinvestigator is responsible for obtaining continued review of the clinical research at intervals not exceeding 1 year or otherwise specified by the IEC. The principal investigator or subinvestigator must supply the sponsor or its designee with written documentation of continued review of the clinical research.

4.3 Informed Consent

Before being admitted to the clinical study, the patient’s legally acceptable representative must have expressed his or her consent to participate, after clear explanations about the nature, scope, and possible consequences of the clinical study have been given to them by the investigator or designee.

Informed consent will also be collected from the patient´s legally acceptable representative for the 24-months-corrected-age assessments, as well as the health economic assessments and any serious ADRs. This consent will be collected when amendment 2 has been implemented and earliest at the 12-months-corrected-age visit. In addition, informed consent will be collected from the legally acceptable representatives for the collection of confounders of neurodevelopment.

If consent to continue into the extension study is refused then data will be collected as per the original protocol, up to and including the 12-months-corrected-age visit. The patient will follow the end of study procedures as described in the original protocol and no further data will be collected.

Information will be given in both oral and written form. The informed consent information sheets will include all the elements required by law following the ICH E6(R1) guidelines. The informed consents will be approved by the IEC (and regulatory authorities) of each study center.

In addition to the standard requirements that physicians are currently obliged to observe when providing information, the following points must also be covered:

- A description of the objectives of the study and how it will be organized
- The type of treatment
- Any potential negative effects attributable to the study drug
- The freedom to ask for further information at any time
- The patient’s legally acceptable representative’s right to withdraw the patient from the clinical study at any time without giving reasons and without jeopardizing the patient’s further course of medical treatment
- The existence of patient insurance coverage and a summary of what is included in this coverage

Adequate time and opportunity to satisfy questions will be given to the patient’s legally acceptable representative.

The investigator will be supplied with an adequate number of ICFs to be used. The forms will be signed and dated by both the investigator or designee and the patient’s legally acceptable representative before the beginning of the study. A copy of the signed forms will be given to the patient’s legally acceptable representative.

To ensure medical confidentiality and data protection, the signed ICFs will be stored in the investigator's study file. The investigator will allow inspection of the forms by authorized representatives of the sponsor, IEC members, and regulatory authorities. The investigator will confirm, by signing and dating the eCRFs, that informed consent has been obtained.

4.4 Study Reporting Requirements

By participating in this study the principal investigator or subinvestigator agrees to submit reports of SAEs according to the time line and method outlined in the protocol. In addition, the principal investigator or subinvestigator agrees to submit annual reports to his or her IEC as appropriate. The principal investigator or subinvestigator also agrees to provide the sponsor with an adequate report shortly after completion of the principal investigator’s or subinvestigator’s participation in the study.

4.5 Financial Disclosure and Obligations

Principal investigators or subinvestigators are required to provide financial disclosure information to allow the sponsor to submit the complete and accurate certification or disclosure statements. In addition, the principal investigator or subinvestigators must provide to the sponsor a commitment to promptly update this information if any relevant changes occur during the course of the investigation and for 1 year following the completion of the study.

Neither the sponsor nor PPD is financially responsible for further testing or treatment of any medical condition that may be detected during the screening process. In addition, in the absence of specific arrangements, neither the sponsor nor PPD is financially responsible for further treatment of the patient's disease.

4.6 Investigator Documentation

Prior to beginning the study, the investigator will be asked to comply with ICH E6(R1) 8.2 by providing the following essential documents, including but not limited to the following:

- An original investigator-signed investigator agreement page of the protocol.
- An IEC-approved ICF and any other written information regarding this study that is to be provided to the patient's legally acceptable representative.
- IEC approval.
- Curriculum vitae for the principal investigator and each subinvestigator. Current licensure must be noted on the curriculum vitae. They will be signed and dated by the principal investigators and subinvestigators at study start-up, indicating that they are accurate and current.
- Financial disclosure information to allow the sponsor to submit complete and accurate certification or disclosure statements. In addition, the investigators must provide to the sponsor a commitment to promptly update this information if any relevant changes occur during the course of the investigation and for 1 year after the completion of the study.
- Laboratory certifications and normal ranges for any local laboratories used by the study center.

4.7 Study Conduct

The principal investigator agrees that the study will be conducted according to the principles of the ICH E6(R1). The principal investigator will conduct all aspects of this study in accordance with all national and local laws or regulations.

4.8 Data Collection

4.8.1 Case Report Forms and Source Documents

It is the intent of this study to acquire study data via electronic format. As part of the responsibilities assumed by participating in the study, the principal investigator or subinvestigator agrees to maintain adequate case histories for the patients treated as part of the research under this protocol. The principal investigator or subinvestigator agrees to maintain source documentation (eg, laboratory reports, ECG strips), to enter patient data into the eCRF as accurately as possible, and to respond to any reported discrepancies rapidly.

The eCRFs are accessed through the Oracle Clinical Remote Data Capture application (Oracle Corp, Redwood Shores, California, United States), which allows for study center data entry and data management. Study center users can read from and write to the sponsor's database where the clinical data are collected. This provides immediate, direct data transfer to the database, as well as immediate detection of discrepancies, enabling study center coordinators to resolve and manage discrepancies in a timely manner.

Each person involved with the study at each study center will have an individual log-on and password that allow for record traceability. Thus, the system, and subsequently any investigative reviews, can identify coordinators, investigators, and individuals who have entered or modified records.

4.9 Adherence to Protocol

The investigator agrees to conduct the study as outlined in this protocol in accordance with the ICH E6(R1) and all applicable guidelines and regulations.

4.10 Coding Dictionaries

Medical history, chronic medical conditions/diagnoses as well as all AEs, will be coded using MedDRA. Previous and concomitant medications will be coded using the World Health Organization Drug Dictionary.

Versions of coding dictionaries used will be stated in the study report.

4.11 Investigator's Final Report

Upon completion of the study, the investigator, where applicable, should inform the institution; the investigator or institution should provide the IEC with a summary of the study's outcome, and the sponsor and regulatory authorities with any reports required.

4.12 Records Retention

All correspondence (eg, with sponsor, IEC, or clinical research associates) relating to this clinical study will be kept in appropriate file folders by the principal investigator. Records of patients, source documents, eCRFs, and drug inventory sheets pertaining to the study must be kept on file by the principal investigator.

Essential documents will be retained by the investigators for a period of 10 years from the close out visit or according to local legislation (whichever is the longest) or at least 2 years have elapsed since the formal discontinuation of clinical development of the investigational product. These documents will be retained for a longer period, however, if required by the applicable regulatory requirements or by an agreement with the sponsor. It is the responsibility of the sponsor to inform the principal investigator or subinvestigator/institution as to when these documents no longer need to be retained.

If an investigator moves, withdraws from an investigation, or retires, the responsibility for maintaining the records may be transferred to another person, who will accept the responsibility. Notice of transfer must be made to and agreed upon by the sponsor.

4.13 Publications

Sobi will publically register this study through posting the protocol and results of this study at www.clinicaltrials.gov.

The results of this study will be published within 1 year after the first regulatory approval of the product. If drug development is discontinued before approval, the results will be published within 1 year after such discontinuation.

After completion of the study, the data may be considered for reporting at scientific meetings or for publications in scientific journals. In these cases, the sponsor will be responsible for these activities and will work with the investigators to determine how the manuscript is written and edited, the number and order of authors, the publication to which it will be submitted, and other related issues. The sponsor has final approval authority over all such issues and will ensure any publication is written according to appropriate guidelines (Wager et al 2003).

Data are the property of the sponsor and cannot be published without prior authorization from the sponsor, but data and publication thereof will not be unduly withheld.

5 Study Management

5.1 Sponsor

Anna Olsson
Clinical Study Manager
Swedish Orphan Biovitrum AB (publ)
112 76 Stockholm
Sweden

5.2 Vendor Contact

PPD Global
PPD Granta Park
Great Abington,
Cambridge CB21 6GQ
United Kingdom

SAE reporting
Medical Affairs/Pharmacovigilance
PPD Granta Park
Great Abington,
Cambridge CB21 6GQ
United Kingdom
SAE Hotline: +44 1223 374240
SAE Fax line: +44 1223 374102
SAE E-mail: emeaasiasafetycentral.sm@ppdi.com

5.3 Central Laboratories

rhBSSL Antibodies and Vitamins A and D

York Bioanalytical Solutions Ltd
Cedar House
Northminster Business Park
Upper Poppleton
York YO26 6QR
United Kingdom

Fatty Acids

Nutrition Research Program
Child and Family Research Institute
Room 171B
950 West 28th Avenue
Vancouver
British Columbia V5Z4H4
Canada

Fecal Calprotectin and Microbiota

PPD Central Laboratory
2 Tesseneer Drive
Highland Heights, KY 41076
USA

5.4 Data Safety Monitoring Board

Review of unblinded safety data will be conducted by an independent DSMB. The DSMB's membership, responsibilities, meeting details, and relationship with other members of the study team are defined in the charter. The membership of the DSMB will include an independent statistician and clinicians (including at least 1 neonatologist) that, collectively, have experience in the management of preterm infants, potential side effects of this study, and in the conduct of randomized clinical trials.

The DSMB will be independent from the sponsor, and the blind will be broken for the members of the DSMB only to aid interpretation of the data. Unblinded safety analyses will be provided for the DSMB by a statistical team that is independent of the study team, with strict procedures to preserve the study blind.

5.5 Monitoring

5.5.1 Monitoring of the Study

The monitor, as a representative of the sponsor, has the obligation to follow the study closely. In doing so, the monitor will visit the principal investigator or subinvestigator and study facilities at periodic intervals, in addition to maintaining necessary telephone and letter contact. The monitor will maintain current personal knowledge of the study through observation, review of study records and source documentation, and discussion of the conduct of the study with the principal investigator or subinvestigator and staff.

All aspects of the study will be carefully monitored, by the sponsor or its designee, for compliance with applicable government regulation with respect to current Good Clinical Practice and current standard operating procedures.

5.5.2 Inspection of Records

Principal investigators or subinvestigators and institutions involved in the study will permit trial-related monitoring, audits, IEC review, and regulatory inspections by providing direct access to all study records. In the event of an audit, the principal investigator or subinvestigator agrees to allow the sponsor, representatives of the sponsor, or regulatory agencies access to all study records.

The principal investigator or subinvestigator should promptly notify the sponsor and PPD of any audits scheduled by any regulatory authorities and promptly forward copies of any audit reports received to the sponsor.

5.6 Management of Protocol Amendments and Deviations

5.6.1 Modification of the Protocol

Any changes in this research activity, except those necessary to remove an apparent, immediate hazard to the patient, must be reviewed and approved by the sponsor or its designee. Amendments to the protocol must be submitted in writing to the principal investigator's or subinvestigator's IEC for approval before patients being enrolled into an amended protocol. This will be fully documented.

The investigator must not implement any deviation from or change to the protocol without discussion with and agreement from Sobi, and prior review, documented approval and favorable opinion of the amendment from the relevant IEC and/or regulatory authorities, except where it is necessary to eliminate an immediate hazard to patients or where the changes involve only logistical or administrative aspects of the clinical study.

Protocol amendments will be submitted to the appropriate authorities as required by the applicable regulatory requirements.

5.6.2 Protocol Violations and Deviations

The principal investigator or subinvestigator or designee must document and explain in the patient's source documentation any deviation from the approved protocol. The principal investigator or subinvestigator may implement a deviation from, or a change of, the protocol to eliminate an immediate hazard to trial patients without prior IEC approval. As soon as possible after such an occurrence, the implemented deviation or change, the reasons for it, and any proposed protocol amendments will be submitted to the IEC for review and approval, to the sponsor for agreement, and to the regulatory authorities, if required.

A deviation from the protocol is an unintended or unanticipated departure from the procedures or processes approved by the sponsor and the IEC and agreed to by the principal investigator or subinvestigator. Deviations usually have an impact on individual patients or a small group of patients and do not involve inclusion, exclusion, or primary endpoint criteria. A protocol violation occurs when there is nonadherence to the protocol that results in a significant, additional risk to the patient, when the patient's legally acceptable representative or principal investigator or subinvestigator has failed to adhere to significant protocol

requirements (inclusion and exclusion criteria) and the patient was enrolled without prior sponsor approval, or when there is nonadherence to regulations or ICH Good Clinical Practice guidelines.

Protocol violations and deviations will be documented by the clinical monitor throughout the course of monitoring visits. Principal investigators or subinvestigators will be notified in writing by the monitor of violations and deviations.

5.7 Study Termination

The end of the study is defined as the date on which the last patient completes the last visit (includes follow-up visits).

Although Sobi has every intention of completing the study, Sobi reserves the right to discontinue the study at any time for clinical or administrative reasons.

5.8 Final Report

Whether the study is completed or prematurely terminated, the sponsor will ensure that the clinical study report is prepared and provided to the regulatory agencies as required by the applicable regulatory requirements. The sponsor will also ensure that the clinical study report meets the standards of the ICH harmonised tripartite guideline E3: Structure and content of clinical study reports.

The results of the assessments of all patients that have completed the 3-month visit and 200 patients that have completed the 12-months-corrected-age visit, including the Bayley assessment, will be presented in a clinical study report. A separate addendum to the report will be prepared when the remaining patients have completed the 12-month visit. The 24-months-corrected-age assessments, the health economic assessments, and any serious ADRs will be reported in a separate clinical study report when all patients have completed the study. Exploratory endpoints may be reported separately.

Where required by applicable regulatory requirements, the clinical study report will be approved and signed by the international coordinating investigator. The international coordinating investigator will be provided reasonable access to statistical tables, figures, and relevant reports and will have the opportunity to review the complete study results.

Upon completion of the study and reporting of 24-months-corrected-age data, the sponsor will provide the investigators with the full summary of the study results. The investigators are encouraged to share the summary results with the study patients' legally acceptable representatives, as appropriate.

6 Appendices

Appendix 1: Schedule of Events

Table 6–1 Schedule of Events until Discharge

Visit	Screening[b]	Treatment Period[a]				Discharge
		Baseline[b,c]	Daily	Weekly	End of Treatment	
Day	-7 to -1	1	2 to 28	8, 15, 22	29	Discharge
Informed consent	x					
Medical history	x					
Inclusion and exclusion criteria	x	x				
Randomization		x				
Demographic data	x					
Routine care laboratory test[d]	(x)	(x)	(x)		(x)	(x)
Vitamins A and D		x			x (± 3 days)	
Antibodies to rhBSSL		x			x (± 3 days)	
Fatty acids		x			x (± 3 days)	
Physical examination	x	x		x	x (± 3 days)	x
Body weight (g)[e]		x		x	x	x
Total body length (mm)		x		x	x	x
Head circumference (mm)		x		x	x	x
Body composition (Pea Pod®)[f]		x			x (± 3 days)	
Daily enteral feeding volume (mL) and feed type		x	x		x	
Calprotectin and microbiota fecal sample		x			x (± 3 days)	
Body temperature (°C)	x	x		x	x	x
Blood pressure and heart rate	x	x		x	x	x
Routine care electrocardiogram[g]	(x)	(x)	(x)		(x)	(x)
Concomitant medication	x	x	x		x	x
Administration of study drug[h]		x	x			
Parenteral nutrition type and volumes		x	x		x	
Routine care food fortification (type and amount)		x	x		x	
Gastrointestinal tolerability		x	x			
Adverse events[i]	x	x	x		x	x

Table 6–1 Schedule of Events until Discharge (Continued)

[a.] The addition of study drug to the food will start on Day 1 (Baseline), when the infant has reached a level of enteral feeding of at least 100 mL/kg/day and will continue for 4 weeks.

[b.] The screening and baseline visits can take place at the same time. All baseline assessments should be performed and documented in the electronic case report form prior to study drug administration on Day 1. The predose assessments on Day 1 will be the patient’s baseline values.

[c.] The baseline visit (Day 1) should occur on the day of randomization or on the day after randomization.

[d.] When a routine safety sample is collected during the treatment period, the following should, whenever possible, be determined in those samples: amylase, aminotransferases (alanine aminotransferase and aspartate aminotransferase), total bilirubin, sodium, and urea. If no routine samples are collected during Days 14 to 29, one sample must be collected for the analysis of amylase, aminotransferases, total bilirubin, sodium, and urea. Information regarding laboratory values judged by the investigator to be abnormal and clinically significant will be captured in the electronic case report form up to the 3-months visit. Information will also be captured until the values return to normal.

[e.] The patient’s body weight in grams will, as a minimum, be measured at Baseline (Day 1, before start of study drug administration), on Days 8, 15, 22, and 29, and on at least 2 other time points per week. Thereafter, body weight should be recorded at least weekly until discharge. Body weight measured at any other time point from randomization up to discharge should also be recorded in the electronic case report form.

[f.] Only at study centers with access to the necessary equipment.

[g.] Only recorded when available within routine care. No extra electrocardiograms should be taken during the study.

[h.] Administration of study drug will continue for 28 days, ie, until body weight is collected on Day 29.

[i.] All serious adverse events will be reported from the time the informed consent has been signed and up to the 12-months-corrected-age visit. Adverse events (including monitoring of infections, necrotizing enterocolitis, sepsis, intraventricular hemorrhage, retinopathy of prematurity, hearing impairment, bronchopulmonary dysplasia, and cholestasis) will be recorded from the start of treatment on Day 1 until the 3-months follow-up visit.

Table 6–2 Schedule of Events for Follow-up Visits

Visit	Follow-up						
	40 Weeks Post-menstrual Age[a]	Month 3	3 Months Corrected Age[a]	Month 6[b]	12 Months Corrected Age	Telephone contacts at 15, 18 and 21 Months Corrected Age[c]	24 Months Corrected Age[c]
Day	>49<br>±3 days	91<br>±7 days	>140<br>±7 days	183<br>±7 days	>415<br>±14 days	±1 month	±14 days
Informed consent					x[d]		
Routine care laboratory test[e]		(x)					
Antibodies to rhBSSL		x		x[f]	x[f]		
Physical examination		x			x		
Body weight (g)		x			x		x
Total body length (mm)		x			x		x[g]
Head circumference (mm)		x			x		x
Body composition (Pea Pod®)[h]	x		x				
Concomitant medication	x	x					
Bayley-III (cognition, language and motor)					x		x
Bayley-III (social-emotional, adaptive behavior)							x
Child Behavior Checklist							x
Neurodevelopment disability composite							x
Confounders of neurodevelopment					x[i]		x
Adverse events[j]	x	x	x[k]	x[k]	x[k]	x[l]	x[l]
Health economic assessments					x[i]	x	x

Table 6–2 Schedule of Events for Follow-up Visits (Continued)

a. The 40 weeks postmenstrual age and the 3-months-corrected-age visits will only occur for patients with body composition assessments during the treatment period.

b. The 6-months visit will only occur for patients where it is required that an antibody sample is collected.

c. Will only occur if informed consent for the scheduled assessments has been provided.

d. For the 24-months-corrected-age assessments, confounders of neurodevelopment, health economic assessments and any serious adverse drug reactions. Can be done at a later time point for patients that have conducted the 12-months-corrected-age visit before the implementation of amendment 2.

e. Information regarding laboratory values judged by the investigator to be abnormal and clinically significant will be captured in the electronic caser report form up to the 3-months visit. Information will also be captured until the values return to normal.

f. Once cut-off level for a positive result has been defined, the sample is only collected for patients with a positive result at previous visit.

g. Measured as height (cm) at 24 months corrected age.

h. Only at study centers with access to the necessary equipment. During the follow-up period, Pea Pod measurements will be conducted at 40 weeks postmenstrual age and 3 months corrected age for all patients who have body composition assessments during the treatment period.

i. As soon as informed consent has been provided (earliest at the 12-months-corrected-age visit).

j. All serious adverse events will be reported from the time the informed consent has been signed and up to the 12-months-corrected-age visit. Adverse events (including monitoring of infections, necrotizing enterocolitis, sepsis, intraventricular hemorrhage, retinopathy of prematurity, hearing impairment, bronchopulmonary dysplasia, and cholestasis) will be recorded from the start of treatment on Day 1 until the 3-month follow-up visit. Serious adverse drug reactions will be reported up to 24 months corrected age.

k. The investigator will ask the patient's legally acceptable representative about possible ongoing or previous (since last visit) events that fulfill the serious adverse event criteria.

l. The patient's legally acceptable representative will be asked about any ongoing or previous (since last contact or visit) events that fulfills the serious adverse event criteria. The investigator will then judge if a causal relationship between the serious adverse event and the study drug is suspected i.e., if the serious adverse event is a serious adverse drug reaction.

Appendix 2: Protocol Amendment 1.0

Text with a **bold** font has been added and text with a ~~strikethrough~~ font has been deleted.

Version	Date	Changes
Version 1.0	10 February 2011	Original Protocol
Version 2.0 Amendment 1.0	27 May 2011	**Synopsis – Objectives**<br>Exploratory objectives of this study are<br>• ~~To assess the effect of rhBSSL treatment on levels of vitamin D~~<br>• To assess the effect of rhBSSL treatment on fatty acid levels (other than DHA and AA)<br>• To assess the effect of rhBSSL on the body composition<br>• To assess the effect of rhBSSL treatment on the fecal calprotectin and microbiota contents
		**Synopsis – Exclusion criteria**<br>10. Prior or ~~concomitant~~**current** treatment with corticosteroids, except hydrocortisone.
		**Synopsis – Endpoints**<br>The study has the following secondary safety endpoints:<br>• Adverse events<br>• Physical examination<br>• Bayley Scale of Infant and Toddler Development III (cognitive)<br>• Bayley Scale of Infant and Toddler Development III (language)<br>• Bayley Scale of Infant and Toddler Development III (motor)<br>• Vomiting (frequency and volume)<br>• Vital signs (heart rate, blood pressure, body temperature)<br>• ~~Routine~~ Laboratory variables ~~including~~ amylase, bilirubin, aminotransferases, **sodium,** and urea<br>• Levels of vitamin**s** A **and D**<br>• Levels of rhBSSL antibodies<br>The study has the following exploratory endpoints:<br>• ~~Levels of vitamin D~~<br>• Levels of fatty acids, other than DHA and AA<br>• Percent body fat<br>• Fecal calprotectin concentration<br>• Fecal microbiota
		**Synopsis – Statistical Methods**<br>Time to readiness for discharge ~~and time from start of treatment to 150 mL/kg/day of enteral feeding~~ will be analyzed using Cox proportional hazards models.

2.2 Exploratory Objectives

Exploratory objectives of this study are

- ~~To assess the effect of rhBSSL treatment on levels of vitamin D~~
- To assess the effect of rhBSSL treatment on fatty acid levels (other than DHA and AA)
- To assess the effect of rhBSSL on the body composition
- To assess the effect of rhBSSL treatment on the fecal calprotectin and microbiota contents

2.3.2 Secondary Endpoints

The study has the following secondary safety endpoints:

- Adverse events (AEs)
- Physical examination
- Bayley Scale of Infant and Toddler Development III (cognitive)
- Bayley Scale of Infant and Toddler Development III (language)
- Bayley Scale of Infant and Toddler Development III (motor)
- Vomiting (frequency and volume)
- Vital signs (heart rate, blood pressure, body temperature)
- ~~Routine~~ Laboratory variables ~~including~~ amylase, bilirubin, aminotransferases, **sodium,** and urea
- Levels of vitamin**s** A **and D**
- Levels of rhBSSL antibodies

2.3.3 Exploratory Endpoints

The study has the following exploratory endpoints:

- ~~Levels of vitamin D~~
- Levels of fatty acids, other than DHA and AA
- Percent body fat
- Fecal calprotectin concentration
- Fecal microbiota

3.2.2 Exclusion Criteria

10. Prior or ~~concomitant~~**current** treatment with corticosteroids, except hydrocortisone.

3.3.1 Reasons for Withdrawal

The investigator may withdraw a patient from the study treatment for the following reasons:

1. Patient experiences serious or intolerable (as determined by the investigator) AEs;

~~2. Patient requires a medication or treatment that is prohibited by the protocol;~~ or

~~3.~~**2.** Patient's legally acceptable representative requests an early discontinuation for any reason.

The investigator ~~will~~**may** also withdraw a patient **from study treatment based on his or her own judgment** upon the request of Sobi**,** or if Sobi terminates the study. Upon occurrence of a serious or intolerable AE, the principal investigator will confer with PPD. If a patient is discontinued because of an AE, the event will be followed up until it is resolved or the condition is stable.

3.4.2 Demography Assessments

At randomization the following should be recorded:

- Body weight
- Total body length
- Head circumference
- Sex
- Race
- Actual birth date
- Expected birth date
- Body weight at birth

~~If the method used to obtain these data is different from that required for the measurements taken during this study, this should be recorded in the eCRF.~~

3.4.4.1.2 Eliciting and Documenting Adverse Events

The investigator is responsible for reporting all AEs that are observed or reported during the study, regardless of their relationship to study drug or their clinical significance.

The investigator will **report and** monitor ~~the patient for~~ **AEs of special interest, ie,** infections, necrotizing enterocolitis, sepsis, intraventricular hemorrhage, retinopathy of prematurity, hearing impairment, bronchopulmonary dysplasia, and cholestasis.

Adverse events will be recorded from ~~the start of treatment on Day 1~~**baseline** until the 3-month follow-up visit. **Serious AEs will be reported from the time the informed consent has been signed.** Any AEs present at the 3-month visit should be followed until resolved or the condition is stable. At the 6-month (if applicable) and at the 12 months' corrected age follow-up visits, the investigator will ask the patient's legally acceptable representative about any ongoing or previous (since last visit) events that fulfill the SAE criteria.

3.4.4.1.3 Reporting Adverse Events

~~All~~ AEs reported or observed ~~during the study~~ will be recorded on the AE page of the eCRF **as described in the eCRF completion guidelines**. Information collected will include date and time of onset, investigator-specified assessment of severity and relationship to study drug, date and time of resolution of the event, seriousness, as well as any required treatment or evaluations, and outcome. Adverse events resulting from concurrent illnesses, reactions to concurrent illnesses, reactions to concurrent medications, or progression of disease states must also be reported. All AEs will be followed to adequate resolution. The Medical Dictionary for Regulatory Activities (MedDRA) will be used to code all AEs (Section 4.10).

3.4.4.1.3 Reporting Adverse Events

Any AE considered serious by the investigator or subinvestigator or which meets SAE criteria (Section 3.4.4.1.1) must be reported to PPD Pharmacovigilance (PVG) Department within 24 hours from the time study center personnel first learn about the event. The following contact information should be used for SAE reporting **(Fax line for sending the SAE notification and the hotline and e-mail for questions)**:

~~Medical Affairs/Pharmacovigilance~~

~~PPD Granta Park~~
~~Great Abington,~~
~~Cambridge CB21 6GQ~~
~~United Kingdom~~
~~PPD PVG Hotline: +44 1223 374240~~
Fax line for sending the SAE notification: PPD PVG Fax line: +44 1223 374102

The hotline and e-mail for questions:
PPD PVG Hotline: +44 1223 374240
PPD PVG E-mail: emeaasiasafetycentral.sm@ppdi.com

PPD PVG address:
Medical Affairs/Pharmacovigilance, PPD Granta Park, Great Abington, Cambridge CB21 6GQ, United Kingdom

3.4.4.1.5 Assessment of Causality

"Not related" and "unlikely" will be included in the category "unrelated" (ie, not having a reasonable suspected causal relationship to the study drug) in sponsor reports. "Possible", "probable" and "definite" will be included in the category "related" (ie, having a reasonable causal relationship to the study drug) in sponsor reports.

3.4.4.2 Physical Examination

Physical examination will be performed at Screening, Baseline, Days 8, 15, 22, and 29, at discharge, and at the follow-up visit at 3 months and the 12 months' corrected age follow-up visit. Any clinically ~~relevant~~**significant** changes from Baseline noted during the physical examinations should be reported as AEs.

3.4.4.3 Gastrointestinal Tolerability

Tolerability assessments will be performed at Baseline **and then** daily until the end of treatment~~, and weekly until the patient is discharged~~. The tolerability assessments will consist of recording incidences and estimated volumes of vomiting. Vomiting should also be reported as an AE ~~where it meets the criterion~~**if considered clinically significant**.

3.4.4.3 Laboratory Safety Analysis

Routine hematology and chemistry assessments will be conducted at the local laboratories according to each hospital's standard procedures. Only a limited number of extra blood samples will be taken from this vulnerable population for standard laboratory safety assessment. When a routine safety sample is collected, the following should whenever possible be determined in those samples: amylase, aminotransferases (alanine aminotransferase and aspartate aminotransferase), total bilirubin, **sodium,** and urea. Of these, amylase, aminotransferases, and total bilirubin have the highest priority.

Blood samples will be collected on Day 29 for serum (or plasma) amylase, aminotransferases, total bilirubin, **sodium,** and urea, unless those have been determined in at least 1 blood sample collected following a minimum of 2 weeks of treatment with study drug. These analyses will be performed by the local laboratory.

3.4.4.7.1 Vitamins A and D

A blood sample for the analysis of vitamin A and vitamin D, ie, D 25(OH)D_2 and 25(OH)D_3, **including inactive epimer forms,** in serum will be collected at Baseline and on Day 29. Section 3.4.6 provides further details of sampling. ~~The determination of Vitamin D is considered an exploratory assessment.~~ ~~Current~~**Previously available** methods for determination of vitamin D use serum volumes that are unacceptably high for the preterm infants~~ in this study~~. Therefore, an assay that can determine both vitamin**s** A and ~~vitamin~~ D in the same sample ~~is currently being~~ **has recently been** developed at Sobi. This assay also includes determination of free cholesterol, since the levels of the lipophilic vitamins vary with the levels of serum lipids. The ~~aim of the~~**new** assay ~~development is to increase~~**has an increased** sensitivity relative to ~~the currently~~**previously** available methods, and thereby reduce**s** the required blood volume.

3.4.4.7.2 rhBSSL Antibodies

A blood sample will be collected at Baseline, on Day 29, and at the 3-month follow-up visit for the determination of rhBSSL antibodies in serum. A further sample will be taken at the 6-month follow-up visit~~if antibodies were detected at 3 months~~. **Once the cut-off level for a positive antibody result has been defined (based on baseline data from approximately the first 50 patients), the 6-month sample will only be taken for those patients who have a positive result for the 3-month sample.** If ~~antibodies are detected~~**the result is positive** at 6 months, a further sample will be collected at the 12 months' corrected age follow-up visit. Section 3.4.6 provides further details of sampling.

~~3.4.5.1 Vitamin D~~

~~See Section 3.4.4.7.1~~

3.4.5.3 Body Composition

Body composition (percent fat mass), will be determined by air displacement plethysmography at study centers with access to the necessary equipment (Pea Pod®~~; Life Measurement, Inc, Concord, California, United States~~). Measurements will be performed at Baseline, Days 15 and 29, at 3 months, and at the 12 months' corrected age visit (Table 6–1). Measurements should be taken as near to the same time each day as is possible.

3.4.6 Sample Collections

Table 3-1 has been updated to clarify the blood volumes and to include sodium in the safety assessments but the table is not reproduced here

Footnote b: ~~Sample to be taken if antibodies are detected at previous visit~~**Once cut-off level for a positive result has been defined, the sample is only collected for patients with a positive result at previous visit**.

3.6.5.3 Infant Formula Requirements

Each study center will select one infant formula that all formula fed patients at that study center will be fed. They will remain on the same infant formula throughout the treatment period, unless medically contraindicated. The exact brand name of the infant formula used should be recorded in the eCRF to enable the composition to be derived.

The range of protein, carbohydrate, **and** lipid~~, and energy~~ content for the infant formulas allowed for use in this study are

Protein: 2.8 to 4.1 g/100 kcal

Carbohydrate: ~~10~~**9**.5 to 12.0 g/100 kcal

Lipids: 4.4 to 6.0 g/100 kcal

~~Energy: 100 to 135 kcal/kg/day~~

In addition, all infant formulas to be used in the study must contain AA and DHA. The infant formula must contain less than **or equal to** 40% of medium-chain TGs.

3.6.5.4 Pasteurized Breast Milk Requirements

There are no special requirements with regard to the source or quality of the PBM. The milk may be the mother's own or come from a milk bank (donor milk). ~~When available from the milk bank, the composition~~**Information about the predominant milk type will be recorded in the eCRF. The composition of the milk** (fat, protein**,** and lactose) ~~of the milk batch~~should be recorded **when available**. ~~Where this is unavailable, a 3-mL sample is collected for analysis of the milk composition. Details are provided in a separate laboratory manual.~~

Fortification of the milk should be done according to a predefined study center-specific schedule. Preferably, one fortifier should be used at a study center, and the amounts added should be fixed, ie, the same concentration added to all PBM. Fortification must not be individualized based on body weight. All use of fortifiers, including lipid emulsions, should be recorded in the eCRF.

The availability of PBM varies between NICUs, and thus the time period during which an infant is given only PBM before switching to infant formula will vary. In this study, the time of switching to infant formula must not be based on body weight. Instead, each study center using PBM in this study should, before the first infant is randomized, declare which of the following alternatives will be followed for the infants in the study:

1. PBM only for 4 weeks
2. PBM only for 2 weeks **followed by infant formula for 2 weeks**

The date when infant formula is first introduced should be recorded in the eCRF, as well as the volumes of PBM and, if applicable, infant formula.

3.6.5.4 Pasteurized Breast Milk Requirements

No fresh breast milk should be used during the 29 days of treatment. Any deviation from this rule must be noted in the eCRF. However, for ethical reasons, breast feeding cannot be prohibited. During the treatment period, the infants may be put to the breast, and the consumption of small volumes is allowed. **If this is the case the date when fresh breast milk was first consumed will be recorded in the eCRF.**

3.6.7 Breaking the Blind

The PPD medical monitor will be responsible for maintaining the blind throughout the study. If a patient becomes seriously ill, the blind will be broken only if knowledge of the administered study drug will affect treatment options available to the patient.

At the initiation of the study, study centers will be instructed on the method for breaking the blind. This will be an electronic process via the IVRS, which is fully documented in the IVRS manual. Blinding codes should only be broken in emergency situations for reasons of patient safety. The investigator should contact PPD, who will contact the sponsor before breaking the blind. In an emergency when there is insufficient time for the investigator to contact PPD or the sponsor, the investigator is permitted to break the blind without contacting PPD or the sponsor. The sponsor and PPD must be notified as soon as possible after the blind break. ~~When the blinding code is broken, it must be fully documented and complete details entered in the eCRF and the patient's medical record.~~ **Only the IVRS user who is logged in during the emergency code break session will receive an unmasked copy of the notification in real time. All other users (clinical team and sponsor team) will receive a blinded real-time notification informing them of the code break.**

3.6.9 Prior, Concomitant, and Subsequent Therapy

Other therapy considered necessary for the patient's welfare may be given at the discretion of the investigator. All such therapy must be recorded in the eCRF. ~~The concomitant administration of corticosteroids (except hydrocortisone) is prohibited.~~ The patient is not allowed to participate concurrently in another clinical intervention study and as such may not use any drug or treatment concurrently under investigation.

Preterm infants often experience complications that need therapeutic intervention. This can in most cases be accepted as long as the medication does not interfere with feeding.

~~Prior medication, from birth until start of treatment, and a~~**A**ll use of concomitant and subsequent medications, from **the** start of **the** ~~treatment~~**study** up to the 3-month follow-up visit, will be recorded in the patient's eCRF (Table 6–1). This will include all prescription drugs, herbal products, vitamins, minerals, and over the counter medications. Any changes in concomitant medications also will be recorded in the patient's eCRF.

3.7.3 Safety Endpoints

The study has the following secondary safety endpoints to meet the objec**tive** "To compare the safety and tolerability of rhBSSL treatment in preterm infants with that of placebo treatment after oral administration in infant formula or PBM":

- AEs
- Physical examination
- Bayley Scale of Infant and Toddler Development III (cognitive)
- Bayley Scale of Infant and Toddler Development III (language)
- Bayley Scale of Infant and Toddler Development III (motor)
- Vomiting (frequency and volume)
- Vital signs (heart rate, blood pressure, body temperature)
- Levels of amylase, bilirubin, aminotransferases, **sodium,** and urea
- Levels of vitamin**s** A **and D**
- Levels of rhBSSL antibodies

3.7.4 Exploratory Endpoints

The study has the following exploratory endpoints:

- ~~Levels of vitamin D~~
- Levels of fatty acids, other than DHA and AA
- Percent body fat
- Fecal calprotectin concentration
- Fecal microbiota

3.8.1 Primary Efficacy Analysis

In order to calculate growth velocity, the natural log-transformed value of the baseline and all ~~each~~ postbaseline weight assessment for each patient will be calculated. A linear regression model will then be fitted for each patient with a response variable of log(weight) and a predictor variable of time. Growth velocity for each patient will be estimated as the slope arising from the regression model, and will need to be multiplied by 1000 for conversion into the desired unit of grams per kilogram per day. The weight over time for each treatment group will be illustrated graphically and if the log-transformed data clearly deviate from the linearity assumption, a sensitivity analysis will be performed using untransformed data.

5.3 Central Laboratories

rhBSSL Antibodies **and Vitamins A and D**
York Bioanalytical Solutions Ltd
Cedar House
Northminster Business Park
Upper Poppleton
York YO26 6QR
United Kingdom

Fatty Acids
~~To be determined~~

Nutrition Research Program
Child and Family Research Institute
Room 171B
950 West28th Avenue
Vancouver
British Columbia V5Z4H4
Canada

~~Vitamin A and D~~
~~To be determined.~~

Fecal Calprotectin and Microbiota
~~To be determined.~~

PPD Central Laboratory
2 Tesseneer Drive
Highland Heights, KY 41076
USA

~~Milk Composition Analysis~~
~~To be determined.~~

6 Appendices

Appendix 1 Table 6-1 Schedule of Events

Table 6–1 has been updated to reflect all the changes made to the protocol as part of this amendment.

Footnote d: The Month 6 visit will only occur for patients ~~who require~~**where it is required that** an antibody sample ~~to be~~**is** collected.

~~Study treatment may stop before discharge.~~

Footnote e: When a routine safety sample is collected, the following should whenever possible be determined in those samples: amylase, aminotransferases (alanine aminotransferase and aspartate aminotransferase), total bilirubin, **sodium,** and urea. If no routine samples are collected during Days 14 to 29 one sample must be collected for the analysis of amylase, aminotransferases, total bilirubin, **sodium,** and urea.

Footnote f: ~~Sample will be taken only if antibodies were detected at the previous visit.~~ **Once cut-off level for a positive result has been defined, the sample is only collected for patients with a positive result at previous visit.**

Footnote l: **Serious AEs will be reported from the time the informed consent has been signed. Adverse events (**including monitoring of infections, necrotizing enterocolitis, sepsis, intraventricular hemorrhage, retinopathy of prematurity, hearing impairment, bronchopulmonary dysplasia, and cholestasis**) will be recorded from baseline until the 3-month follow-up visit.**

	**7 Reference List**
	Hawcutt DB, Rose AC, Fuerst-Recktenwald S, et al. Points to consider when planning the collection of blood or tissue samples in clinical trials of investigational medicinal products in children, infants and neonates. Guide to paediatric drug development and clinical research. Basel, Karger; 2010. 97-11~~9~~**0**.

Appendix 3: Administrative Change 1.0

Text with a **bold** font has been added and text with a ~~strikethrough~~ font has been deleted.

Version	Date	Changes
Version 1.0	10 February 2011	Version 1.0
Version 2.0<br>Amendment 1.0	27 May 2011	See Appendix 2
Version 3.0<br>Administrative<br>Change 1.0	14 May 2012	**Title Page and Protocol Approval Signature Page**<br><br>**Project Manager:** ~~Matthias Oleszewski~~<br>~~Senior Project Manager~~<br>~~PPD Germany GmbH Stephanienstraße 55~~<br>~~76133 Karlsruhe, Germany~~<br>~~Telephone: 49 721 9184 0~~<br><br>**Tracy Roe**<br>**Associate Director, Project Management**<br>**PPD Inc**<br>**Franklin House, Kings Worthy**<br>**Winchester, Hampshire, UK**<br>**Telephone: 44 7944 073 138**
		**Synopsis – Exclusion Criteria and 3.2.2 Exclusion Criteria**<br><br>7. Clinical evidence of sepsis (including low or high white blood cell count and/or low platelet count and bacteriologically proven evidence of systemic infection). This should be based on the investigator's opinion and available local laboratory reference ranges.<br>**8. Systemic anti-infective treatment within 48 hours prior to randomization, other than prophylactic treatment** ~~Patients should not have received antibiotics in preceding 48 hours except where these are being administered for prophylaxis as per routine unit protocols~~ (eg, antifungal prophylaxis with fluconazole) **as per local clinical practice**.<br><br>**Note that exclusion criteria following these were accordingly renumbered*
		**Synopsis – Study Design and 3.1 Study Design**<br><br>The addition of study drug to the food will start on Day 1, which will be as soon as possible after randomization (either on the day of randomization or the day after). The predose assessments on Day 1 (the day the first dose of study drug is administered) will be the patient's baseline values. The administration of study drug will continue for 4 weeks. Follow-up visits will occur at 3 months after the first dose of study drug and at 12 months corrected age. There will be an additional follow-up visit at 6 months after the first dose of study drug if rhBSSL antibodies are found at 3 months. **Patients who have body composition assessments during the treatment period will also return for follow-up assessments at 40 weeks postmenstrual age and 3 months corrected age.**
		**Figure 3-1 Study Schematic**<br><br>~~4 weeks~~ **End of Treatment (Day 29)**

Version	Date	Changes
		**Note: Patients who have body composition assessments during the treatment period will also return for follow-up assessments at 40 weeks postmenstrual age and 3 months corrected age.**
		**3.4.2 Demography Assessments**<br><br>At ~~randomization~~ **Baseline** the following should be recorded:
		**3.4.3.1 Body Weight**<br><br>~~Available body weight data will be retrospectively recorded in the eCRF from birth up to randomization.~~ **Body weight at birth and the lowest measured body weight should be retrospectively recorded in the eCRF.** The patient's body weight in grams will, as a minimum, be measured at Baseline (Day 1, before start of study drug administration), on Days 8, 15, 22, and 29, and on at least 2 other time points per week. Thereafter, body weight should be recorded at least weekly until discharge, and at each scheduled follow-up visit (Table 6–1). Body weight measured at any other time point from randomization up to discharge should also be recorded in the eCRF**, but not more than 1 record per day.**<br><br>**During the treatment period,** the body weight should be measured according to standard unit procedures, as near to the same time each day as is possible, and using the same scale (provided for the study), with graduation of 2 g, each time. The weight of catheters, tubes, and other items that cannot be removed should be subtracted from the total measured weight. **Preferably the same equipment should also be used for assessments made after the treatment period, where possible.**
		**3.4.3.2 Total Body Length**<br><br>**During the treatment period,** the patient's total length (mm) will be measured from the crown to the heel using a preterm infant length board **provided for the study** and recorded in the eCRF. **Preferably and where possible the same equipment should also be used for assessments made after the treatment period.** Total body length should be measured according to separate detailed instructions. This measurement should be made at Baseline **(Day 1)**, Days 8, 15, 22, and 29, at discharge, and at each scheduled follow-up visit (Table 6–1). Measurements should be taken as near to the same time each day as is possible.
		**3.4.3.3 Head Circumference**<br><br>**During the treatment period,** the circumference of the patient's head (mm) will be measured, using a nonstretch measuring tape provided for this study, and recorded in the eCRF. **Preferably and where possible the same equipment should also be used for assessments made after the treatment period.** The measuring of head circumference should be made according to separate detailed instructions. This measurement should be made at Baseline **(Day 1)**, Days 8, 15, 22, and 29, at discharge, and at each scheduled follow-up visit (Table 6–1). Measurements should be taken as near to the same time each day as is possible.
		**3.4.3.5 Docosahexaenoic Acid (DHA) and Arachidonic Acid (AA)**<br><br>Fatty acids, including DHA and AA, will be determined in either serum or plasma at Baseline **(Day 1)** and at ~~4 weeks (~~**the** end of treatment **(Day 29)**. Fatty acids other than DHA and AA are considered exploratory assessments.

Version	Date	Changes
		**3.4.4.1.1 Definitions**<br><br>**Serious Adverse Event**<br>An SAE is defined as any event that<br>• results in death,<br>• is immediately life threatening (includes events that put patients at risk of death at the time of the event but not events that may have caused patient death if more severe),<br>• requires inpatient hospitalization or prolongation of existing hospitalization,<br>• results in persistent or significant disability or incapacity,<br>• is a congenital anomaly or birth defect.<br>Important medical events that may not result in death, be life threatening, or require hospitalization may be considered an SAE when, based upon appropriate medical judgment, they may jeopardize the patient and may require medical or surgical intervention to prevent one of the outcomes listed in this definition.<br>**However, preplanned/administrative hospitalization will not be considered as an SAE, for example:**<br>• **Hospitalization for a procedure that is planned prior to signing the informed consent for enrolling in the study, without a worsening of a pre-existing disease. The procedure must be documented in the source documents and in the eCRF.**<br>• **Hospitalization or prolongation of hospitalization for administrative reasons such as technical, practical, or social reasons, in absence of an AE (eg, preplanned hospitalization for vaccinations according to a vaccination program or hospital general policies). This must also be documented in the source documents and in the eCRF.**<br>**Please note that hospitalization or prolonged hospitalization for a complication to the preplanned/administrative procedure remains a reportable SAE.**
		**3.4.4.1.2 Eliciting and Documenting Adverse Events**<br><br>The investigator is responsible for reporting all AEs that are observed or reported during the study, regardless of their relationship to study drug or their clinical significance.<br>The investigator will report and monitor AEs of special interest, ie, infections, necrotizing enterocolitis, sepsis, intraventricular hemorrhage, retinopathy of prematurity, hearing impairment, bronchopulmonary dysplasia, and cholestasis. **These AEs will be handled as AEs, or if meeting the serious criteria, as SAEs. The AEs of special interest will be discussed in a separate section of the study report.**<br>Adverse events: **AEs** will be recorded from ~~baseline~~ **start of treatment on Day 1** until the 3-month follow-up visit.<br>**Adverse events present at the 3-month visit should be followed up at the patient's next visit (6-month [if applicable] or 12 months). Adverse events that have not resolved or stabilized at the 12 months' corrected age visit should be followed up until satisfactory resolution or until the principal investigator or subinvestigator deems the event to be chronic or not**

Version	Date	Changes
		**clinically significant or the patient's condition to be stable, but without further recordings in the eCRF.**<br><br>Serious AEs: **SAEs** will be reported from the time the informed consent has been signed **until the patient's last visit**. ~~Any AEs present at the 3-month visit should be followed until resolved or the condition is stable. At the 6-month (if applicable) and at the 12 months' corrected age follow-up visits,~~ **At every follow-up visit,** the investigator will ask the patient's legally acceptable representative about any ongoing or previous (since last visit) events that fulfill the SAE criteria.<br><br>**Any SAEs present at the 12-months-corrected-age visit should be followed up until satisfactory resolution or until the principal investigator or subinvestigator deems the event to be chronic or not clinically significant or the patient's condition to be stable, but without further recordings in the eCRF. Additional information received regarding patients with ongoing SAEs after 12 months should be reported via fax notification as described in Section 3.4.4.1.3.**
		**3.4.4.1.3 Reporting Adverse Events**<br><br>Any medical condition that is present at the time that the patient is screened but does not deteriorate should not be reported as an AE. However, if it deteriorates at any time during the study, it should be recorded as an AE.<br><br>**A hospitalization for administrative reasons in the absence of an AE is not considered to be an SAE. If a patient is hospitalized for a procedure that was planned prior to when the informed consent was obtained, it is not considered an SAE. For further information, see Section 3.4.4.1.1.**
		**3.4.4.1.4 Assessment of Severity**<br><br>~~Changes in the severity of an AE will be documented to allow an assessment of the duration of the event at each level of severity to be performed. Adverse events characterized as intermittent require documentation of onset and duration of each episode.~~ **Only one intensity of the AE will be recorded. If the intensity of the AE changes, the maximum intensity of the event will be recorded.**
		**3.4.4.1.6 Follow-up of Patients With Reported Adverse Events**<br><br>All AEs (**including SAEs) captured in accordance with Section 3.4.4.1.2** must be ~~reported in detail on the appropriate eCRF and~~ followed to satisfactory resolution or until the principal investigator or subinvestigator deems the event to be chronic or not clinically significant or the patient**'s** condition to be stable, **but without further recordings into the eCRF after the 12-months-corrected-age visit. Follow-up information from SAEs that are ongoing at the 12-months-corrected-age visit will be reported via the fax notification as described in Section 3.4.4.1.3.**<br><br>**Sobi retains the right to request additional information for any patients with ongoing AE(s)/SAE(s) at the end of the study, if judged necessary.**
		**3.4.4.6 Bayley Scales of Infant and Toddler Development**<br><br>The ~~investigator will assess the patient against the~~ Bayley Scales of Infant and Toddler Development, third edition, **will be used to assess the patient** at 12 months corrected age. The study will assess the cognitive, motor, and language items from the Bayley Scales. The results of the assessments should

Version	Date	Changes
		be recorded in the eCRF.
		**3.4.4.7 Laboratory Safety Analyses**<br><br>Blood samples will be collected ~~on~~ **at the end of treatment (**Day 29**)** for serum (or plasma) amylase, aminotransferases, total bilirubin, sodium, and urea, unless those have been determined in at least 1 blood sample collected following a minimum of 2 weeks of treatment with study drug. These analyses will be performed by the local laboratory.
		**3.4.4.7.1 Vitamins A and D**<br><br>A blood sample for the analysis of vitamin A and vitamin D, ie, D 25(OH)D2 and 25(OH)D3, including inactive epimer forms, in serum will be collected at Baseline **(Day 1)** and ~~on~~ **at the end of treatment (**Day 29**)**.
		**3.4.4.7.2 rhBSSL Antibodies**<br><br>A blood sample will be collected at Baseline **(Day 1)**, ~~on~~ **at the end of treatment (**Day 29**)**, and at the 3-month follow-up visit for the determination of rhBSSL antibodies in serum.
		**3.4.5.2 Body Composition**<br><br>Body composition (percent fat mass), will be determined by air displacement plethysmography at study centers with access to the necessary equipment (Pea Pod®). Measurements will be performed at Baseline **(Day 1)**, ~~Days 15 and~~ **at the end of treatment (**Day 29**)**, at ~~3 months, and at the 12 months'~~ **40 weeks postmenstrual age, and 3 months corrected age, with the 2 latter** visit**s scheduled only for patients with body composition assessments during the treatment period** (**Table 6–1**). ~~Measurements should be taken as near to the same time each day as is possible.~~
		**3.4.5.3 Calprotectin and Microbiota**<br><br>A fecal sample will be collected at Baseline **(Day 1)** and ~~on~~ **at the end of treatment (**Day 29**)**. Detailed instructions regarding sampling and sample handling and shipping will be given in a separate laboratory manual. The samples will be analyzed with respect to calprotectin and bacterial contents.
		**3.6.4.1 Study Drug Packaging and Storage**<br><br>The study drug, rhBSSL **and placebo**, will be stored in single-dose glass vials fitted with rubber stopper and sealed with flip tear-off aluminum caps.<br><br>The vials are packed together with sterile water for reconstitution of the powder**.** ~~and an administration dispenser. The dispenser is a~~ 1-mL oral/enteral syringe**s** intended for administration of the reconstituted medicinal product to the food **and syringe tip caps will be provided**. The syringe**s** and ~~stopper~~ **tip caps** will be provided sterile and individually packed for single use.<br><br>Recombinant human BSSL drug substance is manufactured by WuXi AppTec Inc, (Philadelphia, Pennsylvania, United States) and drug product and matching placebo are manufactured by Patheon Italia SpA (Monza, Italy). PPD Ireland is responsible for storage and distribution in Europe and the PPD depot in Russia is responsible for storage and distribution in Russia.<br><br>The study drug must be stored refrigerated at 2°C to 8°C at the study center in a secure area. Once the study drug has been mixed with the food it **is stable for up to 30 hours if kept** ~~can be stored~~ refrigerated at 2°C to 8°C in a secure

Version	Date	Changes
		location ~~for up to 24 hours~~. **However, instructions for use for the specific formula and the unit's normal procedure must be considered when maximum length of storage is determined if it is shorter than 30 hours.**
		**3.6.4.2 Preparation of Study Drug and Food**<br><br>The pharmacist or delegate will mix the study drug with 1 mL of sterile water ~~and if the mixing process is performed in the pharmacy,~~ **If the reconstitution of study drug and the mixing of reconstituted study drug with the food is not done at the same location,** the syringe ~~will~~ **can** be transported with the ~~stopper~~ **tip cap** in place ~~to the NICU ready for mixing with the food~~. Each vial of study drug will be mixed with 100 mL of PBM or infant formula.<br><br>Each feeding should be drawn from the main food bottle, heated ~~up~~ (not above 37°C) **or given at room temperature**, and the infant fed according to the unit's normal procedure. The main food bottle should not be heated~~,~~ **and** should remain refrigerated~~, and can be used for up to 24 hours~~.
		**3.6.4.3 Study Drug Accountability**<br><br>The investigator will maintain accurate records of receipt of all study drug, including dates of receipt. In addition, accurate records will be kept regarding when and how much study drug is dispensed (ie, added to food) and used by each patient in the study. The amount of drug actually administered to the patients will be calculated from the food records (Section 3.6.5.1). All used study drug vials should be stored in a secure area for drug accountability (room temperature). At the completion of the study all **remaining** study drug will be reconciled and returned, or destroyed locally according to applicable regulations.
		**3.6.5.2 Daily Volume of Parenteral Nutrition**<br><br>The total daily volume (mL) of parenterally administered nutrition received by the patient **during the treatment period** will be recorded in the eCRF ~~every day during the treatment period~~ (Table 6–1). **The product name and volume of any** ~~Details of the~~ parenterally administered nutrition **containing a fat emulsion**~~, including the name of the product and its nutritional content,~~ should **also** be recorded.
		**3.7.2 Secondary Efficacy Endpoints**<br>The following secondary efficacy endpoints are evaluated to support the objective "To determine the effect of rhBSSL treatment in shortening the time of hospital stay":<br>• Time to readiness for discharge. This endpoint will be defined as the time from Baseline when all of the following criteria have been fulfilled: **a sustained weight gain,** ability to maintain normal body temperature, ability to suckle feed, and ability to maintain stable cardiorespiratory function.<br>• Time to discharge
		**4.13 Publications**<br><br>Sobi will ~~fulfill its commitment to publicly disclose the results of studies through posting the results of this study on ClinicalStudyResults.org. This study will be registered on~~ **publically register this study through posting the protocol and results of this study at** www.clinicaltrials.gov.

Version	Date	Changes
		The results of this study will be published within 1 year after the first regulatory approval of the product. If drug development is discontinued before approval, the results will be published within 1 year after such discontinuation.<br>~~The posting on ClinicalStudyResults.org will include the following elements:~~<br>~~• Protocol title, study phase, and indication~~<br>~~• Link to approved product labeling, if applicable~~<br>~~• Synopsis of study results~~<br>~~• Citations of known study publications~~<br>~~• Legal disclaimer~~<br>~~The study results synopsis posted on ClinicalStudyResults.org uses the format established by the guideline ICH E3: Structure and Content of Clinical Study Reports.~~<br>~~If posting of study results to ClinicalStudyResults.org jeopardizes a planned publication of the study results, a pending full publication notice is substituted for the synopsis until the study results publication has been issued or 2 years have elapsed, whichever occurs first.~~
		**Appendix 1 Table 6-1 Schedule of Events**<br>Heading: ~~Week 4~~ **End of Treatment**<br>Body composition time points were updated to reflect the changes per Section 3.4.5.2, which includes adding 2 new visits to the table (ie, 40 weeks postmenstrual age and 3 months corrected age).<br>Footnote i: ~~Day 15 only~~ **During the follow-up period, Pea Pod measurements will be conducted at 40 weeks postmenstrual age and 3 months corrected age for all patients who have body composition assessments during the treatment period.**

Appendix 4: Protocol Amendment 2.0

Text with a **bold** font has been added and text with a ~~strikethrough~~ font has been deleted.

Version	Date	Changes
Version 4.0<br>Amendment 2.0	13 Dec 2012	**Protocol Synopsis – Objectives and 2.1.2 Secondary Objectives**<br><br>The secondary objectives of this study are<br>• **To determine the effect of rhBSSL treatment in decreasing the risk of growth restriction**<br>• **To determine the effect of rhBSSL treatment on neurodevelopment**<br>• **To determine the long-term effect of rhBSSL treatment on anthropometrics**<br>• **To determine the long-term safety of rhBSSL treatment**<br>• **To assess the effect of rhBSSL treatment on health care utilization**<br>• **To assess the effect of rhBSSL treatment on indirect resource use**<br>• **To assess the effect of rhBSSL treatment on chronic medical conditions**
		**Protocol Synopsis – Objectives and 2.2 Exploratory Objectives**<br><br>The exploratory objectives of this study are<br>• To assess the effect of rhBSSL treatment on fatty acid levels ~~(other than DHA and AA)~~
		**Protocol Synopsis - Study Design**<br><br>Follow-up visits will occur at 3 months after the first dose of study drug and at 12 **and 24** months corrected age. There will be an additional follow-up visit at 6 months after the first dose of study drug if rhBSSL antibodies are found at 3 months. Patients who have body composition assessments during the treatment period will also return for follow-up assessments at 40 weeks postmenstrual age and 3 months corrected age. **Telephone contacts will take place at 15, 18 and 21 months corrected age.**
		**Protocol Synopsis – Endpoints and 2.3.2 Secondary Endpoints**<br><br>The study has the following secondary efficacy endpoints:<br>• Body weight (g) at 12 **and 24** months corrected age<br>• **Body height (cm) at 24 months corrected age**<br>• **Growth restriction, defined as growth velocity <15 g per kilogram bodyweight per day during 4 weeks of treatment**<br>• Head circumference (mm) at 12 **and 24** months corrected age<br>• **Bayley Scale of Infant and Toddler Development III (Bayley-III) scores for the cognitive, language, motor, social-emotional and adaptive behavior domains at 24 months corrected age**<br>• **Neurodevelopment disability composite at 24 months corrected age**<br>• **Child Behavior Checklist (CBCL) scores at 24 months corrected age**<br>The study has the following secondary safety endpoints:

Version	Date	Changes
		• ~~Physical examination~~<br>• ~~Bayley Scale of Infant and Toddler Development~~ **Bayley**-III ~~(cognitive)~~ **scores for the cognitive, language and motor domains at 12 months corrected age**<br>• ~~Bayley Scale of Infant and Toddler Development III (language)~~<br>• ~~Bayley Scale of Infant and Toddler Development III (motor)~~<br>**The study has the following health economic endpoints:**<br>• **Number of outpatient visits and number of regular scheduled outpatient visits from the time of initial hospital discharge to home up to 24 months corrected age**<br>• **Number of inpatient visits, days of hospitalization and days in intensive care unit from the time of initial hospital discharge to home up to 24 months corrected age**<br>• **Number of days lost from work related to the child´s condition from the time of initial hospital discharge to home up to 24 months corrected age**<br>• **Presence of chronic medical conditions/diagnoses at 24 months corrected age**
		**Protocol Synopsis – Endpoints, 2.3.3 Exploratory Endpoints and 3.7.5 Exploratory Endpoints**<br><br>The study has the following exploratory endpoints:<br>• Levels of fatty acids~~, other than DHA and AA~~
		**Protocol Synopsis – Statistical Methods**<br><br>• Body weight (g) at 12 **and 24** months corrected age,<br>• **Body height (cm) at 24 months corrected age,**<br>• Head circumference (mm) at 12 **and 24** months corrected age<br>will be analyzed using an analysis of covariance approach similar to that used for the primary endpoint.<br>**The growth restriction will be analyzed using a logistic regression model with treatment, feeding regimen (PBM or infant formula), and size for gestational age category (SGA/AGA) as explanatory variables.**<br>**The Bayley-III cognition, language, motor, social-emotional and adaptive behavior composite scores at 24 months will be analyzed using an analysis of covariance model including factors for treatment, feeding regimen (PBM or infant formula), size for gestational age category (SGA or AGA), and critical confounders for neurodevelopment included as covariates.**<br>**The neurodevelopment disability composite scores will be analyzed using a logistic regression model with treatment, feeding regimen (PBM or infant formula), size for gestational age category (SGA or AGA) and critical confounders for neurodevelopment included as explanatory variables.**<br>The remaining endpoints, including secondary safety**, health economic** and exploratory endpoints, will be analyzed using descriptive statistics.

Version	Date	Changes
		**The study results will be reported after the last patient has conducted the 12-months-corrected-age visit for all assessments performed up to that point, with the exception of health economic assessments. The results of the 24-months-corrected-age assessments, as well as the health economic assessments and any serious adverse drug reactions, will be reported separately.**
		**List of Abbreviations**<br><br>**Bayley-III** **Bayley Scales of Infant and Toddler Development, third edition**<br><br>**CBCL** **Child Behavior Checklist**<br><br>**ADR** **adverse drug reaction**
		**1.6 Study Rationale**<br><br>**In addition, the patients will be followed up to 24 months corrected age to determine the long-term safety and effect (neurodevelopment and anthropometrics) of rhBSSL as well as assess the effect of rhBSSL on selected health economic variables.**
		**3.1 Study Design**<br><br>Follow-up visits will occur at 3 months after the first dose of study drug and at 12 **and 24** months corrected age. There will be an additional follow-up visit at 6 months after the first dose of study drug if rhBSSL antibodies are found at 3 months. Patients who have body composition assessments during the treatment period will also return for follow-up assessments at 40 weeks postmenstrual age and 3 months corrected age. **Telephone contacts will take place at 15, 18 and 21 months corrected age.**<br><br>**For patients that have conducted their 12-months-corrected-age visit before the implementation of amendment 2, i.e., before the long-term follow up was added, a separate visit will be conducted to collect informed consent, confounders for neurodevelopment, serious adverse drug reactions (ADRs) as well as perform the health economic assessments. This visit should take place as soon as the amendment has been approved by the regulatory authority and the independent ethics committee (IEC).**<br>**Figure 3-1 Study Schematic**<br><br>Figure 3-1 Study Schematic has been updated to reflect the changes made to the protocol as part of this amendment.<br><br>**Telephone contacts 15, 18 and 21 months corrected age**<br><br>**24 months corrected age**
		**3.4 Study Procedures**<br><br>**Informed consent will also be collected from the legally acceptable representative of the patients in the study for the 24-months-corrected-age assessments, as well as the health economic assessments and any serious ADRs. This consent will be collected when amendment 2 has been**

Version	Date	Changes
		**implemented and earliest at the 12-months-corrected-age visit. In addition, informed consent will be collected from the legally acceptable representatives for the collection of confounders for neurodevelopment.**<br><br>Patients will undergo the procedures at the time points specified in the schedule of events as shown in Table 6–1 **and Table 6–2**.
		**3.4.2 Demography Assessments and Recording of Confounders of Neurodevelopment**<br><br>**3.4.2.2 Confounders of Neurodevelopment**<br><br>**Even with randomization, confounding of intervention groups may occur when assessing developmental outcomes. Numerous factors related to child developmental outcome have been identified as confounding variables which need to be considered in randomized, controlled, trials of infant nutritional interventions. These variables include socioeconomic status, parental education, race, income, maternal IQ, age, psychological status, parity, marital status, child gender, number of children in the home and exposure to teratogenic substances (Singer 2001). In this study, confounders deemed to have a possible influence on the neurodevelopmental outcome have been selected.**<br><br>**The following confounders of neurodevelopment will be recorded as soon as informed consent has been provided (earliest at the 12-months-corrected-age visit):**<br><br>• **Medical and social factors of the biological mother**<br>○ **maternal height, weight and age at time of conception**<br>○ **presence of diabetes and/or preeclampsia during this pregnancy**<br>○ **nicotine use, alcohol intake and/or drug abuse during this pregnancy**<br>○ **number of previous pregnancies (all pregnancies that lasted beyond week 16 of gestation to be included)**<br><br>**If the biological mother is not the legally authorized representative, the above factors will not be collected.**<br><br>**The following confounders of neurodevelopment will be recorded at the 24-months-corrected-age visit:**<br><br>• **Factors of the household the patient currently lives in**<br>○ **number of children ≤18 years of age in the household**<br>○ **number of parents/primary caregivers in the household**<br>○ **socio economic status of parents/primary caregivers (educational level, employment status and income level)**

Version	Date	Changes
		**If the parent/primary caregiver is not the legally authorized representative, the above factors will not be collected.**
		**3.4.3.1 Body Weight**<br><br>Body weight at birth and the lowest measured body weight should be retrospectively recorded in the eCRF. The patient's body weight in grams will, as a minimum, be measured at Baseline (Day 1, before start of study drug administration), on Days 8, 15, 22, and 29, and on at least 2 other time points per week. Thereafter, body weight should be recorded at least weekly until discharge, and at each scheduled follow-up visit (Table 6–1 **and Table 6–2**). Body weight measured at any other time point from randomization up to discharge should also be recorded in the eCRF, but not more than 1 record per day.<br><br>During the treatment period, the body weight should be measured according to standard unit procedures, as near to the same time each day as is possible, and using the same scale (provided for the study), with graduation of 2 g, each time. The weight of catheters, tubes, and other items that cannot be removed should be subtracted from the total measured weight. Preferably the same equipment should also be used for assessments made after the treatment period, where possible.<br><br>**At the 24-months-corrected-age visit, the body weight does not need to be measured using the scale provided for the study. The body weight will be measured in accordance with hospital routine for children of this age.**
		**3.4.3.2 Total Body Length**<br><br>This measurement should be made at Baseline (Day 1), Days 8, 15, 22, and 29, at discharge, and at ~~each~~ **3-months and the 12-months-corrected-age** follow-up visits (Table 6–1 **and Table 6–2**). Measurements **up to discharge** should be taken as near to the same time each day as is possible.
		**3.4.3.3 Total Body Height**<br><br>**At the 24-months-corrected-age visit, the length measurement (lying) will be replaced by height measurement (standing). The body height will be measured in accordance with hospital routine for children of this age.**
		**3.4.3.4 Head Circumference**<br><br>This measurement should be made at Baseline (Day 1), Days 8, 15, 22, and 29, at discharge, and at each scheduled follow-up visit (Table 6–1 **and Table 6–2**). Measurements **up to discharge** should be taken as near to the same time each day as is possible.
		**3.4.3.7 Bayley Scales of Infant and Toddler Development**<br><br>**A description of the Bayley-III assessment is provided in Section 3.4.4.6.**
		**3.4.3.8 Child Behavior Checklist**<br><br>**The CBCL for 1.5 to 5 years of age is a parent-rated scale that will be used to evaluate mental health and behavioral development of children at 24 months corrected age. It consists of 99 problem items and one open-ended item for recording other problems not listed on the form. Each item will be rated by the parent/primary caregiver on a 0-2 point scale, where 0=not**

Version	Date	Changes
		**true, 1= somewhat or sometimes true and 2=very try or often true. Instructions on how to complete the CBCL will be given by a trained health care professional and the questionnaire should be completed at the site. For administrative reasons it is allowed to administer the CBCL on a different day than the main visit, as long as the administration takes place within the visit window specified in the schedule of events as shown in Table 6–2.**<br><br>**There are 7 syndrome subscales (emotionally reactive, anxious/depressed, somatic complaints, withdrawn, sleep problems, attention problems and aggressive behavior). These subscales are evaluated by summarizing the ratings for the items comprising each syndrome (Rescorla 2005). The subscales are also summarized as internalizing problems (emotionally reactive, anxious/depressed, somatic complaints and withdrawn items) and externalizing problems (attention problems and aggressive behavior items), and a total problem score is calculated.**<br><br>**The CBCL is part of the Achenbach System of Empirically Based Assessment (ASEBA) which has been used in several countries in research and clinical work (Rescorla 2005). The scale has good psychometric properties and confirmation of the reliability and validity of the problem scales have been confirmed for the Dutch version of the CBCL (Potijk et al 2012).**
		**3.4.3.9 Neurodevelopment Disability Composite**<br><br>**The neurodevelopment disability composite will be assessed at 24 months corrected age and is defined as presence of any one of the following:**<br>• **A composite score of less than 85 on any of the cognitive, language or motor domains of Bayley-III**<br>• **Bilateral deafness, defined as need for bilateral amplification**<br>• **Bilateral blindness, defined as corrected visual acuity of less than 20/200 (or equivalent) in the better eye**<br>• **Cerebral palsy (CP), defined as hypotonia, spastic diplegia, hemiplegia or quadriplegia causing functional deficits that require rehabilitation services**<br><br>**Similar neurodevelopment disability composites have been widely used to study neurodevelopmental outcome (Schmidt et al 2007; Wadhawan et al 2009; Wickremasinghe et al 2012).**
		**3.4.4.1.1 Definitions**<br><br>**Serious Adverse Drug Reaction**<br><br>**A serious ADR is defined as any event that fulfills the criteria for an SAE and where a causal relationship between the event and the study drug is suspected by the investigator.**
		**3.4.4.1.2 Eliciting and Documenting Adverse Events**<br><br>**Serious AEs:** SAEs will be reported from the time the informed consent has

Version	Date	Changes
		been signed **up to the 12-months-corrected-age** ~~until the patient's last~~ visit. ~~At every follow-up visit,~~ **T**he investigator will ask the patient's legally acceptable representative about any ongoing or previous (since last visit) events that fulfill the SAE criteria.<br><br>**Serious ADRs: Serious ADRs will be reported up to the 24-months-corrected-age visit. At the time of consent to the follow-up assessments (if not done at the 12-months-corrected-age visit), at telephone contacts and at the 24-months-corrected-age visit, the patient's legally acceptable representative will be asked about any ongoing or previous (since last contact or visit) events that fulfills the SAE criteria. The investigator will then judge if a causal relationship between the SAE and the study drug is suspected i.e., if the SAE is a serious ADR.**<br><br>**Any serious ADRs present at the 24-months-corrected-age visit should be followed up until satisfactory resolution or until the principal investigator or subinvestigator deems the event to be chronic or not clinically significant or the patient's condition to be stable, but without further recordings in the eCRF. Additional information received regarding patients with ongoing serious ADRs after 24 months should be reported via fax notification as described in Section 3.4.4.1.3.**
		**3.4.4.1.3 Reporting Adverse Events**<br><br>**Up to the 12-months-corrected-age visit, t**~~T~~he investigator's assessment of an AE's relationship to study drug is part of the documentation process, but it is not a factor in determining what is or is not reported in the study. If there is any doubt as to whether a clinical observation is an AE, the event will be reported. **Any SAE reported after the 12- and up to the 24-months-corrected-age visits must be assessed by the investigator regarding relationship to study drug and must if considered related (i.e. is a serious ADR) be reported in the study.**<br><br>**Serious Adverse Events** Any AE considered serious by the investigator or subinvestigator or which meets SAE criteria (Section 3.4.4.1.1) **occurring up to the 12-months-corrected-age visit, and any AE which meets SAE criteria (Section 3.4.4.1.1) and where a causal relationship between the event and the study drug is suspected by the investigator (serious ADR) occurring up to the 24-months-corrected-age visit,** must be reported to PPD Pharmacovigilance (PVG) Department within 24 hours from the time study center personnel first learn about the event. The following contact information should be used for SAE **and serious ADR** reporting (Fax line for sending the SAE notification and the hotline and e-mail for questions):<br><br>Fax line for sending the SAE **and serious ADR notification**: PPD PVG Fax line: +44 1223 374102<br><br>The SAE **and serious ADR** must be reported within 24 hours by completing the eCRF pages and sending a paper SAE notification fax to PVG. If the patient is hospitalized during the course of an SAE/**serious ADR**, or because of an SAE/ **serious ADR**, a copy of the hospital discharge summary should be faxed to PPD as soon as it becomes available.<br><br>**For SAEs occurring up to the 12-months-corrected-age visit,** PPD on behalf

Version	Date	Changes
		of Sobi is responsible for reporting unexpected fatal or life-threatening events associated with the use of the study drug (expedited reports) to the regulatory authorities, ethics committees, or principal investigators as required by fax, e-mail, or courier within 7 calendar days after being notified of the event. **For serious ADRs occurring after the 12-months-corrected-age visit, Drug Safety at Sobi will be responsible for reporting to appropriate regulatory authorities and PPD will be responsible for reporting to central ethics committees and principal investigators within the same time frames as above.**
		**3.4.4.1.6.1 Adverse Events Occurring before the 12-Months-corrected-age Visit**<br><br>All AEs (including SAEs) **occurring up to the 12-months-corrected-age visit,** captured in accordance with Section 3.4.4.1.2, must be followed to satisfactory resolution or until the principal investigator or subinvestigator deems the event to be chronic or not clinically significant or the patient's condition to be stable, but without further recordings into the eCRF after the 12-months-corrected-age visit. Follow-up information from SAEs that are ongoing at the 12-months-corrected-age visit will be reported via the fax notification as described in Section 3.4.4.1.3.<br><br>Sobi retains the right to request additional information for any patients with ongoing AE(s)/SAE(s) ~~at the end of the study~~ **at the 12-months-corrected-age visit**, if judged necessary.
		**3.4.4.1.6.2 Serious Adverse Drug Reactions Occurring after the 12–Months-corrected-age Visit**<br><br>**All serious ADRs occurring after the 12-months-corrected-age visit, captured in accordance with Section 3.4.4.1.2, must be followed to satisfactory resolution or until the principal investigator or subinvestigator deems the event to be chronic or not clinically significant or the patient's condition to be stable, but without further recordings into the eCRF after the 24-months-corrected-age visit. Follow-up information from SAEs that are ongoing at the 24-months-corrected-age visit will be reported via the fax notification as described in Section 3.4.4.1.3.**<br><br>**Sobi retains the right to request additional information for any patients with ongoing serious ADRs s at the 24-months-corrected-age visit, if judged necessary.**
		**3.4.4.2 Physical Examination**<br><br>Physical examination will be performed at Screening, Baseline, Days 8, 15, 22, and 29, at discharge, and at the follow-up visit**s** at 3 months and ~~the~~ 12 months corrected age ~~follow-up visit~~. Any clinically significant changes from Baseline **up to the 3-months visit** noted during the physical examinations should be reported as AEs. **See instructions regarding SAE/serious ADR reporting after this visit in Section 3.4.4.1.2.**
		**3.4.4.6 Bayley Scales of Infant and Toddler Development**<br><br>**The Bayley-III is an individually administered instrument that assesses the**

Version	Date	Changes
		**developmental functioning of infants and young children between 1 month and 42 months of age, across five domains: cognitive, motor, language, social-emotional, and adaptive behavior. Assessments of the cognitive, motor and language domains are conducted using items administered to the child; assessment of the social-emotional and adaptive behavior domains are conducted using parent/primary caregiver response to a questionnaire. Bayley-III is primarily used to identify young children with developmental delay and to assist health care providers in the intervention planning. The Bayley Scales of Infant and Toddler Development, second edition, has been used extensively in research to track the effects of intervention on children's development. The psychometric properties of the Bayley Scales of Infant and Toddler Development, second edition, have been maintained in Bayley-III (Bayley administration manual). In this study, Bayley-III** ~~The Bayley Scales of Infant and Toddler Development, third edition,~~ will be used to assess **neurodevelopment** ~~the patient at 12 months corrected age~~. ~~The study will assess t~~The cognitive, motor, and language ~~items from~~ **domains** ~~the Bayley Scale~~ will be **assessed at 12 and 24 months corrected age. At 24 months corrected age, the social-emotional and adaptive behavior domains will also be assessed.**<br><br>**The cognitive, motor, and language domains will be assessed by a trained health care professional. The questionnaires in the social-emotional and adaptive behavior domains will either be completed by the parents/primary caregivers or read to the parents/primary caregivers by a trained health care professional and the response from the parents/primary caregivers recorded. The social-emotional and adaptive behavior questionnaires may either be completed at the site or at home. Instructions on how to complete the questionnaires will be given by a trained health care professional. For administrative reasons it is allowed to administer and complete the Bayley-III on a different day than the main visit as long as the administration takes place within the visit window specified in the schedule of events as shown in Table 6–2.**<br><br>~~The results of the assessments should be recorded in the eCRF.~~ **A composite score will be calculated for each of the 5 domains, and scaled scores will be calculated for each subtest as well as for the cognitive and social-emotional domains.**<br><br>**At 24 months corrected age, the Bayley-III scores will be evaluated as secondary efficacy endpoints.**
		**3.4.4.7 Laboratory Safety Analyses**<br><br>When a routine safety sample is collected **during the treatment period**, the following should whenever possible be determined in those samples: amylase, aminotransferases (alanine aminotransferase and aspartate aminotransferase), total bilirubin, sodium, and urea.<br><br>**Information regarding laboratory values judged by the investigator to be abnormal and clinically significant will be captured in the eCRF up to the 3-months visit. Information will also be captured until the values return to normal.**
		**3.4.5 Exploratory Assessments**<br><br>**The data collected for exploratory assessments may not be evaluated as a part of the main clinical study report, but reported separately.**

Version	Date	Changes
		**3.4.5.1 Total Fatty Acids ~~(Other Than DHA and AA)~~**
		**3.4.5.2 Body Composition**<br>Body composition (percent fat mass), will be determined by air displacement plethysmography at study centers with access to the necessary equipment (Pea Pod®). Measurements will be performed at Baseline (Day 1), at the end of treatment **(**Day 29**)**, at 40 weeks postmenstrual age, and 3 months corrected age, with the 2 latter visit**s** scheduled only for patients with body composition assessments during the treatment period (Table 6–1 **and Table 6–2**).
		**3.4.6 Health Economic Assessments**<br>**3.4.6.1 Health Care Utilization**<br>**Information regarding health care utilization from the time of initial hospital discharge to home up to the 24-months-corrected-age visit will be collected through parent/primary caregiver reporting. The information will be collected as soon as informed consent has been provided (earliest at the 12-months-corrected-age visit), during telephone contacts at 15, 18 and 21 months corrected age and at the 24-months-corrected-age visit.**<br>**Parents/primary caregivers should be encouraged to use a notebook to keep track of all outpatient and inpatient visits and record information such as date, reason/diagnosis for visit, type of visit and duration of hospitalization. In case the caregiver is not able to provide information regarding health care utilization, the site will be asked to contact applicable health care providers for information. Approval from parents/primary caregivers to contact other health care providers will be collected as part of ICF procedure.**<br>**For outpatient care, the number of visits, type of health care provider, reason/diagnosis for visit and whether or not it was a regular scheduled visit will be recorded in the eCRF.**<br>**For inpatient care, the number of visits, reason/diagnosis for visit, number of days of hospitalization and number of days in the intensive care unit will be recorded in the eCRF.**<br>**The health care utilization information will not include standard preventive health care visits for children or vaccination programs.**
		**3.4.6.2 Indirect Resource Use**<br>**Information regarding number of days lost from work related to the child´s condition from the time of initial hospital discharge to home until the 24-months-corrected-age visit will be collected through parent/primary caregiver reporting. The information will be collected as soon as informed consent has been provided (earliest at the 12-months-corrected-age visit), during telephone contacts at 15, 18 and 21 months corrected age and at the 24 months corrected age visit.**<br>**The number of days lost from work related to the child´s condition will be recorded in the eCRF. Parents/caregivers should be encouraged to use a notebook to keep track of the number of days lost from work.**

Version	Date	Changes
		**3.4.6.3 Chronic Medical Conditions/Diagnoses**<br><br>**Chronic medical conditions/diagnoses present at 24 months corrected age will be recorded in the eCRF.**
		**3.6.4.3 Study Drug Accountability**<br><br>In addition, accurate records will be kept regarding when and how much study drug is ~~dispensed (ie,~~ added to food~~) and used by each patient in the study~~. The amount of drug ~~actually~~ administered to the patients will be ~~calculated~~ **estimated** from the food records (Section 3.6.5.1).
		**3.6.7.1 Emergency Unblinding**
		**3.6.7.2 Scheduled Unblinding**<br><br>**As further described in Section 5.8, the main clinical study report will be compiled after the last patient has completed the 12-months-corrected-age visit. The assessments performed at the 24-months-corrected-age visit, the health economic assessments and any serious ADRs will be reported separately at the end of the study. As a consequence, the blind will be broken for central functions both within PPD and Sobi for the analysis of the 12-month-corrected-age data before the last patient has conducted the last follow-up assessment. To minimize bias in the collection of data, the blind will be maintained for personnel at the sites and the patients legally authorized representatives as well as for local PPD staff until the end of the study.**
		**3.6.9 Prior, Concomitant, and Subsequent Therapy**<br><br>The patient is not allowed to participate concurrently in another clinical intervention study and as such may not use any drug or treatment concurrently under investigation **until the patient has completed the 12-months-corrected-age visit.**<br><br>Preterm infants often experience complications that need therapeutic intervention. This can in most cases be accepted as long as the medication does not interfere with feeding.<br><br>All use of concomitant and subsequent medications, from the start of the study up to the 3-month follow-up visit, will be recorded in the patient's eCRF (Table 6–1 **and Table 6–2**). This will include all prescription drugs, herbal products, vitamins, minerals, and over the counter medications. Any changes in concomitant medications also will be recorded in the patient's eCRF.
		**3.7.2 Secondary Efficacy Endpoints**<br><br>**The following secondary efficacy endpoint is evaluated to support the objective "To determine the effect of rhBSSL treatment in decreasing risk of growth restriction"**<br><br>• **Growth restriction, defined as growth velocity <15 g per kilogram bodyweight per day during 4 weeks of treatment**<br><br>**The following secondary efficacy endpoints will be evaluated to support the objective "To determine the effect of rhBSSL treatment on neurodevelopment":**<br><br>• **Bayley-III scores at 24 months corrected age**

Version	Date	Changes

- **Cognitive domain composite score**
- **Language domain composite score**
- **Motor domain composite score**
- **Social-emotional domain composite score**
- **Adaptive domain composite score**
- **Scaled scores for each subtest and for the cognitive and social-emotional domains (in total 16 scaled scores)**

- **Neurodevelopment disability composite at 24 months corrected age (as defined in section 3.4.3.9)**
- **CBCL scores at 24 months corrected age**
  - **Scores for each of 7 syndrome sub-scales (emotionally reactive, anxious/depressed, somatic complaints, withdrawn, sleep problems, attention problems and aggressive behavior)**
  - **Score for internalizing problems**
  - **Score for externalizing problems**
  - **Score for total problems**

The following secondary efficacy endpoints will be evaluated to support the objective "To determine the long-term effect of rhBSSL treatment on anthropometrics":

- **Body weight (g) at 24 months corrected age**
- **Body height (cm) at 24 months corrected age**
- **Head circumference (mm) at 24 months corrected age**

3.7.3 Safety Endpoints

The study has the following secondary safety endpoints to meet the objective "To compare the safety and tolerability of rhBSSL treatment in preterm infants with that of placebo treatment after oral administration in infant formula or PBM":

- AEs **up to 12 months corrected age**
- ~~Physical examination~~
- Bayley~~ Scale of Infant and Toddler Development -~~III ~~(cognitive)~~ **scores at 12 months corrected age**
  - **Cognitive domain composite score**
  - **Language domain composite score**
  - **Motor domain composite score**
  - **Scaled scores for each subtest and for the cognitive domain (in total 5 scaled scores)**
- ~~Bayley Scale of Infant and Toddler Development III (language)~~
- ~~Bayley Scale of Infant and Toddler Development III (motor)~~

The study has the following secondary safety endpoints to meet the objective "To determine the long-term safety of rhBSSL treatment":

- **Serious ADRs up to 24 months corrected age**

Version	Date	Changes
		**3.7.4 Health Economic Endpoints**<br><br>**The study has the following health economic endpoints to meet the objective "To assess the effect of rhBSSL treatment on health care utilization":**<br>• **The number of outpatient visits and the number of regular scheduled outpatient visits from the time of initial hospital discharge to home up to 24 months corrected age**<br>• **The number of inpatient visits, days of hospitalization and days in intensive care unit from the time of initial hospital discharge to home up to 24 months corrected age**<br>**The study has the following endpoint to meet the objective "To assess the effect of rhBSSL treatment on indirect resource use":**<br>• **Number of days lost from work related to the child´s condition from the time of initial hospital discharge to home up to 24 months corrected age**<br>**The study has the following endpoint to meet the objective "To assess the effect of rhBSSL treatment on chronic medical conditions/diagnoses":**<br>• **Presence of chronic medical conditions/diagnoses at 24 months corrected age**
		**3.7.5 Exploratory Endpoints**<br><br>The study has the following exploratory endpoints:<br>• Levels of fatty acids~~, other than DHA and AA~~
		**3.8.2 Secondary Efficacy Analyses**<br><br>**The growth restriction is defined as a growth velocity of less than 15 g per kilogram bodyweight per day during the 4-week treatment period and will be analyzed using a logistic regression model with treatment, feeding regimen (PBM or infant formula), and size for gestational age category (SGA/AGA) as explanatory variables. Based on this model, the estimated risk of growth restriction in the rhBSSL and placebo groups respectively, the estimated odds ratio, the associated 95% confidence interval and P-value will be presented. If the risk of growth restriction in the rhBSSL group is statistically significantly lower than in the placebo group, it will be concluded that rhBSSL reduces the risk of growth restriction in preterm infants receiving infant formula or PBM. In addition, the number and proportion of patients in each treatment group with growth restriction will be presented for all patients as well as by feeding regimen and by size for gestational age category.**<br>**Statistical analysis of the Bayley-III cognition, language, motor, social-emotional and adaptive behavior composite scores at 24 months corrected age will be performed using an analysis of covariance model including factors for treatment, feeding regimen (PBM or infant formula), size for gestational age category (SGA or AGA), and critical confounders for neurodevelopment included as covariates. Based on this model, the estimated mean score for each treatment group, the estimated difference between treatments and the associated 95% confidence interval will be presented. The critical confounders to be included in the model will be specified in a study-specific statistical analysis plan finalized prior to**

Version	Date	Changes
		**breaking the blind. In addition, the respective domain composite score and the scaled score for each subtest at 24 months corrected age will be presented by descriptive statistics.**<br><br>**The neurodevelopment disability composite outcome will be analyzed using a logistic regression model including treatment, feeding regimen (PBM or infant formula), size for gestational age category (SGA or AGA) and critical confounders for neurodevelopment included as explanatory variables. Based on this model, the estimated risk of a neurodevelopment disability in each treatment group, the estimated odds ratio for rhBSSL to placebo and the associated 95% confidence interval will be presented. The critical confounders to be included in the model will be specified in a study-specific statistical analysis plan finalized prior to breaking the blind. Furthermore, the number and proportion of patients in each treatment group with a neurodevelopment disability (according to the above scale) will be presented for all patients as well as by feeding regimen and by size for gestational age category. This latter presentation will also be done for each individual component of the composite for all patients and by feeding regimen and by size for gestational age category.**<br><br>**The CBCL scores will be analyzed using descriptive statistics.**
		**3.8.3 Health Economic Endpoints**<br><br>**Health economic endpoints will be analyzed using descriptive statistics.**
		**3.8.6 Safety Analyses**<br><br>Safety analysis will be performed for vital sign measurements, laboratory safety results, tolerability, ECG findings, vitamin A and D levels, antibodies against rhBSSL, Bayley-III **(composite scores for the cognitive, language and motor domains at 12 months corrected age),** ~~physical examination,~~ and AE data **collected up to 12 months corrected age**.<br><br>**A separate safety analysis will be performed for serious ADRs collected up to 24 months corrected age.**
		**3.8.6.1 Adverse Events**<br><br>The incidence of SAEs, including death**, serious ADRs** and AEs leading to study drug discontinuation will also be tabulated.
		**3.8.6.2 Clinical Laboratory Results**<br><br>The number and proportion of patients experiencing clinically significant events ~~at 4 weeks and across the duration of the study~~ **during the treatment period** will be presented.
		**3.8.8 Analysis of 12-months Data**<br><br>**The main analysis will be performed when the last patient has completed the 12-months-corrected-age visit. The results of this analysis will be reported in the main clinical study report.**
		**3.8.9 Analysis of 24-months Data**<br><br>**The analysis of the 24-months-corrected-age assessments, as well as the health economic assessments and any serious ADRs will be performed when the last patient has completed the 24-months-corrected-age visit. The**

Version	Date	Changes
		**results of this analysis will be reported a separate clinical study report.**
		**3.8.10 Combining Data**<br><br>**A prospective protocol with the purpose of combining data from this study with data from other possible future studies will, if applicable, be written.**
		**4.3 Informed Consent**<br><br>**Informed consent will also be collected from the patient´s legally acceptable representative for the 24-months-corrected-age assessments, as well as the health economic assessments and any serious ADRs. This consent will be collected when amendment 2 has been implemented and earliest at the 12-months-corrected-age visit. In addition, informed consent will be collected from the legally acceptable representatives for the collection of confounders of neurodevelopment.**<br>**If consent to continue into the extension study is refused then data will be collected as per the original protocol, up to and including the 12-months-corrected-age visit. The patient will follow the end of study procedures as described in the original protocol and no further data will be collected.**<br>Information will be given in both oral and written form. The informed consent information sheet**s** will include all the elements required by law following the ICH E6(R1) guidelines. The informed consent**s** will be approved by the IEC (and regulatory authorities) of each study center.
		**4.10 Coding Dictionaries**<br><br>Medical history, **chronic medical conditions/diagnoses** as well as all AEs, will be coded using MedDRA.
		**4.13 Publications**<br><br>After completion of the study, the data may be considered for reporting at ~~a~~ scientific meeting**s** or for publication**s** in ~~a~~ scientific journal**s**.
		**5.8 Final Report**<br><br>**The results of the assessments performed up to the 12-months-corrected-age visit will be presented in the main clinical study report after the last patient has completed the 12-months-corrected-age visit. The 24-months-corrected-age assessments, the health economic assessments and any serious ADRs, will be reported in a separate clinical study report when all patients have completed the study.**<br>Where required by applicable regulatory requirements, ~~an investigator signatory will be identified for the approval of~~ the clinical study report **will be approved and signed by the international coordinating investigator**. The **international coordinating** investigator will be provided reasonable access to statistical tables, figures, and relevant reports and will have the opportunity to review the complete study results.<br>Upon completion of the **study and reporting of 24-months-corrected-age data**~~clinical study report~~, the sponsor will provide the investigator**s** with the full summary of the study results. The investigator**s are** ~~is~~ encouraged to share the summary results with the study patients' legally acceptable representatives, as appropriate.

Version	Date	Changes
		**Appendix 1 Table 6-1 Schedule of Events until Discharge**<br><br>The previous Table 6-1 has been divided into two tables (Table 6–1 and Table 6–2) to increase the readability.<br><br>Table 6–1 has been updated to reflect all the changes made to the protocol as part of this amendment.<br><br>Footnote d: When a routine safety sample is collected **during the treatment period**, the following should, whenever possible, be determined in those samples: amylase, aminotransferases (alanine aminotransferase and aspartate aminotransferase), total bilirubin, sodium, and urea. If no routine samples are collected during Days 14 to 29, one sample must be collected for the analysis of amylase, aminotransferases, total bilirubin, sodium, and urea. **Information regarding laboratory values judged by the investigator to be abnormal and clinically significant will be captured in the electronic case report form up to the 3-months visit. Information will also be captured until the values return to normal.**<br><br>Footnote e: The patient's body weight in grams will, as a minimum, be measured at Baseline (Day 1, before start of study drug administration), on Days 8, 15, 22, and 29, and on at least 2 other time points per week. Thereafter, body weight should be recorded at least weekly until discharge~~, and at each scheduled follow-up visit~~. Body weight measured at any other time point from randomization up to discharge should also be recorded in the electronic case report form.<br><br>Footnote i: **All s**erious **adverse events** ~~AEs~~ will be reported from the time the informed consent has been signed **and up to the 12-months-corrected-age visit**.
		**Appendix 1 Table 6-2 Schedule of Events for Follow-up Visits**<br><br>Table 6–2 has been updated to reflect all the changes made to the protocol as part of this amendment.<br><br>Footnote a: **The 40 weeks postmenstrual age and the 3-months-corrected-age visits will only occur for patients with body composition assessments during the treatment period.**<br><br>Footnote c: **Will only occur if informed consent for the scheduled assessments has been provided.**<br><br>Footnote d: **For the 24-months-corrected-age assessments, confounders of neurodevelopment, health economic assessments and any serious adverse drug reactions. Can be done at a later time point for patients that have conducted the 12-months-corrected-age visit before the implementation of amendment 2.**<br><br>Footnote e: **Information regarding laboratory values judged by the investigator to be abnormal and clinically significant will be captured in the electronic caser report form up to the 3-months visit. Information will also be captured until the values return to normal.**<br><br>Footnote g: **Measured as height (cm) at 24 months corrected age.**<br><br>Footnote i: **As soon as informed consent has been provided (earliest at the 12-months-corrected-age visit).**<br><br>Footnote j: **All s**erious **adverse events** ~~AEs~~ will be reported from the time the informed consent has been signed **and up to the 12-months-corrected-age**

Version	Date	Changes
		**visit**. Adverse events (including monitoring of infections, necrotizing enterocolitis, sepsis, intraventricular hemorrhage, retinopathy of prematurity, hearing impairment, bronchopulmonary dysplasia, and cholestasis) will be recorded from the start of treatment on Day 1 until the 3-month follow-up visit. **Serious adverse drug reactions will be reported up to 24 months corrected age.**<br><br>Footnote l: **The patient's legally acceptable representative will be asked about any ongoing or previous (since last contact or visit) events that fulfills the serious adverse event criteria. The investigator will then judge if a causal relationship between the serious adverse event and the study drug is suspected i.e., if the serious adverse event is a serious adverse drug reaction.**
		**7. Reference List**<br><br>**Bayley, N. Administration Manual for the Bayley Scales of Infant and Toddler Development Third Edition. San Antonio, TX: Pearson; 2006.**<br><br>**Potijk MR, de Winter AF, Bos AF, et al. Higher rates of behavioural and emotional problems at preschool age in children born moderately preterm. Arch Dis Child 2012;97:112-7.**<br><br>**Rescorla LA. Assessment of young children using the achenbach system of empirically based assessment (ASEBA). Ment Retard Dev D R 2005;11 :226-37.**<br><br>**Schmidt B, Roberts RS, Davis P, et al. Long-term effects of caffeine therapy for apnea of prematurity. N Engl J Med 2007;357:1893-1902.**<br><br>**Singer L.T. Randomized clinical trials in infancy: methodologic issues. Semin Neonatol 2001;6:393-401.**<br><br>**Vohr BR, O´Shea M, Wright LL. Longitudinal multicenter follow-up of high-risk infants: why, who, when, and what to assess. Semin Perinatol. 2003;27(4):333-42.**<br><br>**Wadhawan R, Oh W, Perritt RL, et al. Twin gestation and neurodevelopmental outcome in extremely low birth weight infants. Pediatrics 2009;123:e220-27.**<br><br>**Wickremasinghe AC, Rogers EE, Piecuch RE, et al. Neurodevelopmental outcomes following two different treatment approaches (early ligation and selective ligation) for patent ductus arteriosus. J Pediatr 2012 (Epub ahead of print).**

Appendix 5: Administrative Change 2.0

Text with a **bold** font has been added and text with a ~~strikethrough~~ font has been deleted.

Version	Date	Changes
Version 1.0	10 Feb 2011	Version 1.0
Version 2.0<br>Amendment 1.0	27 May 2011	See Appendix 2
Version 3.0<br>Administrative Change 1.0	14 May 2012	See Appendix 3
Version 4.0<br>Amendment 2.0	13 Dec 2012	See Appendix 4
Version 5.0<br>Administrative Change 2.0	10 Jun 2013	**Title Page and Protocol Approval Signature Page**<br><br>**Sponsor's Medical Monitor:** ~~Kristina Timdahl MD~~**Björn Paulsson MD**
		**Protocol Synopsis – health economic endpoints**<br><br>The study has the following secondary safety endpoints:<br>• ~~Levels~~**Presence** of rhBSSL antibodies<br>The study has the following health economic endpoints:<br>• Number of ~~outpatient visits and number of regular scheduled~~ outpatient visits from the time of initial hospital discharge to home up to 24 months corrected age
		**Protocol Synopsis – statistical methods**<br><br>Time to readiness for discharge **and time to discharge** will be analyzed using ~~Cox proportional hazards models~~**an analysis of variance model**.<br>**Readmission to hospital within 1 month of discharge and** ~~The~~ growth restriction will be analyzed using a logistic regression model with treatment, feeding regimen (PBM or infant formula), and size for gestational age category (SGA/AGA) as explanatory variables.<br>The study results will be reported after ~~the last patient has conducted~~ **200 patients have completed** the 12-months-corrected-age visit ~~for all assessments performed up to that point, with the exception of health economic assessments,~~ **including the Bayley assessment. A separate addendum to the report will be prepared when the remaining patients have completed the 12-month visit.**
		**2.3.2 Secondary Endpoints**<br><br>The study has the following secondary safety endpoints:<br>• ~~Levels~~**Presence** of rhBSSL antibodies<br><br>The study has the following health economic endpoints:<br>• Number of ~~outpatient visits and number of regular scheduled~~ outpatient visits from the time of initial hospital discharge to home up

Version	Date	Changes
		to 24 months corrected age
		**3.1 Study Design**<br><br>~~As soon as~~ **When** patients reach a level of enteral feeding of **at least** 100 mL/kg/day, they will be randomly assigned to receive rhBSSL 8700 U or placebo added to 100 mL of the food.
		**3.4.2.1 Demography Assessments**<br><br>• **Information related to multiple birth**
		**3.4.3.6 Docosahexaenoic Acid (DHA) and Arachidonic Acid (AA)**<br><br>Section ~~0~~**3.4.7** provides further details of sampling.
		**3.4.4.7.1 Vitamins A and D**<br><br>Section ~~0~~**3.4.7** provides further details of sampling.
		**3.4.4.7.2 rhBSSL Antibodies**<br><br>Section ~~0~~ **3.4.7** provides further details of sampling.
		**3.4.6.1 Health Care Utilization**<br><br>For outpatient care, the number of visits~~,~~ **and** type of health care provider~~, reason/diagnosis for visit and whether or not it was a regular scheduled visit~~ will be recorded in the eCRF.
		**3.6.7.2 Scheduled Unblinding**<br><br>~~As further described in Section 5.8, the main clinical study report will be compiled after the last patient has completed the 12-months-corrected-age visit. The assessments performed at the 24-months-corrected-age visit, the health economic assessments and any serious ADRs will be reported separately at the end of the study. As a consequence, the blind will be broken for central functions both within PPD and Sobi for the analysis of the 12-month-corrected-age data before the last patient has conducted the last follow-up assessment. To minimize bias in the collection of data, the~~ **The blind will be broken for an independent analysis and reporting team within PPD and for Sobi once the last patient has completed the 3-month visit and 200 patients have completed the 12-months-corrected-age visit, including the Bayley assessment. Once unblinding has occurred, the unblinded teams will not be involved in decisions regarding individual patients where the patient identity can be revealed. The** blind will be maintained for all **other teams within PPD,** personnel at the sites**,** and the patients**'** legally authorized representatives ~~as well as for local PPD staff~~ until the end of the study. **For details of study reporting, see Section 5.8.**
		**3.7.2 Secondary Efficacy Endpoints**<br><br>The following secondary efficacy endpoint ~~is~~ **will be** evaluated to support the objective "To determine the effect of rhBSSL treatment in decreasing risk of growth restriction"<br><br>The following secondary efficacy endpoints ~~are~~**will be** evaluated to support the objective "To determine the effect of rhBSSL treatment in shortening the time

Version	Date	Changes
		of hospital stay"
		**3.7.3 Safety Endpoints**<br>• AEs up to **3 months and SAEs up to** 12 months corrected age<br>• ~~Levels~~ **Presence** of rhBSSL antibodies
		**3.7.4 Health Economic Endpoints**<br>• The number of ~~outpatient visits and the number of regular scheduled~~ outpatient visits from the time of initial hospital discharge to home up to 24 months corrected age
		**3.8.2 Secondary Efficacy Analyses**<br>**The following will be analyzed using descriptive statistics:**<br>• **Body weight (g) at 24 months corrected age**<br>• **Body height (cm) at 24 months corrected age**<br>• **Head circumference (mm) at 24 months corrected age**<br>• **Weight-to-length ratio (g/cm) at 4 weeks**<br>~~Time to readiness for discharge will be analyzed using Cox proportional hazards models. Time will be calculated from date of first dose. Patients who do not experience an event will be censored at their last available visit. The models will be stratified by feeding regimen (PBM or infant formula), and the size for gestational age category (SGA or AGA). Treatment effect will be estimated using the hazard ratio and corresponding 95% confidence interval. Unstratified Kaplan Meier estimates of event free proportion will also be calculated for graphical presentation of each endpoint by treatment.~~ **Time to readiness for discharge will be analyzed using an analysis of variance model. Time to readiness for discharge will be used as the response variable and factors will be included for treatment, feeding regimen (PBM or infant formula) and size for gestational age category (SGA or AGA). For the comparison between treatment groups, the point estimate, the associated 95% confidence interval and P-value from the model will be presented.**<br>**Time to discharge will be analyzed using an analysis of variance model similar to that used for time to readiness for discharge.**<br>**Readmission to hospital within 1 month of discharge will be analyzed using a logistic regression model with treatment, feeding regimen (PBM or infant formula), and size for gestational age category (SGA/AGA) as explanatory variables. Based on this model, the estimated risk of readmission to hospital in the rhBSSL and placebo groups respectively, the estimated odds ratio and the 95% confidence interval will be presented. In addition, the number and proportion of patients in each treatment group who were readmitted to hospital will be presented for all patients as well as by feeding regimen and by size for gestational age category.**<br>Time from start of treatment to 150 mL/kg/day of enteral feeding ~~and time to discharge and readmittance to hospital within 1 month from discharge~~ will be presented using descriptive statistics.
		**3.8.6 Safety Analysis**<br>Safety analysis will be performed for vital sign measurements, laboratory safety

Version	Date	Changes
		results, tolerability, **withheld enteral feeding during 24 hours,** ECG findings, vitamin A and D levels, antibodies against rhBSSL, Bayley-III (composite scores for the cognitive, language and motor domains at 12 months corrected age), and AE data collected up to **3 months and SAE data up to** 12 months corrected age.
		**3.8.6.1 Adverse Events**<br><br>All treatment-emergent AEs will be summarized showing the number of patients who experienced an event, the percentage of patients with that event, and the ~~grade~~**severity**, duration, and relationship to study drug.
		**3.8.8 Analysis of Data Up to 12 Months ~~Data~~**<br><br>~~The main analysis will be performed~~ **All available data up to the 12-months-corrected-age-visit will be analyzed** when ~~the last patient has~~ **all patients have completed the 3-month visit and 200 patients have** completed the 12-months-corrected-age visit**, including the Bayley assessment.** The results ~~of this analysis~~ will be reported in ~~the main~~ **a** clinical study report. **When all patients have completed their 12-month visit, complementary analyses of 12-month data (ie, after the 3-month visit and up to the 12-month visit) will be performed and reported in an addendum to the clinical study report.**
		**3.8.9 Analysis of 24-months Data**<br><br>The results of this analysis will be reported **in** a separate clinical study report.
		**5.8 Final Report**<br><br>The results of the assessments ~~performed up to the 12-months-corrected-age visit will be presented in the main clinical study report after the last patient has completed the 12-months-corrected-age visit~~ **of all patients that have completed the 3-month visit and 200 patients that have completed the 12-months-corrected-age visit, including the Bayley assessment, will be presented in a clinical study report. A separate addendum to the report will be prepared when the remaining patients have completed the 12-month visit.** The 24-months-corrected-age assessments, the health economic assessments, and any serious ADRs~~,~~ will be reported in a separate clinical study report when all patients have completed the study. **Exploratory endpoints may be reported separately.**

7 Reference List

Andersson Y, Savman K, Blackberg L, et al. Pasteurization of mother's own milk reduces fat absorption and growth in preterm infants. Acta Paediatr. 2007;96(10):1445-9.

Armand M. Lipases and lipolysis in the human digestive tract: where do we stand? Curr Opin Clin Nutr Metab Care. 2007;10(2):156-64.

Bayley, N. Administration Manual for the Bayley Scales of Infant and Toddler Development Third Edition. San Antonio, TX: Pearson; 2006.

Bloom BT, Mulligan J, Arnold C, et al. Improving growth of very low birth weight infants in the first 28 days. Pediatrics. 2003;112(1 Pt 1):8-14.

Carey M, Hernell O. Digestion and absorption of fat. Semin Gastrointest Dis. 1992;3:189-208.

Casey PH. Growth of low birth weight preterm children. Semin Perinatol. 2008;32(1):20-7.

Dusick AM, Poindexter BB, Ehrenkranz RA, et al. Growth failure in the preterm infant: can we catch up? Semin Perinatol. 2003;27(4):302-10.

Ehrenkranz RA, Younes N, Lemons JA, et al. Longitudinal growth of hospitalized very low birth weight infants. Pediatrics. 1999;104(2 Pt 1):280-9.

Ehrenkranz RA, Dusick AM, Vohr BR, et al. Growth in the neonatal intensive care unit influences neurodevelopmental and growth outcomes of extremely low birth weight infants. Pediatrics. 2006;117(4):1253-61.

Embleton NE, Pang N, Cooke RJ. Postnatal malnutrition and growth retardation: an inevitable consequence of current recommendations in preterm infants? Pediatrics. 2001;107(2):270-3.

Engle WA; American Academy of Pediatrics Committee on Fetus and Newborn. Age terminology during the perinatal period. Pediatrics. 2004;114(5):1362-4.

Fredrikzon B, Hernell O, Blackberg L, et al. Bile salt-stimulated lipase in human milk: evidence of activity in vivo and of a role in the digestion of milk retinol esters. Pediatr Res. 1978;12(11):1048-52.

Freed LM, Neville MC, Hamosh P, et al. Diurnal and within-feed variations in lipase activity and triglyceride content of human milk. J Pediatr Gastroenterol Nutr. 1986;5(6):938-42.

Hamosh M. Lipases: digestion of neutral lipids; physiological and pathological implications. [cited 11 Nov 2010]. In: Encyclopedia of Life Sciences [Internet]. Hoboken (NJ): Wiley & Sons Inc. 2005. Available from: http://onlinelibrary.wiley.com.

Hawcutt DB, Rose AC, Fuerst-Recktenwald S, et al. Points to consider when planning the collection of blood or tissue samples in clinical trials of investigational medicinal products in children, infants and neonates. Guide to paediatric drug development and clinical research. Basel, Karger; 2010. 97-110.

Hernell O, Gebre-Medhin M, Olivecrona T. Breast milk composition in Ethiopian and Swedish mothers. IV. Milk lipases. Am J Clin Nutr. 1977;30(4):508-11.

Lebenthal E, Lee PC. Development of functional responses in human exocrine pancreas. Pediatrics. 1980;66(4):556-60.

Lombardo D. Bile salt-dependent lipase: its pathophysiological implications. Biochim Biophys Acta. 2001;1533(1):1-28.

Manson WG, Weaver LT. Fat digestion in the neonate. Arch Dis Child Fetal Neonatal Ed. 1997;76(3):F206-11.

Olsen IE, Groveman SA, Lawson ML, et al. New intrauterine growth curves based on United States data. Pediatrics. 2010;125(2):e214-24.

Potijk MR, de Winter AF, Bos AF, et al. Higher rates of behavioural and emotional problems at preschool age in children born moderately preterm. Arch Dis Child 2012;97:112-7.

Rescorla LA. Assessment of young children using the achenbach system of empirically based assessment (ASEBA). Ment Retard Dev D R 2005;11 :226-37.

Rouge C, Butel MJ, Piloquet H, et al. Fecal calprotectin excretion in preterm infants during the neonatal period. PLoS ONE. 2010;5(6):e11083.

Savino F, Castagno E, Calabrese R, et al. High faecal calprotectin levels in healthy, exclusively breast-fed infants. Neonatology 2010;97(4):299-304.

Schmidt B, Roberts RS, Davis P, et al. Long-term effects of caffeine therapy for apnea of prematurity. N Engl J Med 2007;357:1893-1902.

Singer L.T. Randomized clinical trials in infancy: methodologic issues. Semin Neonatol 2001;6:393-401.

Stromqvist M, Hernell O, Hansson L, et al. Naturally occurring variants of human milk bile salt-stimulated lipase. Arch Biochem Biophys. 1997;347(1):30-6.

Torres JE, VanderJagt D, Okolo SN, et al. Bile salt-stimulated lipase in the milk of Fulani and Kanuri women in Nigeria and native Nepalese women. J Natl Med Assoc. 2001;93(6):201-7.

Vohr BR, O´Shea M, Wright LL. Longitudinal multicenter follow-up of high-risk infants: why, who, when, and what to assess. Semin Perinatol. 2003;27(4):333-42.

Wadhawan R, Oh W, Perritt RL, et al. Twin gestation and neurodevelopmental outcome in extremely low birth weight infants. Pediatrics 2009;123:e220-27.

Wager E, Field EA, Grossman L. Good publication practice for pharmaceutical companies. Curr Med Res Opin 2003;19(3):149-54.

Wickremasinghe AC, Rogers EE, Piecuch RE, et al. Neurodevelopmental outcomes following two different treatment approaches (early ligation and selective ligation) for patent ductus arteriosus. J Pediatr 2012 (Epub ahead of print).

Williamson S, Finucane E, Ellis H, et al. Effect of heat treatment of human milk on absorption of nitrogen, fat, sodium, calcium, and phosphorus by preterm infants. Arch Dis Child. 1978;53(7):555-63.

Zoppi G, Andreotti G, Pajno-Ferrara F, et al. Exocrine pancreas function in premature and full term neonates. Pediatr Res. 1972;6:880-6.
